# Growth control of the eukaryote cell: a systems biology study in yeast

**DOI:** 10.1186/jbiol54

**Published:** 2007-04-30

**Authors:** Juan I Castrillo, Leo A Zeef, David C Hoyle, Nianshu Zhang, Andrew Hayes, David CJ Gardner, Michael J Cornell, June Petty, Luke Hakes, Leanne Wardleworth, Bharat Rash, Marie Brown, Warwick B Dunn, David Broadhurst, Kerry O'Donoghue, Svenja S Hester, Tom PJ Dunkley, Sarah R Hart, Neil Swainston, Peter Li, Simon J Gaskell, Norman W Paton, Kathryn S Lilley, Douglas B Kell, Stephen G Oliver

**Affiliations:** 1 Faculty of Life Sciences, Michael Smith Building, University of Manchester, Oxford Road, Manchester M13 9PT, UK; 2 Northwest Institute for Bio-Health Informatics (NIBHI), School of Medicine, Stopford Building, University of Manchester, Oxford Road, Manchester M13 9PT, UK; 3 School of Computer Science, Kilburn Building, University of Manchester, Oxford Road, Manchester M13 9PL, UK; 4 School of Chemistry, Manchester Interdisciplinary Biocentre, University of Manchester, 131 Princess St, Manchester M1 7DN, UK; 5 Cambridge Centre for Proteomics, Department of Biochemistry, University of Cambridge, Downing Site, Cambridge CB2 1QW, UK; 6 Manchester Centre for Integrative Systems Biology, Manchester Interdisciplinary Biocentre, University of Manchester, 131 Princess St, Manchester M1 7DN, UK

## Abstract

**Background:**

Cell growth underlies many key cellular and developmental processes, yet a limited number of studies have been carried out on cell-growth regulation. Comprehensive studies at the transcriptional, proteomic and metabolic levels under defined controlled conditions are currently lacking.

**Results:**

Metabolic control analysis is being exploited in a systems biology study of the eukaryotic cell. Using chemostat culture, we have measured the impact of changes in flux (growth rate) on the transcriptome, proteome, endometabolome and exometabolome of the yeast *Saccharomyces cerevisiae*. Each functional genomic level shows clear growth-rate-associated trends and discriminates between carbon-sufficient and carbon-limited conditions. Genes consistently and significantly upregulated with increasing growth rate are frequently essential and encode evolutionarily conserved proteins of known function that participate in many protein-protein interactions. In contrast, more unknown, and fewer essential, genes are downregulated with increasing growth rate; their protein products rarely interact with one another. A large proportion of yeast genes under positive growth-rate control share orthologs with other eukaryotes, including humans. Significantly, transcription of genes encoding components of the TOR complex (a major controller of eukaryotic cell growth) is not subject to growth-rate regulation. Moreover, integrative studies reveal the extent and importance of post-transcriptional control, patterns of control of metabolic fluxes at the level of enzyme synthesis, and the relevance of specific enzymatic reactions in the control of metabolic fluxes during cell growth.

**Conclusion:**

This work constitutes a first comprehensive systems biology study on growth-rate control in the eukaryotic cell. The results have direct implications for advanced studies on cell growth, *in vivo *regulation of metabolic fluxes for comprehensive metabolic engineering, and for the design of genome-scale systems biology models of the eukaryotic cell.

## Background

Metabolic control analysis [1] is a conceptual and mathematical formalism that models the relative contributions of individual effectors in a pathway to both the flux through the pathway and the concentrations of intermediates within it. To exploit metabolic control analysis in an initial systems biology analysis of the eukaryotic cell, two categories of experiments are required. In category 1, flux is changed and the impact on the levels of the direct and indirect products of gene action is measured. In category 2, the levels of individual gene products are altered, and the impact on the flux is measured. In this category 1 study, we have measured the impact of changing the flux on the transcriptome, proteome, and metabolome of *Saccharomyces cerevisiae*. In this whole-cell analysis, flux equates to growth rate.

Cell growth (the increase in cell mass through macromolecular synthesis) requires the synthesis of cellular components in precise, stoichiometric quantities, and must be subject to tight coordinate control [2-6]. Cell growth underpins many critical cellular and developmental processes, yet comprehensive studies on growth rate and its control have lagged behind those on cell-cycle progression [7,8], cell proliferation [4,6] and coupling between cell growth and division [9,10]. A limited number of studies in batch (flask) cultures in complex media have been reported for the important model eukaryote *Saccharomyces cerevisiae*. These showed that the coordinate expression of ribosomal protein genes with growth rate appeared regulated almost entirely at the transcriptional level [11-13]. However, these batch studies could not separate growth rate from nutritional effects [14]. Chemostat cultures in defined media constitute an adequate alternative, allowing the study of physiological patterns under controlled environmental conditions [14-17]. However, the majority of chemostat studies have mainly focused on the characterization of environmental responses at a single growth rate [18-20], and so the mechanisms involved in the regulation of growth-rate-related genes are still poorly understood. Previous investigations have been confined to the RNA level; however, an increasing number of studies demonstrate the importance of post-transcriptional (translational and post-translational) mechanisms [21-24]. This evidence for control being exerted at multiple levels emphasizes the need to extend metabolic control analysis to include the concept of modular control [25].

Comprehensive high-throughput analyses at the levels of mRNAs, proteins, and metabolites, and studies on gene expression patterns are required for systems biology studies of cell growth [4,26-29]. Although such comprehensive data sets are lacking, many studies have pointed to a central role for the target-of-rapamycin (TOR) signal transduction pathway in growth control. TOR is a serine/threonine kinase that has been conserved from yeasts to mammals; it integrates signals from nutrients or growth factors to regulate cell growth and cell-cycle progression coordinately [3,30-33].

We have studied the control of the yeast transcriptome, proteome, and metabolome in a manner that allows the separation of growth-rate effects from nutritional effects, and have paid particular attention to the role of the rapamycin-sensitive TOR complex 1 (TORC1) [32] in mediating growth-rate control. Both the concepts and the data generated by these experiments should provide a useful foundation for the construction of dynamic models of the yeast cell in systems biology [26-28].

## Results and discussion

### Growth-rate effects revealed at all 'omic' levels

We wished to study the impact of growth rate on the total complement of mRNA molecules, proteins, and metabolites in *S. cerevisiae*, independent of any nutritional or other physiological effects. To achieve this, we carried out our analyses on yeast grown in steady-state chemostat culture under four different nutrient limitations (glucose, ammonium, phosphate, and sulfate) at three different dilution (that is, growth) rates (*D *= μ = 0.07, 0.1, and 0.2/hour, equivalent to population doubling times (*T*_d_) of 10 hours, 7 hours, and 3.5 hours, respectively; μ = specific growth rate defined as grams of biomass generated per gram of biomass present per unit time). We then looked for changes that correlated with growth rate under all four nutrient-limiting conditions, using principal components analysis (PCA; see Materials and methods). Trends that appear in all four nutrient-limited series, including carbon-limited cultures with equivalent glucose concentrations, cannot be attributed to variations in residual substrate concentrations (for example, different levels of glucose repression). Instead, they must be due to intrinsic growth-rate-related processes.

Gene expression at the mRNA level was investigated by transcriptome analysis using Affymetrix hybridization arrays. Proteomic studies were performed using isotope tags for multiplexed relative and absolute quantification (iTRAQ) [34,35]. In this case, the four tags and labeling schema applied (see Materials and methods) allowed us to test and compare the proteomes of cells grown at μ = 0.1/hour (*T*_d _= 7 hours) with those of cells grown at μ = 0.2/hour (*T*_d _= 3.5 hours) for all four nutrient limitations. We were able to detect and quantify a significant proportion of the yeast proteome (around 700 proteins per nutrient-limiting condition; 1,358 proteins in total; see Materials and methods). For the metabolome, which is the closest genomic level to the cell's phenotype [36,37], gas chromatography coupled to time-of-flight mass spectrometry (GC/TOF-MS) was used to analyze the complement of intracellular and extracellular metabolites, that is, the endo- and the exometabolomes [38,39].

**Figure 1:**
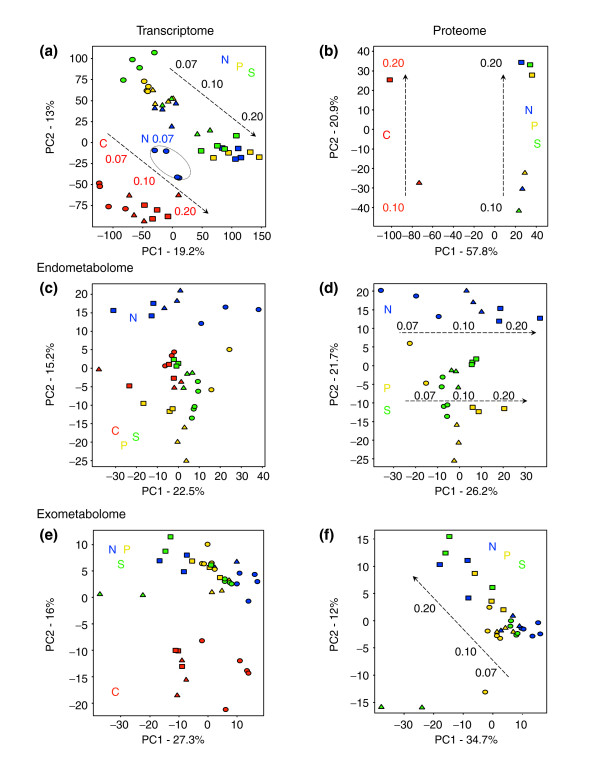
Principal components analyses (PCA) of steady-state chemostat cultures. The *x *and *y *axes represent the two main principal components (PC1, PC2), the groups responsible for the majority of the variance in each global dataset (see Materials and methods). PCA and growth-rate trends (dashed lines) at the **(a) **transcriptome (mRNA) level and **(b) **proteome level. **(c,e) **PCA and trends at the (c) endometabolome and (e) exometabolome level, respectively. **(d,f) **Same as (c) and (e) for carbon-sufficient chemostat series (N-, P- and S- limited series; see text for explanation). Each symbol represents a culture condition, colored as follows: red, carbon (C) limitation; blue, nitrogen (N) limitation; yellow, phosphate (P) limitation; green, sulfate (S) limitation. The symbol shape indicates the specific growth rate, μ, of the culture: ovals, μ = 0.07/h; triangles, μ = 0.1/h; rectangles, μ = 0.2/h. The circle round the blue ovals includes chemostat series exhibiting pseudohyphal growth (see text). As a test of reproducibility, for each nutrient-limiting condition, one of the three μ = 0.07/h exometabolome samples was analyzed in triplicate.

Principal components analyses (PCA) of transcriptome, proteome, and endo- and exometabolome data showed clear growth-rate-associated trends for all omic levels (Figure 1). In the case of the endo- and exometabolomes, these trends are clearly revealed after independent analysis of the carbon-limited and carbon-sufficient datasets (see Figure 1d,f). This is because, in contrast to all other nutrient-limited steady states, the endo- and exometabolomic profiles from cells in glucose-limited steady-state cultures showed no clear growth-rate trend. We infer from this that yeast cells are well-adapted to growth under carbon-limited conditions and are able to adjust the individual fluxes through their metabolic network to regulate overflow metabolism whatever overall flux is imposed by the external supply of carbon substrate. This result is congruent with our data from category 2 experiments (D. Delneri and S.G.O., unpublished work) in which we have examined the effect that reducing the copy number of individual genes in diploid cells has on flux by performing competition experiments, in chemostat cultures, between yeast strains heterozygous for individual gene deletions.

For all three levels of 'omic analysis, the data show a clear distinction between carbon-limited and carbon-sufficient cells (Figure 1). Once the data from the carbon-limited steady states have been excluded, both the endometabolome and the exometabolome data from all three carbon-sufficient cultures show a clear and consistent growth-rate trend (compare Figure 1c,e with 1d,f). In addition, for the endometabolome data, the second principal component separates the ammonium-limited cells from those grown under phosphate and sulfate limitation (Figure 1d).

Figure 1a shows that the transcriptome data from nitrogen-limited cells at the lowest growth rate studied (0.07/hour) do not obey the general growth-rate trend. Uniquely among all the cultures that we analyzed, cells from these cultures had a pseudohyphal, rather than a budding, growth pattern; these data should allow us to define those genes whose expression is specifically associated with filamentous growth. We did not examine the proteome at μ = 0.07/hour and so do not know whether this difference is reflected at the protein level. However, the proteomic data from all steady-state cultures at μ = 0.1/hour and 0.2/hour show the same clear discrimination between carbon-limited and carbon-sufficient cells and the same growth-rate-associated trend as was found with the metabolome and transcriptome data. The fact that all 'omes' studied display a growth-rate-associated trend suggests a multilevel control underlying global regulation of cell growth, and we now examine these levels in some detail.

### Growth-rate control at the transcriptional level

Hybridization-array technology was used to determine how the levels of gene transcripts changed with both flux (growth rate) and nutrient environment. While the transcriptomes of cells grown under each of the four nutrient-limiting conditions have their own characteristics (see Additional data files 1 (Figures S1 and S2), 2 (Tables S1 and S2), and 3), there is a common qualitative and quantitative response to increasing growth rate that is independent of the specific nutrient limitation (see Figure 1a, and Additional data file 1 (Figures S3 and S4)).

We performed an analysis of covariance (ANCOVA) in order to identify those genes whose transcription was significantly and consistently upregulated or downregulated with growth rate in all four nutrient-limitation conditions studied (see Additional data file 1 (Figure S3)). These genes were ranked by estimates of false discovery rate (FDR), in this case the *q*-value [40] of the ANCOVA model (obtained from the *p *value, after multiple testing correction; see Additional data file 4), which represents the relative significance in the (condition-independent) change in gene expression with growth rate. Taking these *q*-values, we applied a cut-off of 5% (*q *= 0.05 [40]; see Materials and methods). This produced a set of 493 genes whose expression is significantly upregulated with increasing growth rate (*q *< 0.05; see also Additional data file 4), and 398 genes that exhibited significant and concomitant downregulation with increasing growth rate, independent of the culture conditions (see Additional data files 1 (Figure S4) and 2 (Tables S3 and S4)).

Essential genes, that is, genes whose deletion results in a failure to grow on rich glucose-containing medium [41,42], are statistically overrepresented in the list of genes significantly upregulated with growth rate (161 out of 493 (32.6%); the fraction of all yeast genes that are essential is around 17%), whereas they are significantly underrepresented in the downregulated list (22 out of 398 (5.5%, again compared to 17%)). The proportion of essential open reading frames (ORFs) in the downregulated set (5.5%) is significantly different from the proportion of essential ORFs that we find not to be subject to growth-rate control (16.8%). In fact the fraction of essential ORFs in this non-growth-regulated set is indistinguishable from the proportion of all yeast ORFs that are essential to growth (16.6%).

**Figure 2:**
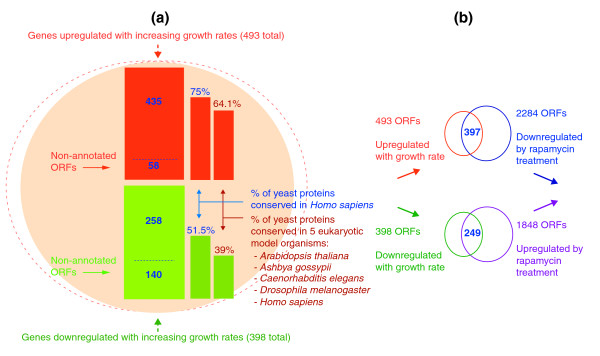
Cell-growth regulation of gene expression at the transcriptional level. **(a) **Groups of genes significantly upregulated (main red block) and downregulated (main green block) with growth rate irrespective of the nutrient-limiting condition, and their conservation in eukaryotes. The smaller blocks to the right represent the percentages of conserved orthologous proteins in *Homo sapiens *alone and in five model eukaryotic organisms [52]. The number of non-annotated open reading frames (ORFs) in up- and downregulated lists (that is, ORFs/genes of unknown function Affymetrix annotation 12 July 2006) is included. **(b) **Target-of-rapamycin (TOR) regulation at the transcriptional level. Genes upregulated and downregulated with growth rate are indicated by red and green circles, respectively. Groups of genes whose transcription is significantly downregulated by rapamycin treatment are indicated by the blue circle; those upregulated by the purple circle. Overlapping areas indicate groups of specific growth-related genes whose expression is significantly affected by rapamycin at the transcriptional level.

Despite the fact that genes that are downregulated with increasing growth rate are rarely essential on rich medium [41,42], the central role of all growth-regulated genes in cell growth is confirmed by independent studies on deletion mutants. This applies to both the essential and the non-essential genes in both the up- and downregulated sets (Figure 2a). Thus, null mutations in many of the genes that we have identified as growth-regulated have been reported to either be lethal or produce a severe growth defect (84.0% in the upregulated set; 64.6% in the downregulated set) [41,42] (see Additional data file 2 (Tables S3 and S4)). In all, our studies have revealed the importance of nonessential genes whose expression is growth-rate regulated in determining whether yeast can grow at normal rates. This applies to genes whose expression is downregulated with increasing growth rate, as well as those under positive growth-rate regulation.

From all these studies, a significant number of genes (891; 15% of the protein-encoding genes in the genome) have their transcript levels determined by growth rate (Figure 2a). While many of these genes (198, 22.2%) correspond to ORFs of so far unknown function (Figure 2a; see also Additional data file 2 (Tables S3 and S4)), according to Affymetrix (12 July 2006) and Gene Ontology (GO) annotations [43], an examination of the functions determined by the remainder is instructive. Using two different GO analysis tools (GoMiner [44] and GenMAPP [45]; see Additional data files 1 (Figures S5-S16) and 2 (Tables S5-S11)) we showed that the 435 genes of known function that are upregulated with growth rate (see Figure 2a and Additional data files 1 (Figure S4) and 2 (Table S3)) include a significant proportion whose products are involved in the biological processes of translation initiation, ribosome biogenesis and assembly, protein biosynthesis, RNA metabolism, nucleobase, nucleoside, nucleotide and nucleic acid metabolism, nucleus import and export and proteasome function (see Additional data files 1 (Figures S5 and S11) and 2 (Tables S3 and S5)). The corresponding analysis of GO molecular functions for the same gene set showed the following to be overrepresented: translation initiation factor activity and nucleic acid (RNA) binding, structural constituent of ribosome activity, ligase activity forming aminoacyl-tRNAs and DNA-directed RNA polymerase activity (see Additional data files 1 (Figures S6 and S12) and 2 (Table S6)). At the level of cellular components, GO studies indicated that the most representative upregulated processes occur in a variety of subcellular compartments (cytosol, exosome, and nucleus) and complexes (for example, eukaryotic translation initiation complexes, nucleolus, ribosome subunits, and the proteasome core complex; see Additional data files 1 (Figures S7 and S13) and 2 (Table S7)). For a comprehensive analysis of processes upregulated with increasing growth rate, see Additional data file 5.

GO analysis of the set of 258 genes of known function whose transcription was significantly downregulated with increasing growth rate (see Figure 2a and Additional data files 1 (Figure S3) and 2 (Table S4)) shows that a high proportion of these genes correspond to the following biological processes: response to external stimulus, cell communication and signal transduction, autophagy, homeostasis, response to stress, vesicle recycling within Golgi (see Additional data files 1 (Figures S8 and S14) and 2 (Table S9)). The most overrepresented GO molecular function categories for this gene set correspond to a variety of catalytic, signal transduction, transcription regulator, and transport activities. These include receptor signaling protein activity, protein kinases, phosphotransferase, oxidoreductase and ATPase activity coupled to transmembrane movement of ions, and phosphorylation mechanisms (see Additional data files 1 (Figures S9 and S15) and 2 (Table S10)). At the level of cellular component, downregulated processes occur at the level of the plasma membrane, the vacuole, and the repairosome (see Additional data files 1 (Figures S10 and S16) and 2 (Table S11)). Although essential genes are under-represented in this list (22 out of 398; see Additional data file 2 (Table S4) and the *Saccharomyces *Genome Database [42]), the fact that 64.6% of the downregulated genes have been reported to result in growth defects or inviability in gene deletion studies (see Additional data file 2 (Table S4) and [42]) points to a crucial role of these genes in growth-related processes that has yet to be elucidated. All of the 22 essential genes in this set are of known function, but only 11 of them have been reported previously as being directly related to cell growth and maintenance. For a comprehensive analysis of the role of most relevant downregulated processes regulating cell growth at the transcriptional level, see Additional data file 5.

Genes that are downregulated with increasing growth rate are probably involved in maximizing the efficient utilization of cellular resources at each different growth rate and culture condition, particularly when nutrients are scarce. Our data indicate that this is a poorly understood aspect of the cell's economy since a significant number of these genes (140/398; 35.2%) are of as-yet-undetermined function. This is despite the fact that nutrient scarcity is likely to be a common circumstance in the organism's natural environment [46]. Among the genes of known function that are upregulated at low growth rates are those involved in mobilization and storage of available resources at the level of the vacuole (see Additional data file 1 (Figure S20)). Another interesting example of genes that are upregulated at low growth rates are those involved in autophagy (see Additional data file 1 (Figure S21)). Autophagy is a major system of bulk degradation of cellular components. It participates in the coordinate degradation of cytoplasmic components, including proteins, large complexes and organelles whose turnover is important in the control of cell growth. Autophagy mediates the shrinkage of the ribosome pool, thus slowing cell growth when nutrients are limiting [47].

Autophagy in yeast has been reported to be a TOR-mediated response to nutrient starvation [48], and we have demonstrated previously the induction of autophagy genes in stationary phase [19]. Autophagy genes are well conserved from yeast to mammals, suggesting that it is a fundamental activity of eukaryotic cells, being implicated in processes such as homeostasis, development and differentiation [47]. Other genes that are upregulated at low growth rates are those encoding specific transcriptional repressors whose action results in the activation of alternative routes for the assimilation of substrates and/or as an adaptation to the environment.

In all, the data on the downregulated genes present a picture of the yeast cell at low growth rates activating pathways involved in the response to external stimuli, maintenance of homeostasis, vacuolar transport and storage, and autophagy; the whole being directed towards a more efficient use of scarce resources. Finally, we have found that genes that were annotated previously as being involved in 'response to stress' [42,49,50] are upregulated at low growth rates. Moreover, we have confirmed these findings at the proteome level (see proteomic studies (Table 1)). This demonstrates that a large part of what others have termed the 'generalized stress response' may more properly be viewed as a slow-growth response.

**Table 1:** Groups of relevant biological processes regulated at the protein-expression level

Biological process	Proteins (examples)	*p*-value (GO studies)
**Upregulated**		
Cellular biosynthesis (81)	Ser3p, Cpa1p, Lia1p Rpl10p	1.1E-29
Amino-acid and derivative metabolism (33)	Gln1p, Leu1p, Lys4p	2.6E-21
Translation (42)	Rpl6ap, Mes1p, Sui3p, Fun12p	4.8E-13
Macromolecule biosynthesis (48)	Mdh2p, Rpl3p, Mes1p, Ths1p	5.7E-12
tRNA aminoacylation (9)	Mes1p, Cdc60p, Ded81p	9.7E-9
Ribosome biogenesis and assembly (22)	Nug1p, Rpl30p, Nop58p, Yf3p	3.2E-7
Purine nucleotide metabolism (8)	Ade17p, Ade1p, Hpt1p	3.8E-6
Sulfur metabolism (8)	Ecm17p, Met10p, Trx2p, Sam2p	2.2E-5
Organic-acid biosynthesis (4)	Ald6p, Fas1p, Fas2p	3.6E-4
Regulation of protein metabolism (6)	Asc1p, Cap2p, Rpl30p	1.2E-3
rRNA processing (8)	Nug1p, Has1p, Utp10p	1.8E-2
		
**Downregulated**		
Cellular carbohydrate metabolism (19)	Gre2p, Tsl1p, Tps1p, Eno1p	2E-7
Coenzyme metabolism (14)	Pan5p, Mdh3p, Npt1p	5.8E-7
Response to stress (27)	Pol30p, Hsp104p, Rvs161p	9.8E-7
Response to stimulus (31)	Lap3p, Akr1p, Ycf1p, Fet3p	1.1E-5
Cellular macromolecule catabolism (17)	Skp1p, Pre3p, Rpn3p, Kar2p	2.3E-4
Vacuole organization and biogenesis (6)	Sec17p, Tpm1p, Vtc2p, Vtc3p	3.6E-4
Transport (35)	Pet9p, Sar1p, Fet3p, Hxt3p	1.1E-3
Cellular lipid metabolism (11)	Ncp1p, Erg1p, Erg27p. Lem3p	7.7E-3
Homeostasis (7)	Skp1p, Ahp1p, Vma5p, Zrt3p	2E-2

Groups of relevant biological processes regulated at the protein expression level from growth rates 0.1 to 0.2/h under carbon limitation are shown here. Proteins significantly upregulated or downregulated with increasing growth rate (relative fold-changes greater than 20%, 141 proteins) from iTRAQ studies were analyzed by GO studies (GO tool, SGD Term Finder [42]). Numbers of proteins obtained per biological process are included in brackets. Full lists of results, including genes significantly regulated at the protein expression level, for each biological process are provided in Additional data file 2 (Tables S19 and S20).

### Cell-growth-related genes subjected to transcriptional control encode a core protein machinery conserved among all eukaryotes

A high percentage of the proteins encoded by the up- and downregulated genes are highly conserved in a variety of 'model' eukaryotes (*Ashbya gossypii*, *Caenorhabditis elegans*, *Arabidopsis thaliana*, *Drosophila melanogaster *and *Homo sapiens*) [51,52], which points to the existence of an essentially conserved 'core' protein machinery governing cell growth in the Eukarya. Thus, 75% of the protein products of yeast genes upregulated with growth rate have orthologs in humans, whereas 52% of the downregulated set have human orthologs (which is not significantly different to the figure of 48% for all *S. cerevisiae *proteins [51]; see Figure 2a and Additional data file 2 (Tables S3 and S4)). Many of these proteins are built into complex machines [53]. Proteins encoded by the upregulated genes participate in a large number of interactions with each other (876 interactions as compared with 287 expected by chance), whereas those encoded by the downregulated genes rarely interact with one another (89 compared with the 193 expected by chance; see Additional data files 2 (Tables S12 and S13) and 4).

### TOR control of cell growth at the transcriptional level

The TOR signal transduction pathway is a central controller of the eukaryotic cell, sensing cellular environment and linking nutrient assimilation with translation initiation and ribosomal protein synthesis to control cell growth [3,4,33,54-56]. Many genes responsible for central growth processes (for example, translation initiation, ribosome biogenesis, autophagy, stability of biosynthetic components) are regulated at the transcriptional level (see Additional data file 2 (Tables S3 and S4)) and are under the direct or indirect control of TOR [32,33] (see Additional data file 1 (Figure S22)). The exact mechanisms by which the TOR pathway controls these processes are not known, but appear to be mediated (at least, in part) by GATA-type, zinc-finger and forkhead transcription factors [32,33,57-60]. We decided to test the generality of the hypothesis that TOR, more specifically the TOR signaling branch that mediates temporal control of cell growth (TORC1) complex [32], is the major regulator of yeast gene expression in response to nutrient availability, and hence of growth rate [3,31-33]. To do this, we examined the impact of rapamycin, a specific inhibitor of the TORC1 complex [32], and widely used to elicit the TOR control response [32,61], on the yeast transcriptome [14].

The results of this examination should be approached with caution for two reasons. First, few inhibitors are completely specific in their action and thus our analysis is likely to be complicated by side-effects of rapamycin on processes other than TOR action. Second, as the addition of the inhibitor would necessarily disturb the steady state of a chemostat culture, we performed this experiment in batch. We have shown previously that the use of batch culture introduces a number of confounding variables to transcriptome analyses that are avoided by the use of chemostats [14,19]. Thus, it may be predicted that the rapamycin-inhibition experiment would show more genes affected than were found to be subject to growth-rate control in our chemostat studies. This, indeed, proved to be the case (Figure 2b). Remarkably, the rapamycin and growth-rate data showed more than 70% of growth-rate-regulated genes to be members of the TOR-responsive sets. We found 397 growth-rate upregulated genes to be downregulated by rapamycin, and 249 genes downregulated by growth rate were upregulated in response to the drug. Thus, 646 growth-rate-regulated genes (72.5%) appear to be specifically controlled by TOR (Figure 2b; see also Additional data files 1 (Figure S23) and 2 (Tables S15 and S16)). Our studies are also in good agreement with previous transcriptional studies on the effect of rapamycin on yeast cultures, showing a characteristic global response, with translational initiation, aminoacyl-tRNA synthetases, RNA polymerases, ribosome biogenesis and proteasome subunits among the most significantly affected biological processes (see Figure 2b and Additional data file 2 (Tables S15-S17) and [61,62]). These are key processes in which our sets of growth-rate-regulated genes are involved.

In our results, none of the genes specifying the components of the TORC1 complex [32,63] appears significantly regulated at the level of transcription (see Additional data file 1 (Figures S24 and S25)), in agreement with previously reported studies (SGD; ORF expression connection studies [42]). Evidence is accumulating that post-transcriptional mechanisms play an important role in the global regulation of cell growth [24,64,65] (see also the section on translational control, below). As an example, many genes reported to be involved in control of cell size or coordination between cell growth and division [9] do not appear regulated at the transcriptional level (see Additional data files 2 (Tables S3 and S4) and 5), showing that it is important to extend these studies to the proteomic level.

### Proteomic signatures of growth-rate change

**Figure 3:**
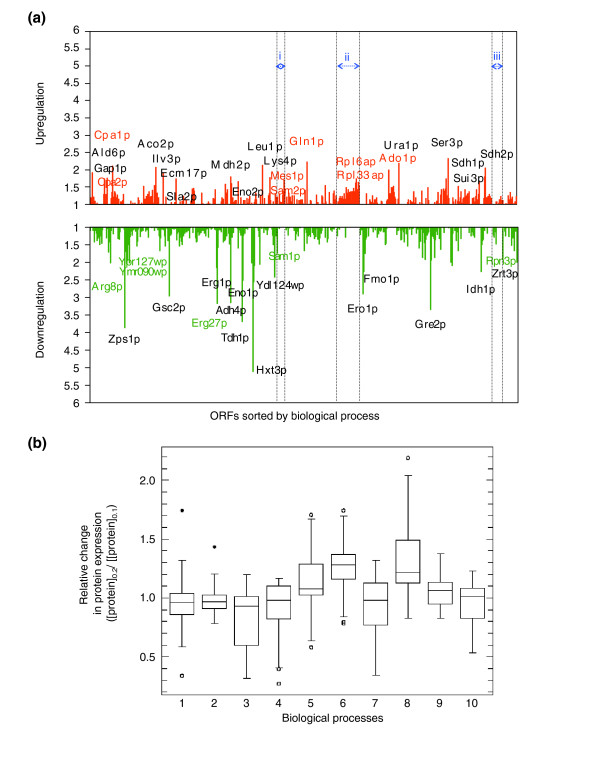
Gene-expression signatures at the protein level. **(a) **The graph shows the pattern of relative changes (fold change) in protein levels with a shift in growth rate (μ) from 0.1 to 0.2/h (doubling time, *T*_d _= 6.9 to 3.5 h) under conditions of carbon limitation (663 proteins in total). ORFs were sorted by biological process [42]. i, Methionine biosynthesis; ii, protein biosynthesis; iii, ubiquitin-dependent protein catabolism. Red, upregulated protein expression; green, downregulated. Selected groups of proteins whose levels are consistently upregulated or downregulated with growth independently of culture condition are labeled in the appropriate color. **(b) **Box-plot of relative changes in protein expression from growth rate 0.1 to 0.2/h of proteins of representative biological processes (>10 proteins identified per process). 1, Cell wall organization and biogenesis; 2, endoplasmic reticulum (ER) to Golgi transport; 3, ergosterol biosynthesis; 4, glycolysis; 5, methionine biosynthesis and methionine metabolism; 6, protein biosynthesis; 7, protein folding; 8, purine nucleotide, purine base and pyrimidine base biosynthesis; 9, regulation of transcription; 10, ubiquitin-dependent protein catabolism. Open and solid dots indicate presence of outliers that lie more than 3 or 1.5 times the interquartile range, respectively.

Most global gene-expression studies have been entirely at the transcriptome level and often assume that changes in transcript levels should correlate with changes at the protein level. However, there is ample evidence that this is a dangerous assumption [21-24,65-69]. We extended our study to the proteome level using iTRAQ [34,35], covering a significant proportion of the yeast proteome (around 700 proteins per nutrient-limiting condition; 1,358 in total; see Materials and methods). For example, we examined the differences in protein levels (proteomic signatures) between cells growing at μ = 0.1/hour and those growing at 0.2/hour under carbon limitation (Figure 3 and Additional data file 2 (Table S18)), and found a number of proteins and biological processes to be significantly up- and downregulated under these conditions (Table 1 and Additional data file 2 (Tables S19 and S20)). Remarkably, as with the transcriptome profiles, these proteomic signatures appear to be characteristic for each nutrient-limiting condition, but there is also a common pattern that represents the proteomic response to a growth-rate shift from μ = 0.1 to 0.2/hour (see Figure 3a and Additional data files 1 (Figure S26) and 2 (Tables S18 and S21)). Relative changes in proteome levels of proteins participating in relevant biological processes are shown in Figure 3b. Again, in common with the transcriptome data, most of the changes in protein levels lie in a range between a less than twofold decrease and a less than twofold increase (Figure 3a and Additional data file 1 (Figure S26)). Similar analyses (that is, ANOVA) to those performed on the transcriptome data can be applied to identify groups of proteins that are consistently and significantly up- or downregulated with growth rate (see Additional data file 4).

Among the groups of proteins whose levels appear consistently up- or downregulated with growth irrespective of the specific nutrient limitation (see Figure 3 and Additional data files 1 (Figure S26) and 2 (Tables S22 and S23)) are proteins of the translational machinery (for example, translation initiation and elongation factors, ribosomal proteins, aminoacyl-tRNA synthetases), enzymes involved in methionine and methyl cycle metabolism, and regulatory enzymes of amino-acid and other relevant biosynthetic pathways. Selected groups of proteins are marked in color in Figure 3a. As a relevant example, proteomic studies reveal different responses in the levels of the two *S*-adenosylmethionine synthetases, Sam1p and Sam2p (see Figure 3a and Additional data file 1 (Figure S26)). This, and the fact that the *SAM2 *gene was significantly upregulated at the transcriptional level (Additional data file 2 (Table S3)), are in accordance with previous reports [70].

Finally, nutrient-independent changes in levels of metabolic enzymes (see Figure 3a; the most relevant are included in Additional data file 2 (Table S24)) with growth rate will be of particular importance for the elucidation of the yeast cell's strategies for the control of central metabolic fluxes during cell growth, and for the identification of groups of metabolic enzymes consistently up- and downregulated at the protein level (for example, amino-acid biosynthetic enzymes; Table 2). These studies have direct implications for the design of new comprehensive metabolic engineering strategies, and will be discussed in the section below on metabolic control, where (for example) the role of the Sam1p and Sam2p isoenzymes is considered.

**Table 2:** Amino-acid biosynthetic enzymes with protein levels consistently up- and downregulated with growth rate under all nutrient-limiting conditions

	Enzymes	
	
Amino-acid biosynthetic pathway	Upregulated	Downregulated
Arginine	Aco1p, Aco2p, Cpa1p, Cpa2p	Arg1p, Arg8p
Homocysteine, cysteine, methionine, and sulfur compounds	Ecm17p, Met10p, Met13p, Sam2p, Met6p, Ado1p	Sam1p
Glutamine	Gln1p	
Leucine, isoleucine, valine	Ilv3p, Leu1p	
Lysine	Aco1p, Aco2p, Lys2p, Lys4p	

Aco1p, aconitase; Aco2p, putative aconitase isozyme; Ado1p; adenosine kinase; Arg1p, arginosuccinate synthetase; Arg8p, acetylornithine aminotransferase; Cpa1p, small subunit of carbamoyl phosphate synthetase; Cpa2p, large subunit of carbamoyl phosphate synthetase; Ecm17p, sulfite reductase beta subunit; Gln1p, glutamine synthetase; Ilv3p, dihydroxyacid dehydratase; Leu1p, isopropylmalate isomerase; Lys2p, alpha aminoadipate reductase; Lys4p, homoaconitase; Met6p, methionine synthase; Met10p, sulfite reductase alpha subunit; Met13p, methylenetetrahydrofolate reductase isozyme; Sam1p, *S*-adenosylmethionine synthetase isozyme; Sam2p, *S*-adenosylmethionine synthetase isozyme.

### Proteome-transcriptome correlations

**Figure 4:**
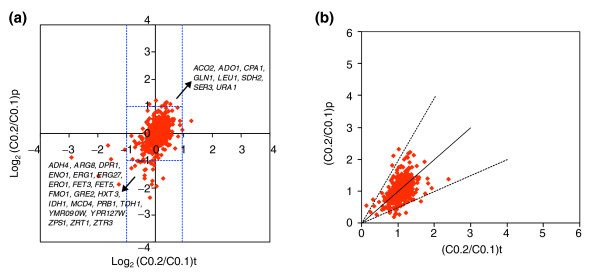
Integration of proteome and transcriptome studies. Proteome-transcriptome correlations are determined by the relative changes in protein levels versus relative changes in transcriptional levels from μ = 0.1 to 0.2/h under conditions of carbon limitation. **(a) **Log_2 _correlations with the most relevant outliers (cases in which changes in transcript levels do not result in comparable changes at the protein level) named. **(b) **Correlations between relative changes in natural values. The lines with *y*/*x *slope 0.5, 1 and 2 respectively allow to delimit groups of protein/transcript pairs that are correlated (*y*/*x *ratio >1) and anti-correlated (*y*/*x *ratio <1), and their limits (majority of them with *y*/*x *ratios within 0.5 and 2; [0.5 <*y*/*x *ratio < 2]).

**Figure 5:**
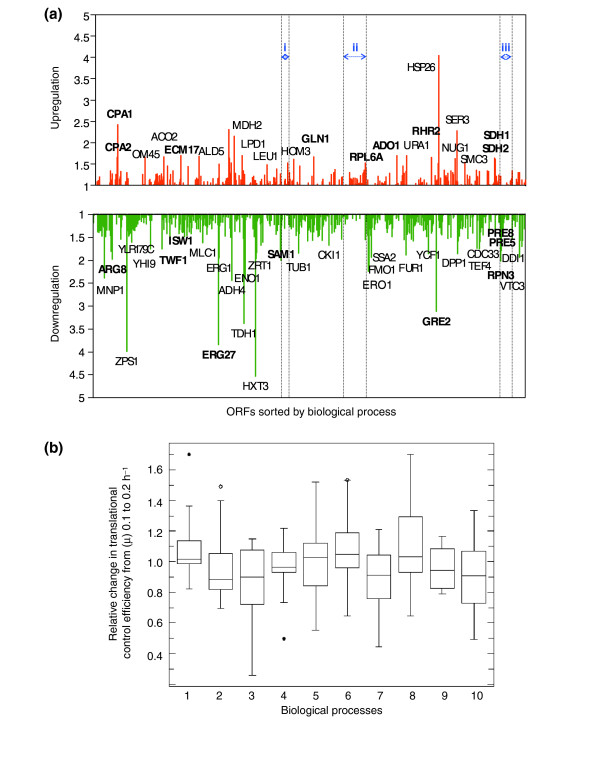
Cell-growth regulation of gene expression at the translational level. Translational control. **(a) **Patterns of relative changes in translational control efficiencies from growth rate (μ) 0.1 to 0.2/h, under conditions of carbon-limitation. ORFs sorted by biological process [42]. i, Methionine biosynthesis; ii, protein biosynthesis; iii, ubiquitin-dependent protein catabolism. Selected groups of transcripts whose translational control efficiency is consistently up- or downregulated with growth independently of culture condition are marked in bold. Red, upregulation; green, downregulation. **(b) **Box-plot of relative changes in translational control efficiencies from growth rate (μ) 0.1 to 0.2/h of transcripts in representative biological processes (>10 proteins identified per process). 1, Cell wall organization and biogenesis; 2, ER to Golgi transport; 3, ergosterol biosynthesis; 4, glycolysis; 5, methionine biosynthesis and methionine metabolism; 6, protein biosynthesis; 7, protein folding; 8, purine nucleotide, purine base and pyrimidine base biosynthesis; 9, regulation of transcription; 10, ubiquitin-dependent protein catabolism. Open and solid dots indicate presence of outliers that lie more than 3 or 1.5 times the interquartile (IQR) range, respectively.

Because our transcriptome and proteome data had been obtained from the same samples of cells from chemostat cultures in steady state at growth rates of both 0.1 and 0.2/hour, and as these data had been normalized and statistically analyzed in the same way, we were able to make a realistic determination of the congruence between the level of any gene transcript and its cognate protein product(s). Example results are presented in Figure 4 for the glucose-limited steady states. Overall, the correlation coefficients (r) for each nutrient-limiting condition (C, N, P and S limitation) lie between 0.4 and 0.7, indicating only a moderate global congruence between transcript and protein levels (see Additional data file 6), in agreement with some previous studies [65-69,71]. The fact that mRNA changes do not generally correlate with protein changes suggests a widespread role for post-transcriptional mechanisms in the control of yeast's growth rate (see below). Most transcripts show a relative change in their level, between both growth rates of 0.1/hour and 0.2/hour, that is within a twofold range up and down, and the same is true for their cognate proteins. However, there are a number of transcript-protein pairs that are significant outliers, cases in which changes in transcript levels do not result in comparable changes at the protein level (for example, *ADH4*/Adh4p and *ADO1*/Ado1p in Figure 4); examples of these outliers are shown more clearly in Figure 5, and are discussed in the following section.

### Growth-rate-associated changes in translational control efficiencies

A number of post-transcriptional mechanisms might be involved in modulating the cellular concentration of a given protein relative to that of the mRNA species that encodes it. These include mRNA recruitment from the nucleus and p-bodies, polyadenylation states, level of polysomal occupancy per transcript, and rates of protein degradation [21-24,72-74]. To encompass all of these mechanisms of translational control and quantify their overall effect, we define the effective 'translational control efficiency' (Trlc Eff_i_) of a given messenger RNA in terms of its P/R ratio [protein_i_]/[mRNA_i_] (see Materials and methods and Additional data file 7), and show that the ratio of relative change in the level of a protein to the relative change in its cognate mRNA (obtainable from proteome-transcriptome studies; see above) is equal numerically to the ratio of relative changes in translational control efficiencies between the two conditions studied (see Materials and methods and Additional data file 7).

By this means, and on a genome-wide scale, we can quantify the relative changes in the overall translational control efficiencies of mRNA molecules corresponding to a shift from μ = 0.1 to 0.2/hour (that is, a doubling in specific growth rate). The results are presented in Figure 5 (for just the carbon-limited steady state) and in Additional data file 8. The pattern of changes suggests that the translational control efficiencies of particular mRNAs are modulated selectively in order to fine-tune protein activities and metabolic fluxes of relevant biological processes during cell growth (Figure 5a,b). The pattern of changes in translational control efficiencies is dependent on the specific nutrient-limiting condition, with most transcripts showing a less than twofold change (up or down) in their translational efficiencies, but a few undergo much larger relative changes (Figure 5a, see also Additional data files 1 (Figure S27) and 8).

This metric of the relative change in translational control efficiency allowed us to make a quantitative estimate of the relative contribution of post-transcriptional control mechanisms to a change in growth rate. For each nutrient-limiting condition, more than 35% of all transcripts were found to change their translational efficiency to a significant (greater than 20%) extent. Further studies, including analysis of post-translational modifications across the proteome (for example, phosphorylation and glycosylation), will provide a more complete picture of the role of post-transcriptional control during cell growth.

From all these data, we were able to extract groups of transcripts whose translational control efficiencies are consistently up- or downregulated with growth rate, irrespective of the limiting nutrient. Transcripts in this category include those encoding components of the translational machinery, enzymes subject to covalent or allosteric regulation that are involved in amino acid and other biosynthetic pathways, and regulatory proteasome subunits. Selected cases are marked in bold in Figure 5a and summarized in Additional data file 2 (Table S25). As an interesting example, the relative level of the transcript of *CPA1 *(encoding the small subunit of the multimeric enzyme carbamoyl phosphate synthetase (CPSase) in the arginine biosynthetic pathway) does not change with growth rate (see Additional data file 2 (Table S3)), but the overall efficiency with which this mRNA is translated goes up significantly with growth rate (see Figure 5a, and Additional data file 8). Although CPSase activity has been found to be subject to regulation at the transcriptional, translational and metabolic levels [75-78], under the specific conditions tested (synthetic medium under nutrient-limited conditions, with ammonium as sole nitrogen source), it appears to be regulated mainly at the translational level.

### Growth-rate control at the level of the metabolome

How are the metabolic fluxes characteristic of an increase in the rate of biomass accumulation actually controlled? To what extent are these fluxes regulated by gene expression (enzyme expression levels) or by metabolic regulation? To answer these questions, the quantitative proteomic data must be integrated with those on the metabolome. This is, without doubt, the most difficult challenge in data integration that exists in functional genomics or systems biology. To a large extent, it is because the metabolome, in contrast to the transcriptome and the proteome, has no simple, direct connection to the genome [79]. We have recently developed statistical approaches with which to integrate transcriptome data with those for a small number of key metabolites (for example, glucose, ethanol, CO_2_) [80], but we have yet to extend this to the entire metabolome. This is a field in which many different strategies are likely to be required and, indeed, are starting to be developed - for instance, metabolic network topology [81].

**Figure 6:**
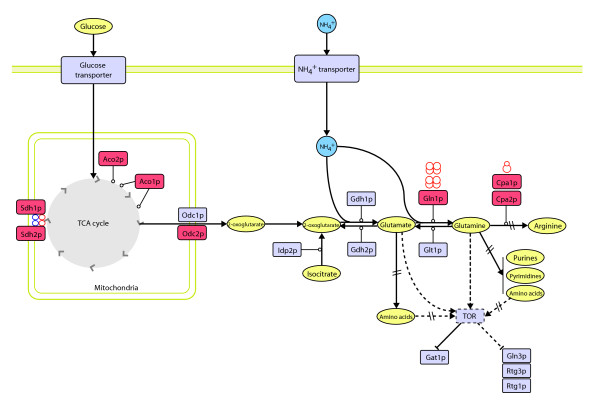
Integration of proteome and metabolic control to show regulation of carbon and nitrogen metabolic fluxes at the protein (enzyme) level. Shown here are the coupling of carbon and nitrogen fluxes at the level of glutamate dehydrogenase (Gdh1p, Gdh2p) and glutamine synthetase (Gln1p), the regulation of arginine biosynthesis at the carbamoyl phosphate synthetase (Cpa1p, Cpa2p) level and amino-acid biosynthesis, and amino-acid sensing by TOR. Selected proteins with levels consistently upregulated (red) with growth independently of culture conditions are shown. Enzymes responsible for the cytosolic 2-oxoglutarate pool: Aco1p and Aco2p, aconitase and putative aconitase isoenzyme; Odc1p and Odc2p, mitochondrial 2-oxoglutarate transporters; Idp2p, NADP-specific isocitrate dehydrogenase. Enzyme subunits coupling the oxidation of succinate to the transfer of electrons to ubiquinone: Sdh1p and Sdh2p, succinate dehydrogenase, flavoprotein, and iron-sulfur protein subunits, respectively. Metabolic diagram from [42, 91, 92] and drawn using Cell Designer [136] and Adobe Illustrator [137].

**Figure 7:**
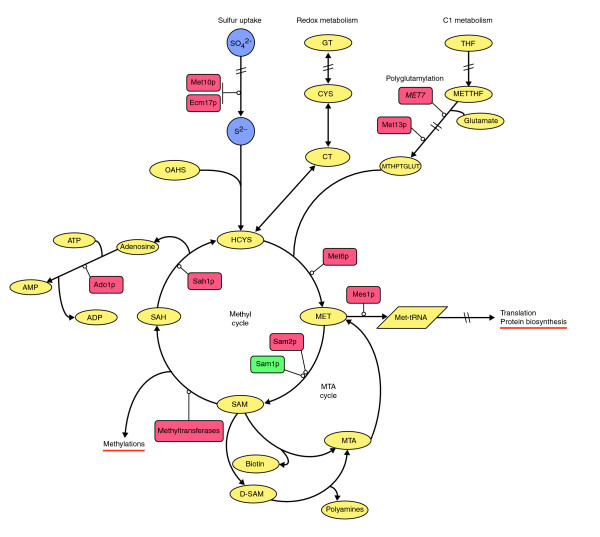
Integration of proteome and metabolic control to show regulation of sulfur and C1 (folate) metabolic fluxes at the protein (enzyme) level. Selected proteins with levels consistently upregulated (red) or downregulated (green) with growth independently of culture conditions are shown. Sulfur, C1 metabolism, methyl cycle, methionine and *S*-adenosylmethionine (SAM) fluxes towards methylation of proteins, rRNAs and tRNAs, and protein biosynthesis are shown here. Metabolic pathways and enzymes are from [42,82, 103-105] and the diagram is drawn with Cell Designer [136] and Adobe Illustrator [137]. Reverse methionine biosynthetic pathways [83] have been omitted for clarity. Metabolite abbreviations: THF, tetrahydrofolate; METTHF, 5,10-methylenetetrahydrofolate; MTHPTGLUT, 5-methyltetrahydropteroyltriglutamate (donor of the terminal methyl group in methionine biosynthesis); GT, glutathione; CYS, cysteine; CT, cystathionine; OAHS, *O*-acetylhomoserine; HCYS, homocysteine; MET, methionine; SAM, *S*-adenosylmethionine; SAH, *S*-adenosylhomocysteine; D-SAM, decarboxylated *S*-adenosylmethionine; MTA, methylthioadenosine. Metabolic steps (genes/enzymes): Met10p, sulfite reductase alpha subunit; Ecm17p, sulfite reductase beta subunit; *MET7*, folylpolyglutamate synthetase (Met7p not detected; the relevance of polyglutamylation in the C1 metabolism branch was demonstrated at the transcriptional level (see text)); Met13p, methylenetetrahydrofolate reductase isozyme; Met6p, methionine synthase; Mes1p, methionyl-tRNA synthetase; Sam1p, S-adenosylmethionine synthetase isozyme; Sam2p, S-adenosylmethionine synthetase isozyme. Sah1p, S-adenosyl-L-homocysteine hydrolase; Ado1p, adenosine kinase.

In the current study, we used the ANOVA analysis applied to the iTRAQ proteomic data to identify proteins whose levels were consistently up- or downregulated with growth rate (see Figure 3 and Additional data file 2 (Tables S22-S24)). This analysis highlighted two growth-related metabolic processes: the coupling of carbon and nitrogen fluxes towards the synthesis of amino acids, for example, glutamine and arginine (Figure 6); and the flux of methionine and *S*-adenosylmethionine (SAM through the methyl cycle [82,83] (Figure 7).

#### Coupling of carbon and nitrogen fluxes towards amino-acid biosynthesis

In a synthetic medium with ammonium as sole nitrogen source, the cell must synthesize all its amino acids *de novo*. This implies an efficient coupling of carbon and nitrogen fluxes from 2-oxoglutarate, increasing metabolic fluxes through glutamate dehydrogenase and glutamine synthetase towards the synthesis of all necessary amino acids (Figure 6) [84]. 2-Oxoglutarate, considered to be one of the 12 basic precursor metabolites [85], is primarily synthesized in the mitochondrion through the tricarboxylic acid cycle (TCA). In our studies, we found Aco1p (aconitase) and Aco2p (a putative aconitase isoenzyme with 55% aminoacid sequence identity to Aco1p [86]) to be the TCA cycle enzymes that were most significantly upregulated at the level of protein expression (see Figure 6 and Additional data file 2 (Table S22)). This points to an increase in flux towards *cis*-aconitate and isocitrate (note that Aco1p participates in two consecutive steps in the TCA cycle). At the same time, our endometabolome studies showed that the steady-state levels of citrate, the initial substrate for aconitase, fell with increasing growth rate (see Additional data file 2 (Table S26)).

Significant upregulation at the level of protein expression towards increasing TCA fluxes was also found at the level of succinate dehydrogenase, the enzyme complex coupling oxidation of succinate to the transfer of electrons to ubiquinone. Both Sdh1p and Sdh2p (the flavoprotein and iron-sulfur subunits of the succinate dehydrogenase complex) were significantly upregulated with growth rate (*q *= 0.051 and 0.046, respectively). Once again, metabolome studies showed a decrease in the *in vivo *steady-state levels of the corresponding substrate, succinate, at higher growth rates (see Additional data file 2 (Table S26)).

Among the enzymes responsible for the supply of 2-oxoglutarate in the cytosol, Idp2p (NADP-isocitrate dehydrogenase) and Odc1p (one of two isoforms of the mitochondrial 2-oxoglutarate transporter [87]) were not detected in our proteomic analyses and the transcriptional patterns of their cognate genes were not in the growth-rate-regulated set (*q *for *IDP2 *= 0.13; *q *for *ODC1 *= 0.32; no clear trends with growth rate). However, *ODC2*, which encodes the other isoform of the mitochondrial transporter, Odc2p, is consistently and significantly upregulated with growth rate at both the mRNA (*q *= 0.05) and protein (*q *= 0.12) levels. This demonstrates the importance of mitochondrial transport in the regulation of amino acid biosynthesis and represents a first example of the differential regulation of two enzyme isoforms (with 61% amino-acid sequence identity) with growth rate (see below).

In addition to increased levels of Odc2p, our proteomic data also demonstrate that the levels of glutamine synthetase (Gln1p) as well as the small and large carbamoyl-phosphate synthase subunits (Cpa1p and Cpa2p) are upregulated with growth rate (see Figure 3a and Additional data files 1 (Figure S26) and 2 (Table S27)). These are important regulatory enzymes whose expression and activity have been reported to be tightly regulated at the transcriptional, translational, post-translational and metabolic levels [75-78,84,88-90]. The endometabolome data showed no significant growth-rate-associated change in the steady-state 2-oxoglutarate and glutamine levels, with only glutamate exhibiting a decrease in its intracellular level (see Additional data file 2 (Table S26)). Glutamate is one of several metabolites sensed by TOR, which regulates the activity and localization of the Gat1p (Nil1p) transcription factor, which (in turn) mediates nitrogen catabolite repression in response to intracellular glutamate (see Figure 6) [91,92].

#### Metabolic fluxes towards methionine and S-adenosylmethionine

*S*-adenosylmethionine (AdoMet or SAM), the methyl donor for the majority of methyltransferase reactions [93,94] is one of the most connected metabolites in the cell, after ATP. It participates in a myriad of biochemical processes in different subcellular compartments (for example, cytosol, nucleus, and mitochondria) [82,83,95]. How is the synthesis of this central metabolite regulated and its internal fluxes appropriately distributed?

At the metabolic level, yeast cells have been reported to contain at least two separate SAM pools, with different turnover rates, a labile cytosolic pool and a more stable organellar (mainly vacuolar) pool [96]. We detected SAM (together with low levels of cystathionine, cysteine and glutathione - this last confirming negligible oxidative stress) in our steady-state endometabolome samples (see Additional data file 2 (Table S28)), but we cannot determine how it is partitioned between the organellar and cytosolic compartments. Nonetheless, our results show that gross SAM levels do change with growth rate in a manner that is specific for each of the different nutrient limitations examined.

*Saccharomyces cerevisiae *contains two *S*-adenosylmethionine synthetase genes, responsible for the synthesis of SAM from methionine, *SAM1 *and *SAM2*. The protein products of these two genes are 92% identical [70,82]. Sam1p is the most abundant isoenzyme and is localized in the cytoplasm [97,98]. It is a highly interconnected protein and interacts with proteins involved in a number of central metabolic processes (for example, multimeric enzymes in glycolysis/gluconeogenesis and amino acid biosynthetic pathways; Pfk1p, Pfk2p, Gpm1p, His4p, Trp2p, Trp3p, and proteasome subunits; Rpn1p, Rpn2p), nuclear pore proteins (for example, Kap95p, Kap104p, Kap123p, Crm1p, Mtr10p, Nup2p), mitochondrial proteins (for example, Mpm1p, Mis1p, Mdj1p), vacuolar proteins and proteins involved in vacuolar protein sorting (for example, Vps13p, Vps1p, Vth2p, Vma6p). These interactions have been extracted from the BioGRID database of curated interactions [99] and the paper by Gavin and coworkers [100]. On the other hand, Sam2p has no clear subcellular localization [97,98] and is rarely associated with other proteins [99,100]. No specific functions have so far been assigned to these two isoenzymes in *S. cerevisiae *[82].

We find that *SAM2 *mRNA levels are significantly upregulated with growth rate (see Additional data file 2 (Table S3)), whereas the *SAM1 *transcript shows no significant upregulation with increasing growth rate, confirmed by quantitative real-time PCR (QRT-PCR). More pertinently, our proteomic studies under all four nutrient-limiting conditions show that the increase in growth rate from μ = 0.1 to 0.2/hour entails an increase in the levels of a number of enzymes (for example, Ado1p, Met13p, Met6p, Sam2p) involved in methionine and SAM biosynthesis, and these results have been confirmed by two-dimensional difference gel electrophoresis [101,102] (see Materials and methods). In contrast, the levels of Sam1p actually fall with increasing growth rate (Figure 7, and Additional data files 1 (Figure S29) and 2 (Tables S23 and S25)).

Increased fluxes through C1 (folate) metabolism towards synthesis of 5-methyltetrahydropteroyltriglutamate, the donor of the terminal methyl group in methionine synthesis [42,103,104] were demonstrated by significant upregulation of *MET7 *(*q *for *MET7 *= 0.042), encoding folylpoly-glutamate synthetase (FPGS), which is responsible for polyglutamylation of folate coenzymes [103-105], and upregulated levels of methylenetetrahydrofolate reductase, Met13p (see Figure 7 and Additional data file 2 (Table S22)). Here, it is noteworthy that *S. cerevisiae *cells possess only one methionine synthetase, Met6p, which functions without cobalamin as a cofactor [42,103,104]. These results show the relevance of controlled fluxes of glutamate in methionine and SAM synthesis and point to the existence of close interrelations between the carbon, nitrogen and sulfur central metabolic pathways. The complete picture is one where an increase in growth rate involves the mobilization of C1 and sulfur metabolism towards increasing synthesis of SAM and proteins (see Figure 7).

We analyzed the growth-rate response of transcripts encoding methyltransferases and found those responsible for the methylation of rRNA (for example, *NOP1*, *NOP2*, *SPB1 *and *DIM1*) and tRNA (for example, *NCL1*, *TRM1*, *TRM3*, *TRM7*, *TRM8*) to be overrepresented in the group of genes whose transcription is significantly upregulated with growth rate (*q *< 0.09; from the ANCOVA analysis). Control of rRNA and tRNA synthesis (including rRNA and tRNA methylation) is closely tied to cell growth [106]. From reports in the literature, we have calculated that more than 2,000 methylation events per second are required just for the *de novo *synthesis of rRNA [107,108]. Thus, high growth rates will generate a high demand for SAM simply to sustain the methylation of rRNAs and tRNAs, let alone the requirements associated with the methylation of the GpppN termini of capped mRNAs [109]. Our data indicate that the increasing levels of Sam2p are most likely to satisfy this demand at high growth rates, thus associating Sam2p with the high-turnover pool of SAM. In contrast, Sam1p may have the main responsibility for the redistribution of SAM between the different subcellular organelles. A definitive attribution of the division of responsibility for the production of this key metabolite between the two isoenzymes must await more advanced studies involving selective labeling and *in vivo *imaging.

**Figure 8:**
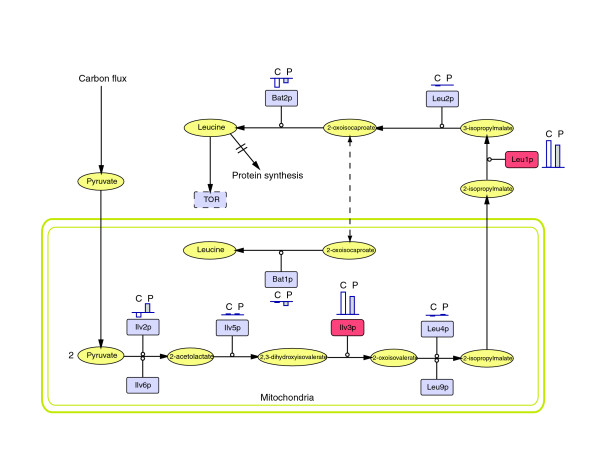
Multiple enzyme regulation in the metabolic control of the leucine biosynthetic pathway at the protein level. *In vivo* relative changes in enzyme levels from μ = 0.1 to 0.2/h, under carbon- (C) and phosphate- (P) limiting conditions are indicated by the lengths of the gray and white bars next to each enzyme. Their position under or above the baseline indicates downregulation or upregulation, respectively. Leucine biosynthesis diagram and nomenclature from [42]; diagram drawn with Cell Designer [136] and Adobe Illustrator [137]. Enzymes consistently upregulated under both conditions are marked in red. Metabolic steps (enzymes): Ilv2p, acetolactate synthase; Ilv6p, regulatory subunit of acetolactate synthase; Ilv5p, acetohydroxyacid reductoisomerase; Ilv3p, dihydroxyacid dehydratase; Leu4p, 2-isopropylmalate synthase (main isozyme); Leu9p, 2-isopropylmalate synthase (minor isozyme); Leu1p, isopropylmalate isomerase; Leu2p, 3-isopropylmalate dehydrogenase; Bat1p, mitochondrial branched-chain aminoacid aminotransferase; Bat2p, cytosolic branched-chain amino-acid aminotransferase.

The above examples show that integration of transcriptomic, proteomic, and metabolomic studies can provide detailed information about cellular strategies to direct metabolic fluxes toward the supply of intermediates required to sustain cell growth. However, a key question remains unanswered: how is the control of metabolic flux shared between regulation at the level of gene expression (that is, enzyme expression levels) and regulation at the level of metabolism itself, where individual intermediates can alter enzyme activity? In Figure 8, we show the impact of a change in growth rate (from 0.1 to 0.2/hour, in carbon- and phosphate-limited chemostat cultures) on the relative levels of the enzymes involved in the biosynthesis of leucine, an amino acid that has been reported as an upstream regulator of the TOR pathway [31].

These data show that, within a particular metabolic pathway, some enzymatic steps may be selectively regulated at the level of enzyme production (for example, Ilv3p, Leu1p), whereas others exhibit negligible regulation at the protein level (for example, Ilv5p). Our results support the 'hierarchical' control concept encompassed by regulation analysis theory [110] that has been used previously to explain the control of glycolysis in yeast [111,112]. What is now required is some convenient approach that will permit a global, systematic integration of metabolome data with those of transcriptomics and proteomics, rather than the case-by-case analysis that we have presented here.

The above examples show that integration of transcriptomic, proteomic, and metabolomic studies can provide detailed information on cellular strategies for control of metabolic fluxes. They have revealed the existence of differentially regulated isoenzymes, which make complementary contributions to metabolic flux at different growth rates. This integrative approach, and the information obtained from it, opens the way for systems biology to exploit new theories (such as regulation analysis theory [110]) that derive from the concepts of metabolic control analysis. They also suggest novel strategies for the comprehensive metabolic engineering of yeast, one of the great workhorses of both ancient and modern biotechnology.

## Conclusions

The principle of multilevel regulation in metabolic control analysis has been explored in a systems biology study of the eukaryotic cell. We have measured the impact of changes in growth rate on the transcriptome, proteome, and endo- and exometabolomes of the yeast *S. cerevisiae*. Each level shows clear growth-rate-associated trends and also discriminates between different nutrient-limiting conditions.

Characteristic signatures at the transcriptomic, proteomic, and exo- and endo-metabolomic levels reveal groups of growth-regulated genes, proteins and biological processes controlled at different levels, with specific outliers characteristic for each nutrient-limiting condition. A large proportion of the growth-regulated yeast genes share protein orthologs with other eukaryotes, including humans, which points to the existence of an essentially conserved core protein machinery governing cell growth in Eukarya. A high proportion (72.5%) of these growth-regulated genes appear to be targets for the TORC1 signaling pathway and participate in a high number of protein-protein interactions.

Our studies emphasize the importance of TOR as a central controller that integrates not only the sensing of nutritional status, but also the response of all aspects of gene expression, including transcription, translation and protein stability [3,31,32,55,113]. One relevant design rule [114] elucidated by this study and data from our category 2 experiments (D. Delneri and S.G.O., unpublished work) is that major controllers of eukaryotic cell growth (such as TOR), are not themselves subject to growth-rate control at the transcriptional level. This appears to be a general trend in biology. This, and the fact that genome-wide studies aimed to extract all genes regulated under one condition fail to detect genes known to make a significant phenotypic contribution in the same condition [41,115,116], points to the need for incorporation of new integrative strategies in order to identify groups of genes controlled at different regulatory levels.

At the gene-expression level, comprehensive integration of quantitative proteome and transcriptome data reveals the widespread extent and importance of post-transcriptional mechanisms in growth-rate control, specific for each nutrient-limiting condition, and suggests that the translational efficiencies of particular mRNAs are modulated selectively in order to fine-tune protein activities and metabolic fluxes during cell growth. Our studies open the way towards the dissection of the contribution of transcriptional and translational control of gene expression in genome-wide studies.

Control of metabolic fluxes at the level of enzyme concentration is demonstrated by the integration of quantitative proteomic and metabolome studies. The regulation of metabolic flux appears as a dynamic process, involving distributed control at the transcriptional, translational and post-translational levels, and fine-tuning at the level of the metabolites themselves [117,118]. This would appear to be one of the sources of the intrinsic adaptability and distributed robustness of the eukaryotic cell, allowing it to adapt to short- and long-term environmental changes [119].

In all, the results reveal a multilevel, fine regulation of gene expression and metabolic fluxes during cell growth. Our results have direct implications in advanced studies on cell growth, *in vivo *regulation of metabolic fluxes for comprehensive metabolic engineering, and the design of genome-scale systems biology models of the eukaryotic cell.

## Materials and methods

### Yeast strain and experimental strategy

The diploid *Saccharomyces cerevisiae *strain FY1679 (*MATa/MATα ura3-52/ura3-52 leu2-1/+trp1-63/+his3-D200/+ho::kanMX4/ho::kanMX4 *was used for all the experiments. Conditions for chemostat cultivation in a mineral medium under C, N, P and S nutrient limitation have been described previously [14,120] and are given in Additional data file 4). For the rapamycin study, a culture growing at mid-exponential phase was divided into two. Rapamycin (200 ng/ml) was added to one half, and the drug's solvent to the other, as the control. Samples were taken at 0, 1, 2 and 4 h after treatment (see Additional data file 4).

### Transcriptional studies

Biomass was harvested and total RNA extracted as previously described [14]. For growth-rate dependence studies, the microarray experimental design consisted of four nutrient-limiting conditions grown at three growth rates. These 12 conditions were analyzed in quadruplicate using Affymetrix Yeast Genome S98 GeneChip oligonucleotide arrays (Affymetrix, Santa Clara, CA). For the rapamycin study, samples were analyzed in duplicate using YG_S98 arrays (Affymetrix). Arrays that passed outlier data-quality assessment using dChip software [121] were normalized with RMAExpress [122]. For each probe set the coefficient of variation (CV) was calculated for each condition (%CV = (standard deviation/mean) × 100). The mean CV (variance in transcriptional studies) was calculated, being in the range between 2.4-3.9% in all cases. The data were submitted in MIAME-compliant format to the ArrayExpress public repository [123] under accession numbers E-MEXP-115 (growth-rate studies) and E-MAXD-4 (rapamycin studies).

Statistical analyses (PCA, *t*-tests, ANOVA/ANCOVA and false-discovery rate estimation) to identify significantly regulated genes were performed with Matlab (MathWorks, Natick, MA) [124], Q-value [40], GeneSpring (Agilent Technologies, Santa Clara, CA) [125] and maxdView software (available from [126]); see Additional data file 4 for details. Transcriptome results were validated by comparison of the patterns of gene expression of genes from equivalent chemostat experiments using an independent macroarray technique [19] and, in addition, triplicate analyses of eight genes were performed by QRT-PCR.

### Proteome studies

Yeast were grown in chemostat culture as described above. Cycloheximide (final concentration 100 μg/ml) was added to 1-liter steady-state chemostat cultures. Cells (10 × 80 ml) were harvested and centrifuged at 5,000 rpm for 5 minutes at 4°C. The pellet was resuspended in 1 ml ice-cold double-distilled water and transferred to a 1.5-ml microcentrifuge tube. Cells were repelleted by centrifugation at 10,000 rpm, the supernatant discarded, and the yeast pellet frozen in dry ice and stored at -80°C.

#### Isotope tags for multiplexed relative and absolute quantification (iTRAQ) of proteins

Protein samples were precipitated as follows. Chilled acetone (1.8 ml) was added to a 300 μl sample. The tubes were inverted three times and left at -20°C for 4 h. Precipitated proteins were pelleted by a 10 minute centrifugation at 3,000 rpm, and resuspended in iTRAQ labeling buffer (8 M urea, 2% Triton X100, 0.1% SDS and 25 mM triethyl ammonium bicarbonate (TEAB) pH 8.5). Protein concentration was determined using the detergent-compatible BCA protein assay (Pierce, Rockford, IL).

Each sample (100 μg protein) was then reduced (4 mM Tris(2-carboxyethyl) phosphine (TCEP), 20°C, 1 h) and cysteines blocked (8 mM methyl methanethiosulfonate (MMTS), 20°C 10 minutes). Samples were then diluted with 50 mM TEAB (pH 8.5) such that the final urea concentration was below 1 M, digested with trypsin (1:20) overnight at 37°C (Promega, Madison, WI; 2.5 μg added at 0 and 1 h) and lyophilized using a Savant AES2010 speed vacuum system.

Three separate iTRAQ labeling experiments were carried out such that each sample corresponding to a nutrient limitation and growth rate was labeled once. In each experiment one of the iTRAQ tags was used to label a pooled sample comprising equal amounts of each sample analyzed within the experiment (see Additional data file 1 (Figure S28) for the iTRAQ labeling scheme). Each lyophilized sample was resuspended in 100 μl labeling buffer (0.25 M TEAB, 75% ethanol), added to one unit of the corresponding iTRAQ reagent and incubated for 1 h at 20°C. Residual reagent was quenched by adding 100 μl water and incubating for a further 15 minutes at 20°C. The samples belonging to the iTRAQ comparisons were then pooled (pooled standard) and lyophilized.

#### Cation exchange chromatography

Cation exchange fractionation of the iTRAQ samples was carried out on a BioLC HPLC system (Dionex, Sunnyvale, CA) using a polysulphoethyl A column (PolyLC, Columbia, MD; 2.1 × 200 mm, 5 μm, 300 Å). The three lyophilized iTRAQ-labeled pools were resuspended in 6 ml 20% vol/vol acetonitrile (ACN), 10 mM KH_2_PO_4_-H_3_PO_4 _pH 2.7, loaded and washed isocratically for 60 minutes at a flow rate of 200 μl/minute to remove the urea, detergents, and excess reagents. Peptides were eluted using a linear gradient of 30-125 mM KCl (20% vol/vol ACN, 10 mM KH_2_PO_4_-H_3_PO_4 _pH 2.7) over 70 minutes at a flow rate of 200 μl/minute. Fractions were collected at 2-minute intervals, lyophilized and resuspended in 70 μl 2% ACN, 0.1% trifluoroacetic acid. Fractions 13-36 (collected between 65 mM and 105 mM KCl) from each iTRAQ experiment (72 fractions in total) were analyzed by liquid chromatography followed by tandem mass spectrometry (LC-MS/MS).

#### LC-MS/MS analysis

Peptides were separated and analyzed using an Ultimate Plus nano-LC system (Dionex) coupled to a QSTAR XL quadrupole TOF hybrid mass spectrometer (Applied Biosystems, Foster City, CA). Samples (60 μl) were loaded onto an Acclaim PA C16 pre-column (5 mm × 300 μm internal diameter, Dionex) at 20 μl/minute and washed with 0.1% formic acid (FA; also at 20 μl/minute) for 25 minutes to desalt the samples. Peptides were then eluted onto a PepMap C18 analytical column (15 cm × 75 μm internal diameter, Dionex) at 150 nl/minute and separated using a 165 minute gradient of 5-32% ACN (0.1% FA). The QSTAR XL was operated in information-dependent acquisition (IDA) mode, in which a 1 second TOF-MS scan from 400-1,600 m/z was performed, followed by 3 second product ion scans from 100-1,580 m/z on the two most intense doubly (2^+^) or triply (3^+^) charged ions.

#### MS data analysis and protein quantification

Mass spectrometry data files were processed using the wiff2DTA software to generate centroided and uncentroided peak lists [127]. Mascot version 2.0.01 (Matrix Science, London, UK) was used to search centroided peak lists against the *Saccharomyces *Genome Database protein database (latest release). The following modifications were used: fixed, iTRAQ (K), iTRAQ (N-term), MMTS (C); variable, oxidation (M), iTRAQ (Y). The MS tolerance was 0.2 Da and the MS/MS tolerance 0.5 Da. Each peak list was also searched against a reversed version of the database in order to determine the false identification rate. Mascot peptide score thresholds of 29 for all three experiments resulted in false identification rates of less than 1% for proteins with at least two peptides. iTRAQ reporter ion ratios were calculated from the uncentroided peak lists using i-Tracker [35,128]. Normalized reporter ion areas were calculated as follows: normalized area A = area A/(area A + area B + area C + area D).

The Genome Annotating Proteomic Pipeline (GAPP) system [129] was used to parse peptide identification and scoring information from the Mascot output files and link these to the quantification data in a relational database (MySQL version 4.0, MySQL, Uppsala, Sweden). Peptides were quantified if at least three of the four reporter ion peaks were above a threshold of 15 counts and if they had a Mascot score of at least 20. In addition, only peptides that were unique to a single identified protein were quantified. After application of this strategy, we were able to quantify a significant proportion of the yeast proteome (around 700 proteins per nutrient-limiting condition; 584 common to all nutrient-limiting conditions; 1,358 unique proteins in total). The global coefficient of variance of the proteomics data was calculated using replicate pools and never exceeded 13%. Proteomics (iTRAQ) results were validated by two-dimensional difference gel electrophoresis [101,102], confirming clear growth-rate trends at the global level (by PCA) and consistent trends in patterns of expression of representative relevant proteins (for example, Aco1p, Cpa2p, Rpl5p, Met6p, Sam2p, Ado1p) (data not shown). Proteomic data are in the process of being submitted to the PRIDE proteomics repository [130].

### Metabolome studies

Sampling, quenching, and efficient extraction for endo- and exometabolome analyses of hundreds of intra- and extracellular metabolites, including the main glycolytic intermediates, nucleotides, pyridine nucleotides, and organic acids (for example, pyruvate, citrate, and succinate) were carried out as described previously [131]. Direct quenching of the culture was performed by fast sampling of a volume of culture equivalent to 30 mg dry weight [131]; extracts and culture supernatants were stored at -80°C. Samples were prepared for MS immediately before the analysis was carried out.

#### GC/TOF-MS analysis

Two different sample types were analyzed, endometabolome and exometabolome. Endometabolome samples (typically 200 μl) were spiked with 4 μl internal standard solution (2.22 mg/ml [^2^H_2_]malonic acid, 1.92 mg/ml [^2^H_5_]glycine and 0.61 mg/ml [^13^C_6_]glucose dissolved in water) and lyophilized using a Speedvac concentrator vacuum system SPD111V connected to a Micromodulyo Freeze Dryer (ThermoLife Sciences, Basingstoke, UK). Exometabolome samples (1,000 μl) were spiked with 20 μl internal standard solution and lyophilized as previously described. Dried samples were derivatized as follows; 100 μl of 20 mg/ml *O*-methylhydroxylamine solution was added and heated at 40°C for 90 minutes followed by addition of 100 μl *N*-acetyl-*N*-(trimethylsilyl)-trifluoroacetamide and heating at 40°C for 120 minutes. The final solution was spiked with 20 μl retention index solution (0.6 mg/ml *n*-decane, *n*-dodecane, *n*-pentadecane, *n*-nonadecane, *n*-docosane dissolved in hexane).

All samples were analyzed on an Agilent 6890 gas chromatograph coupled to a LECO Pegasus III time-of-flight mass spectrometer (LECO, St Joseph, MI) using the manufacturer's software (ChromaTOF version 2.12) and a DB-50 GC column (Supelco, Gillingham, UK; 30 m × 0.25 mm × 0.25 μm film thickness). The instrument conditions are detailed in Additional data file 2 (Table S30). In the ChromaTOF software the S/N threshold was set at 10, baseline offset at 1.0, data points for averaging at 7, and peak width at 2.5. The TOF mass spectrometer can collect spectra at up to 500 Hz and uses sophisticated but proprietary deconvolution software to discriminate overlapping peaks on the basis of their mass spectra. Initial processing of raw data was undertaken using LECO ChromaTOF v2.12 software to construct a data matrix (metabolite peak versus sample number) using response ratios (peak area metabolite/peak area [^2^H_2_]malonic acid) to calculate the relative amount of each metabolite in each sample. For each metabolite peak for each set of three biological replicates, the CV was calculated as follows: %CV = (standard deviation/mean) × 100. The mean CV for replicate analytical analyses (*n *= 3) was 24.6% for all analyses undertaken, with 62% of these analyses having a CV less than 20% and 38.04% having one less than 10%. Although this shows precision to be less robust than for targeted analyses, this is appropriate in metabolic profiling, where many hundreds of metabolites are detected in short analysis times.

Metabolite peaks were initially identified by searches on a commercially available mass spectral library [NIST/EPA/NIH (02)] (US National Institute of Standards and Technology, the Environmental Protection Agency and the National Institutes of Health; 2002) and libraries prepared by one of us (W.B.D.). For a peak to be identified required a similarity and reverse match score of greater than 700. Metabolite identification was confirmed by the analysis of pure chemical standards in identical conditions to the sample analysis. Identification was confirmed if the retention time (± 5 s) and mass spectra (similarity and reverse matches greater than 750) of metabolite peak in sample and standard were equivalent. A list of profiled metabolites will be available on request at the Manchester Centre for Integrative Systems Biology website [132] after all metabolomic data are structured and annotated in a machine-usable form for efficient data management (MeMo [133]) towards the implementation of a standard metabolomics framework [134].

#### Analysis of GC/MS raw data and metabolite levels

Within a GC/MS-based data matrix composed of response ratios (peak area-metabolite/peak area-internal standard), zero (or not detected) values can be obtained for any given metabolite peak caused by either of the following reasons: the metabolite is not present or is present at a concentration below the limit of detection; or the metabolite cannot be resolved from others in the chromatograph by the deconvolution software. In these cases, the following procedure was used to improve data structure for further univariate analysis techniques. If two of three replicates were zero values and the third replicate was a non-zero value, the third (non-zero) replicate was replaced with zero. If two of three replicates were non-zero values and the third replicate was a zero value, the zero value for the third replicate was replaced with the mean of the other two replicates.

#### Determination of relative changes in translational control efficiencies

To encompass all mechanisms involved in translational control and to quantify its global effect, we define the translational control efficiency of each mRNAi (Trlc Eff_i_) as the effective translation of each transcript into protein (encompassing synthesis and degradation processes; net P/R ratio (protein/mRNA) as follows:

Trlc Eff_i _= ([Protein_i_]/([mRNA_i_]) (see also Additional data file 7).

From protein-transcriptome correlation studies we can define the ratio of relative changes in protein versus transcript levels (ratio[(/)p/(/) tr]) as:

Ratio[(/)p/(/)tr]_i _= ([Protein_i_]_2_/[Protein_i_]_1_)/([mRNA_i_]_2_/[mRNA_i_]_1_)

Thus, as an example, when applied to relative changes between two growth rates, for example, 0.2 versus 0.1/h:

Ratio[(/)p/(/)tr]_i _= [(Protein_i _0.2/Protein_i _0.1)/(Transcript_i _0.2/Transcript_i _0.1)]_i _= ([Protein_i_] 0.2/[Protein_i_] 0.1)/([mRNA_i_] 0.2/[mRNA_i_] 0.1)

From here, as Equation (1) can be rearranged as:

Ratio[(/)p/(/)tr]_i _= ([Protein_i_]_2_/([mRNA_i_]_2_)/([Protein_i_]_1_/[mRNA_i_]_1_)

it follows that the Ratio[(/)p/(/)tr]_i _is numerically equal to the ratio of translational control efficiencies i, from condition 1 to condition 2:

Ratio[(/)p/(/)tr]_i _= (Trlc Eff)_2_/(Trlc Eff)_1 _= (Ratio Trlc Eff)_i_

See also Additional data file 7.

Relative changes in translational control efficiencies are obtained from microarray and proteomic studies. These compare relative changes in gene expression of the same individual transcript (or protein) between two different growth rates. In these one-to-one comparisons, systematic errors due to, for example, different labeling or hybridization efficiencies are minimized. Moreover, we have sought to reduce all sources of systematic error and a summary of the strategies applied is included below. Despite all these precautions, translational control efficiencies will always be dependent on the accuracy of the techniques used to determine relative changes in gene expression. To evaluate these data, one must take into account the CV obtained for each independent technique used. These are provided above.

### Minimization of systematic error

We sought to minimize sources of systematic error, first by careful experimental design (see above) and the application of the following strategies: use of steady-state chemostat cultures ensuring carefully controlled environmental conditions at each constant growth rate [14-17]; avoidance of prolonged cultivation studies (that is, keeping to below 60 generations) to eliminate risks of strain variability and/or mutational effects; fast sampling and growth-arrest methods avoiding environmental disturbances for proper transcriptome, proteome and metabolome analyses; for each omic analysis, all samples were processed by the same specialist researcher; careful analytical strategies and normalization methods (see Additional data file 4).

## Additional data files

The following additional data are available with this paper online. Additional data file 1 contains additional figures. Additional data file 2 contains additional tables. Additional data file 3 contains the legends for the additional figures and tables. Additional data file 4 contains supplementary methods, including data analysis and processing for PCA, transcriptome, proteome and metabolome data analyses and integrative studies, statistical analyses on omic datasets, GO studies, and analysis of protein-protein interactions. Additional data file 5 includes additional studies on transcriptional control of cell growth. Additional data file 6 contains proteome-transcriptome correlations. Additional data file 7 contains the main concepts of translational control efficiency and translational control. Additional data file 8 contains global patterns of relative changes in translational control efficiencies. The whole article (with links to the additional documents) will also be available at the Manchester Centre for Integrative Systems Biology website [132].

## Supplementary Material

Additional data file 1 Supplementary figures S1-S28. Click here for file

Additional data file 2 Supplementary tables S1-S30. Click here for file

Additional data file 3 Legends to supplementary figures and tables Click here for file

Additional data file 4 Supplementary methods. Click here for file

Additional data file 5 Additional studies on transcriptional control of cell growth. Click here for file

Additional data file 6 Proteome-transcriptome correlations. Click here for file

Additional data file 7 Main concepts on translational control efficiency and translational control Click here for file

Additional data file 8 Global patterns of relative changes in translational control efficiencies. Click here for file
