# Supplementary material for: Growth control of the eukaryote cell: a systems biology study in yeast
Source: J Biol. 2007 Apr 30;6(2):4. doi: 10.1186/jbiol54 (PMC2373899; doi:10.1186/jbiol54)
Supplement: Additional data file 1 — Supplementary figures S1-S28. [file jbiol54-S1.zip › Fig S23 SGOliver.pdf]

Temporal control of cell growth. TOR control of gene expression at the transcriptional level

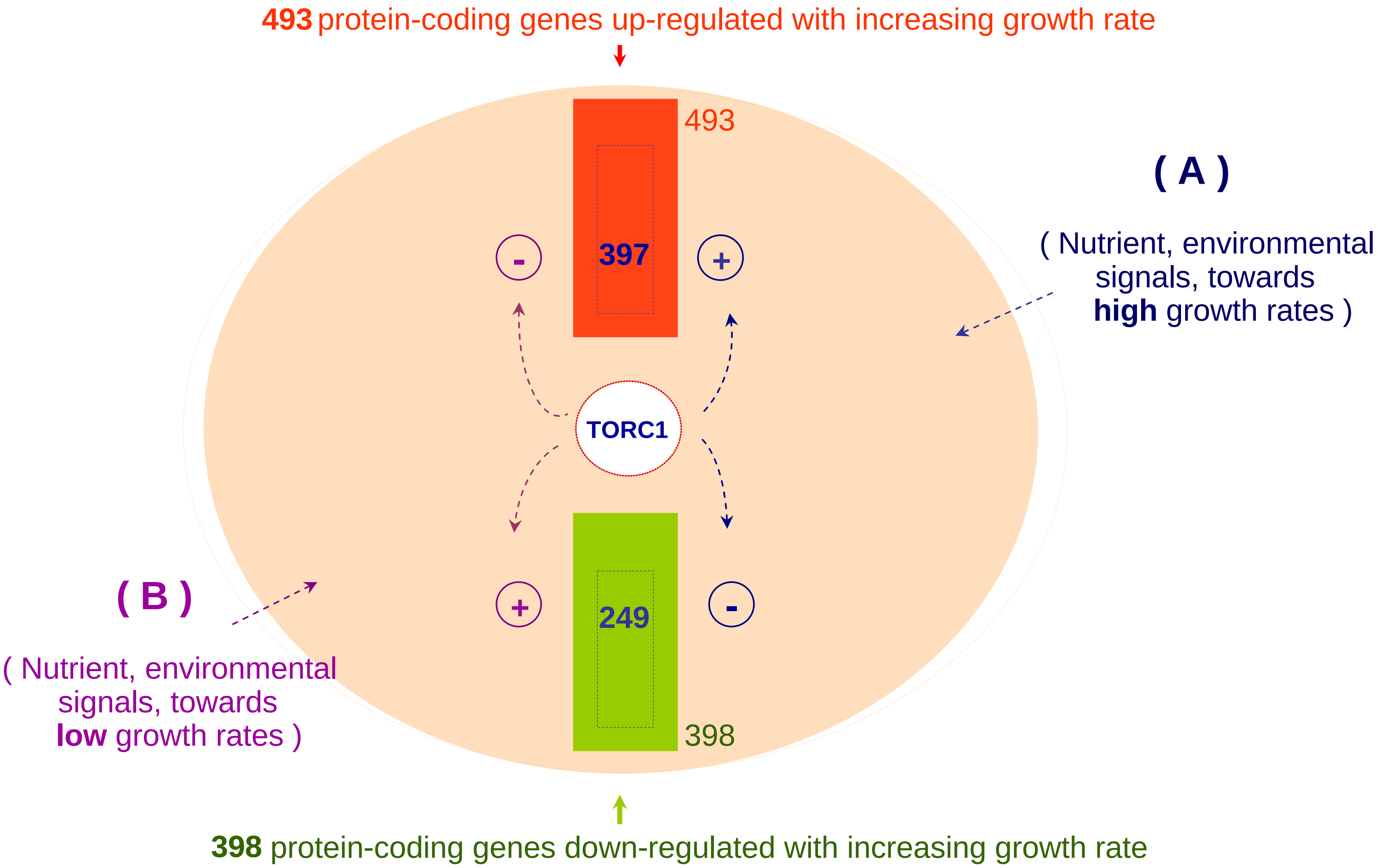

Fig. S23

Corresponding author: S. G. Oliver
