# Supplementary material for: Growth control of the eukaryote cell: a systems biology study in yeast
Source: J Biol. 2007 Apr 30;6(2):4. doi: 10.1186/jbiol54 (PMC2373899; doi:10.1186/jbiol54)
Supplement: Additional data file 1 — Supplementary figures S1-S28. [file jbiol54-S1.zip › Fig S28 SGOliver.pdf]

114	115	116	117	
P	N 0.1	C 0.1	S 0.1	EXPERIMENT 1
P	P 0.1	N 0.2	C 0.2	EXPERIMENT 2
P	S 0.2	P 0.2	P	EXPERIMENT 3

Fig. S28

Corresponding author: S. G. Oliver
