# Supplementary figures and images for: Growth control of the eukaryote cell: a systems biology study in yeast
Source: J Biol. 2007 Apr 30;6(2):4. doi: 10.1186/jbiol54 (PMC2373899; doi:10.1186/jbiol54)

# Biological process: down-regulated with growth rate

Fig. S8

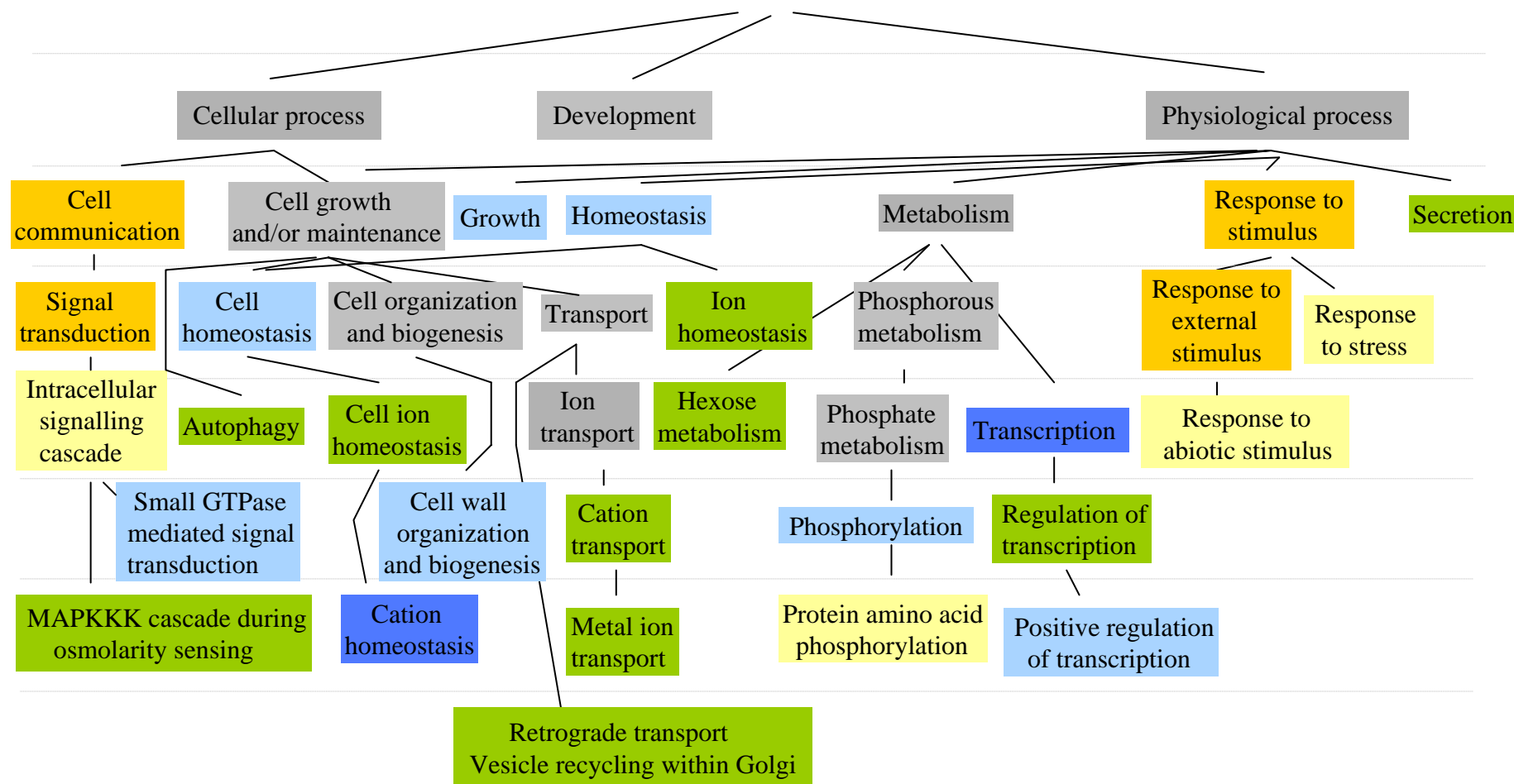

p value:

$\leq 2e^{-4}$

$2e^{-4}$  to  $4e^{-3}$

$4e^{-3}$  to  $1.5e^{-2}$

$1.5e^{-2}$  to  $5e^{-2}$

$> 5e^{-2}$

Supplement: Additional data file 1 — Supplementary figures S1-S28. [file jbiol54-S1.zip › Fig S8 SGOliver.pdf]

**Fig. S10**

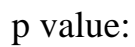
$$> 3e^{-1}$$

Supplement: Additional data file 1 — Supplementary figures S1-S28. [file jbiol54-S1.zip › Fig S10 SGOliver.pdf]

**Fig. S17**

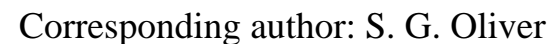

Supplement: Additional data file 1 — Supplementary figures S1-S28. [file jbiol54-S1.zip › Fig S17 SGOliver.pdf]

## RNA polymerase complex

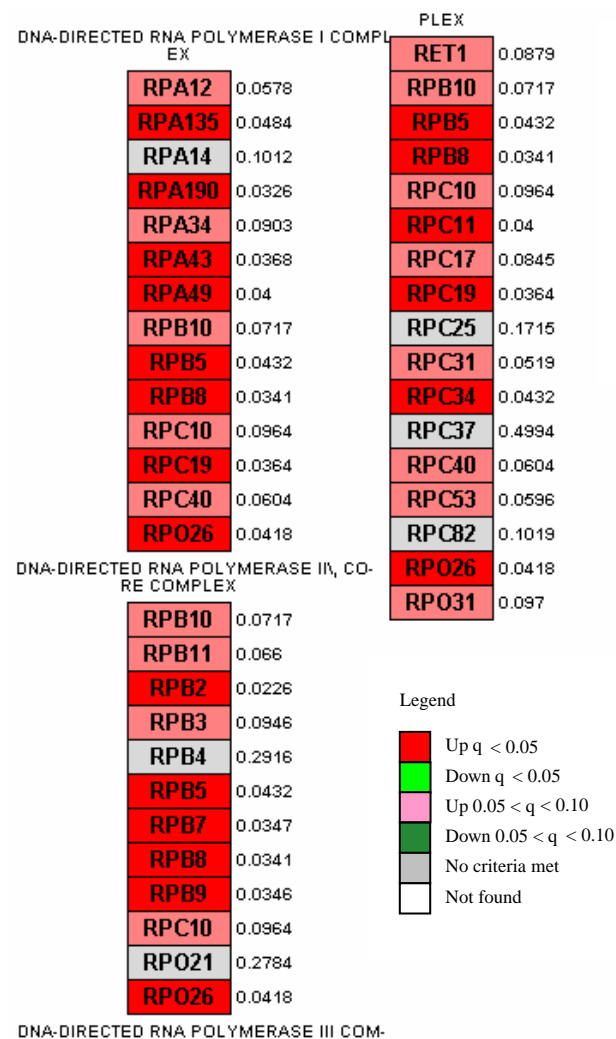

Fig. S18

Supplement: Additional data file 1 — Supplementary figures S1-S28. [file jbiol54-S1.zip › Fig S18 SGOliver.pdf]

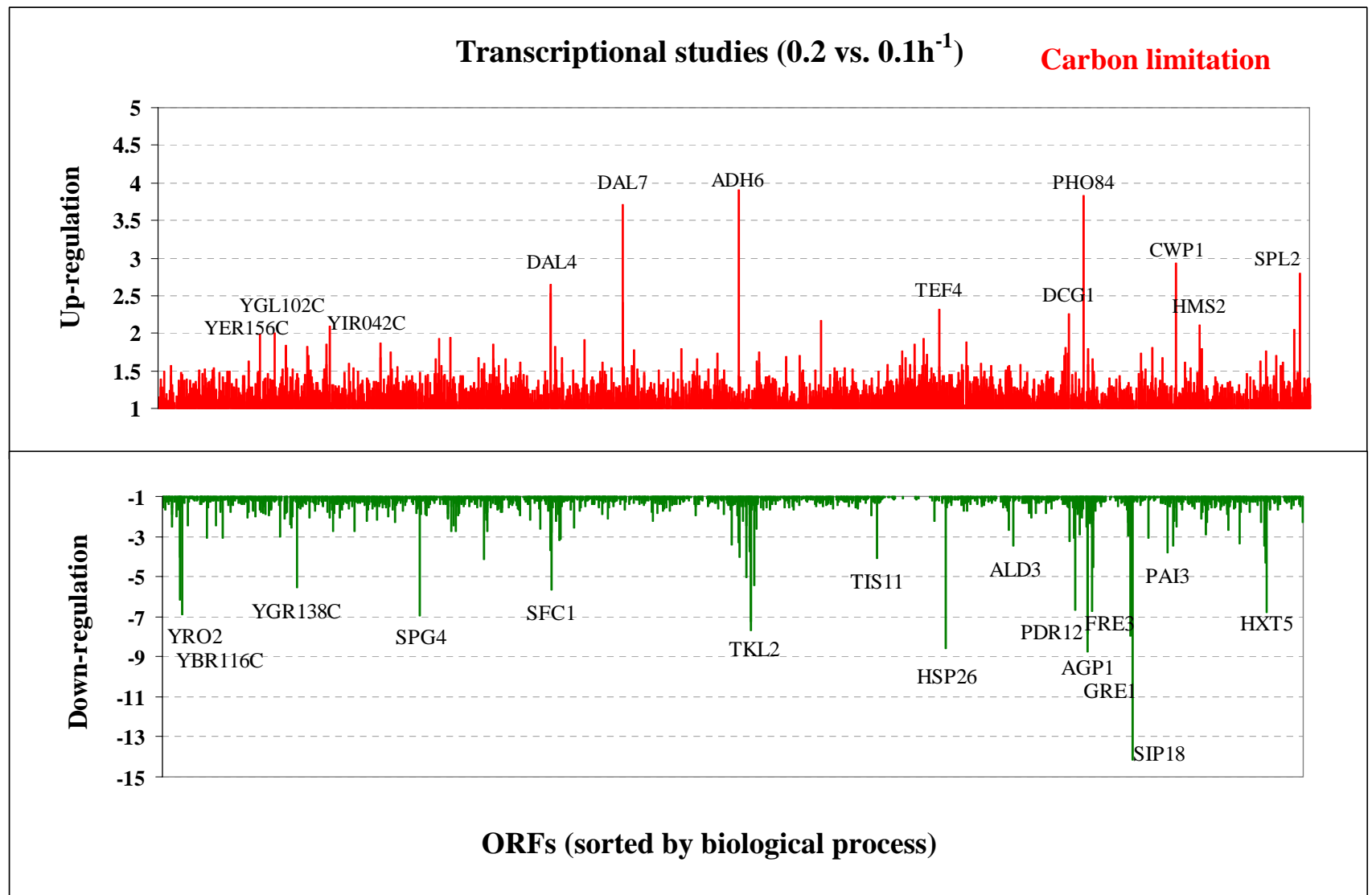

**Fig. S1**

Corresponding author: S. G. Oliver

Supplement: Additional data file 1 — Supplementary figures S1-S28. [file jbiol54-S1.zip › Fig S1 SGOliver.pdf]

## tRNA aminoacylation for protein translation

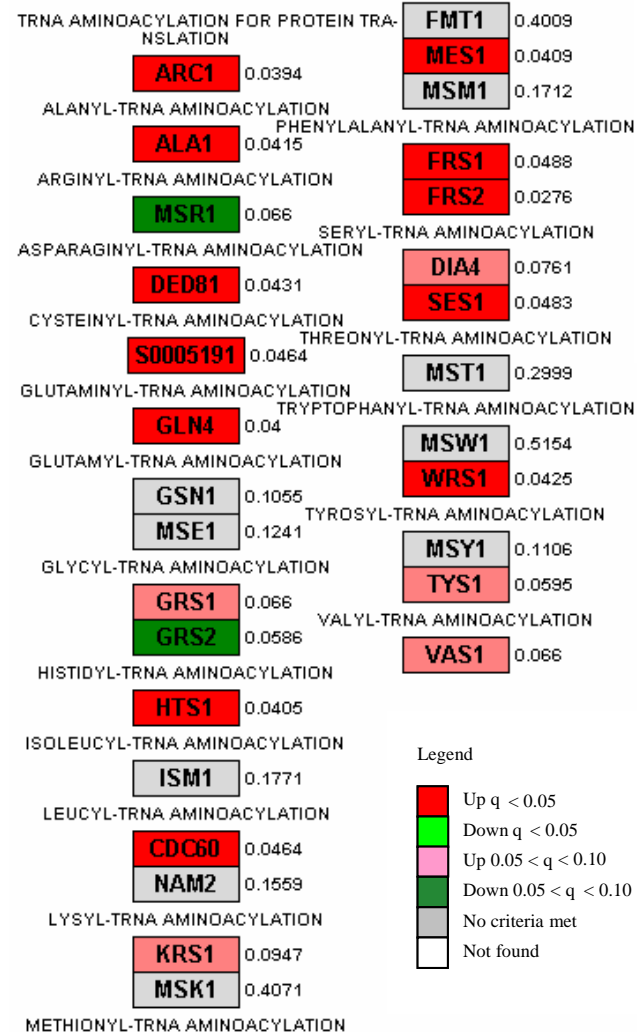

Fig. S19

Supplement: Additional data file 1 — Supplementary figures S1-S28. [file jbiol54-S1.zip › Fig S19 SGOliver.pdf]

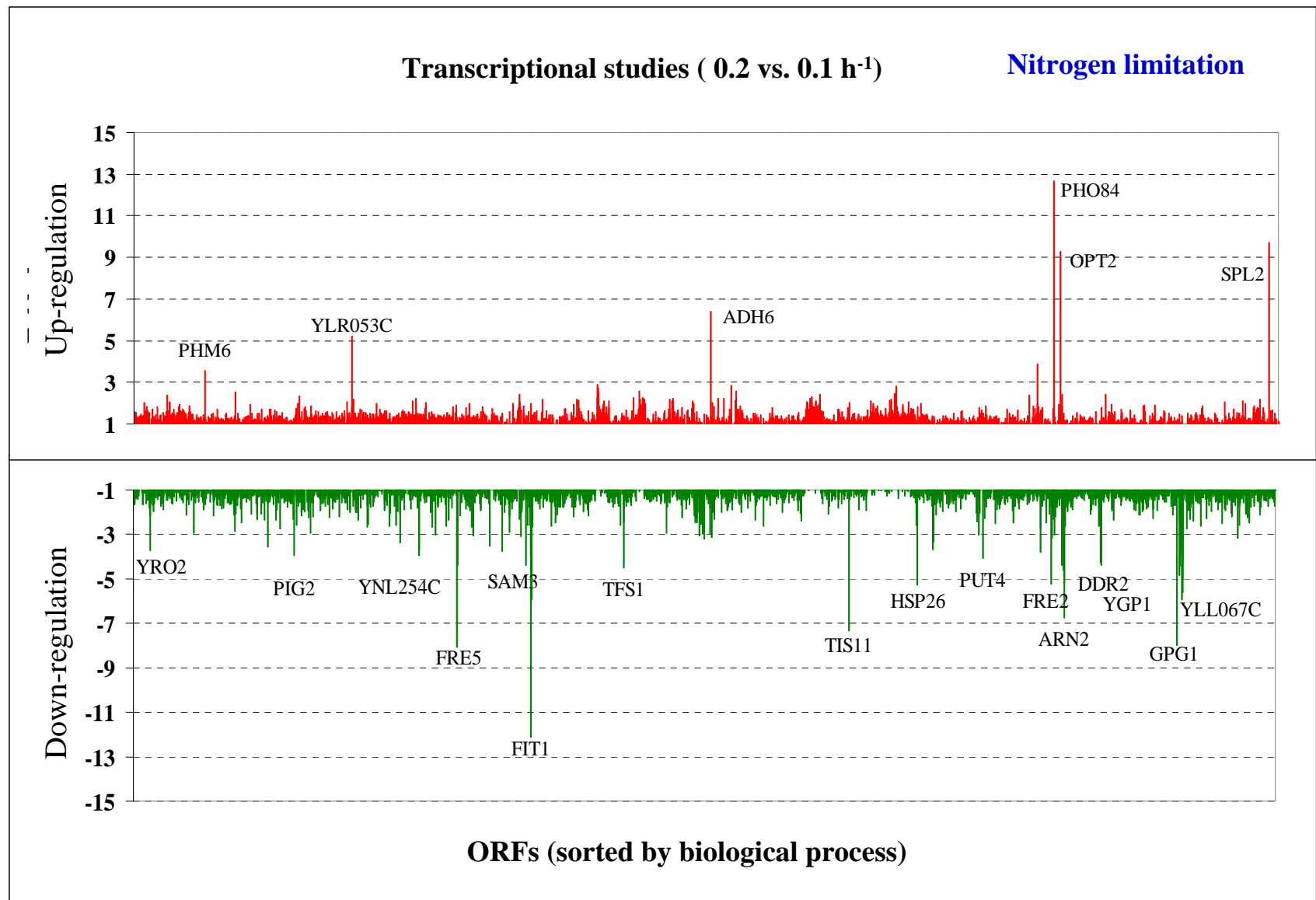

**Fig. S2**

Corresponding author: S. G. Oliver

Supplement: Additional data file 1 — Supplementary figures S1-S28. [file jbiol54-S1.zip › Fig S2 SGOliver.pdf]

## storage vacuole

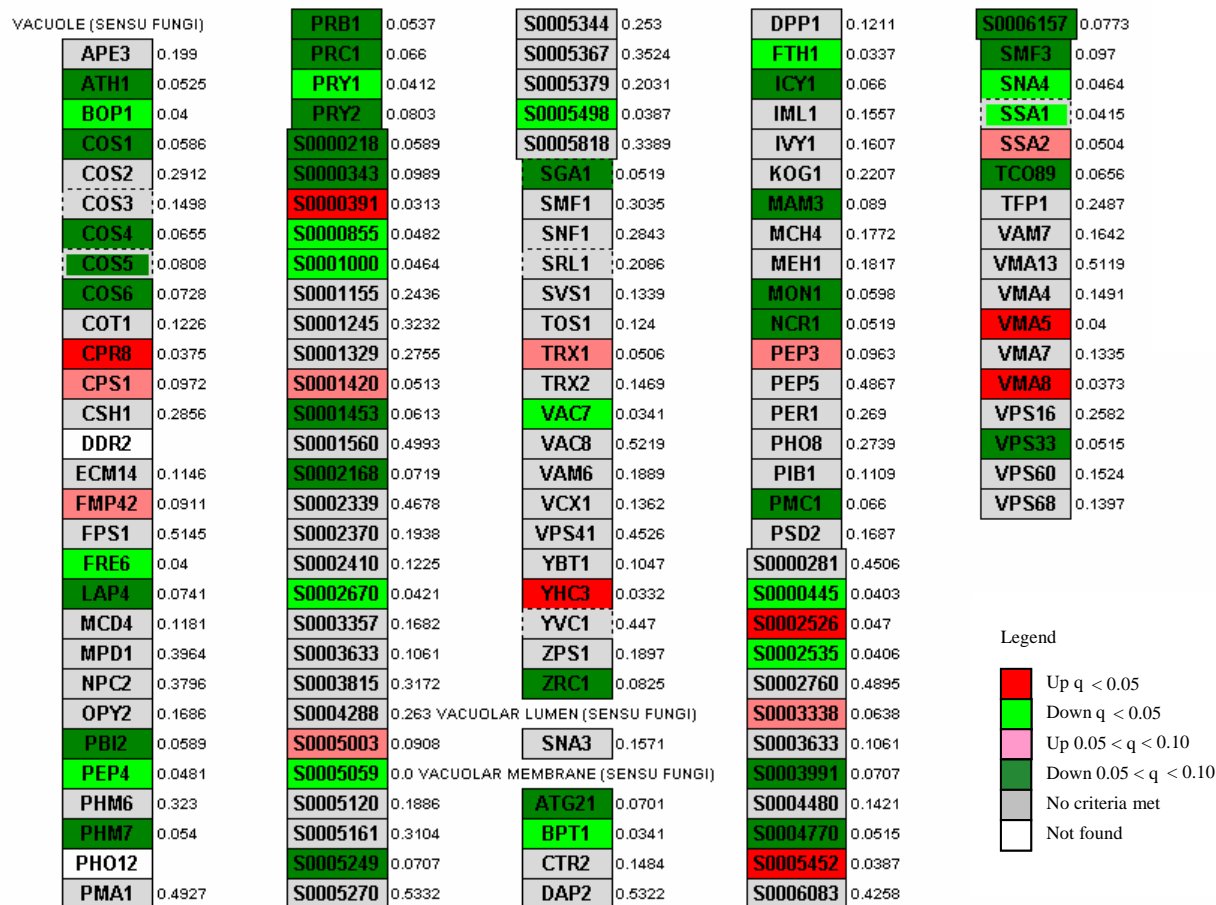

Fig. S20

Supplement: Additional data file 1 — Supplementary figures S1-S28. [file jbiol54-S1.zip › Fig S20 SGOliver.pdf]

# autophagy

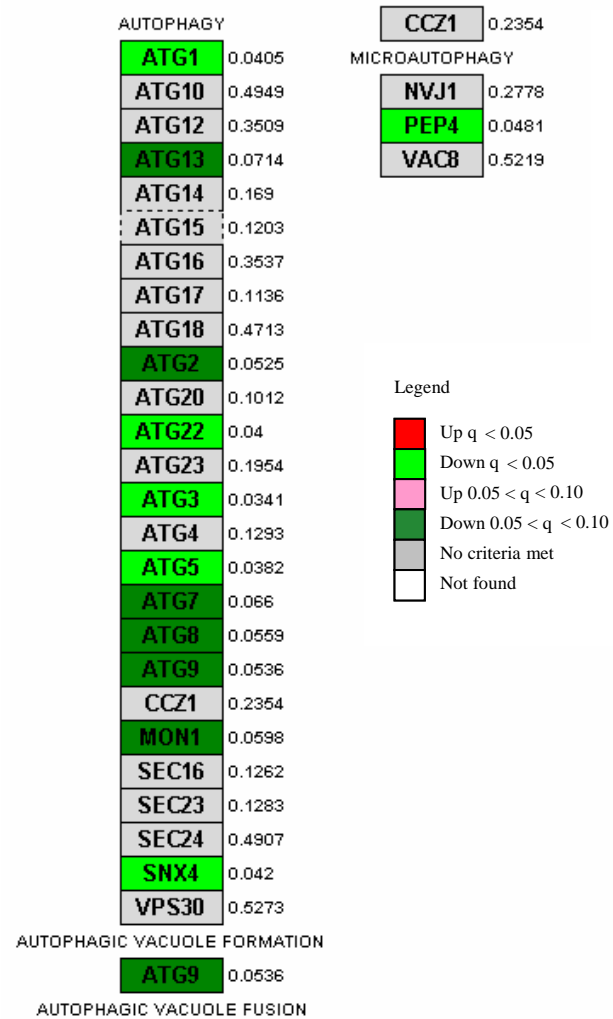

**Fig. S21**

Supplement: Additional data file 1 — Supplementary figures S1-S28. [file jbiol54-S1.zip › Fig S21 SGOliver.pdf]

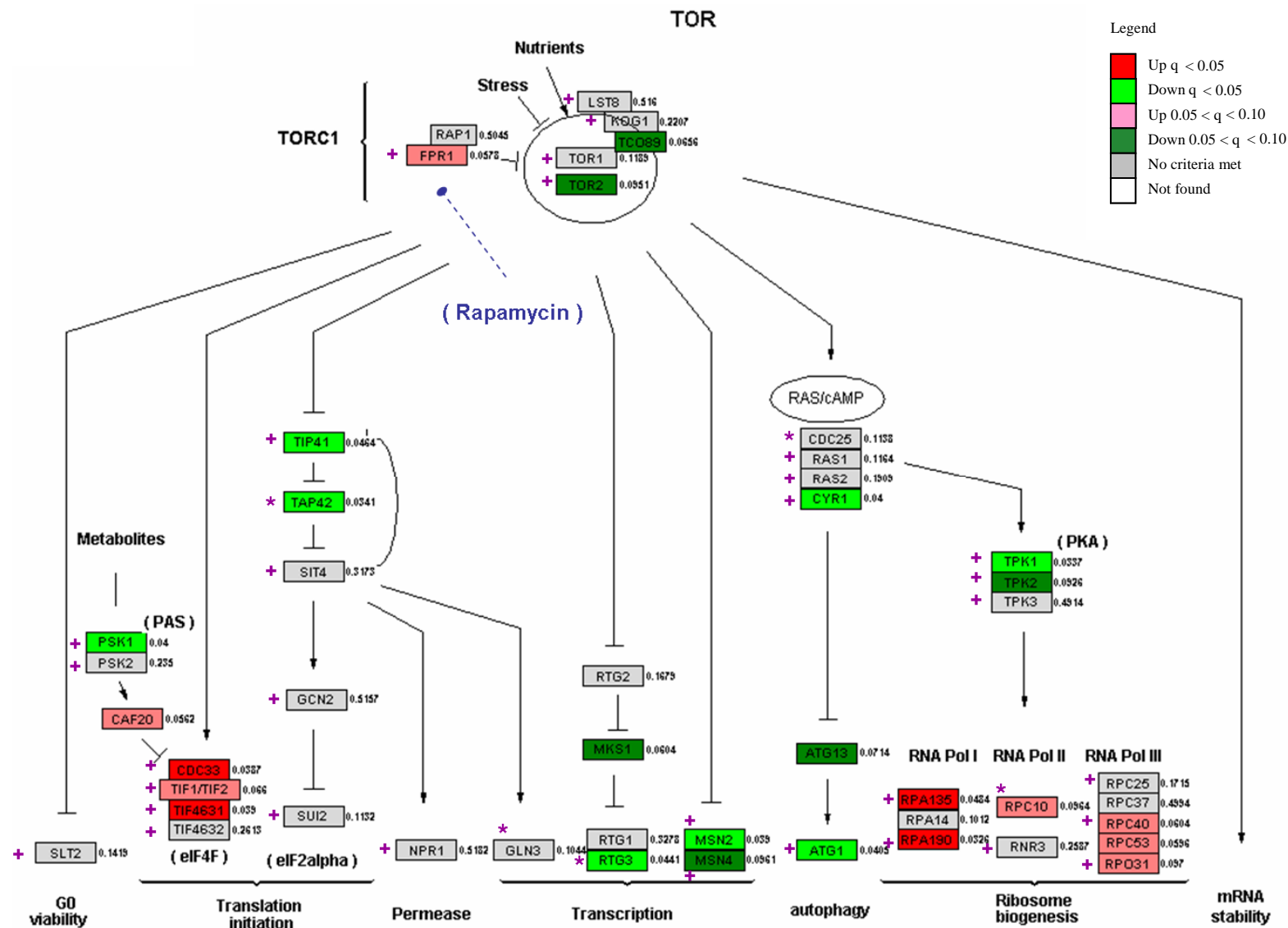

**Fig. S22**

Supplement: Additional data file 1 — Supplementary figures S1-S28. [file jbiol54-S1.zip › Fig S22 SGOliver.pdf]

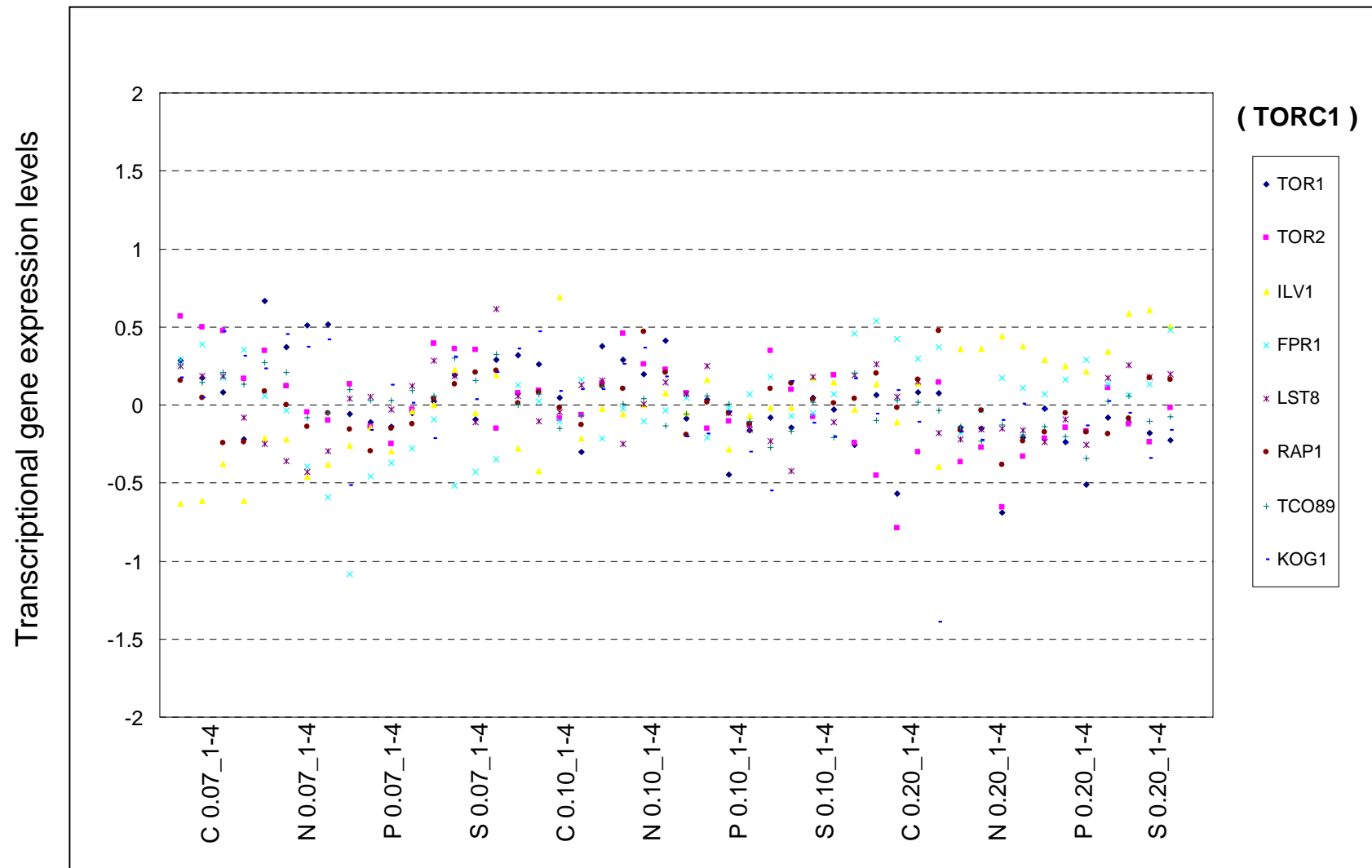

Fig. S24

Supplement: Additional data file 1 — Supplementary figures S1-S28. [file jbiol54-S1.zip › Fig S24 SGOliver.pdf]

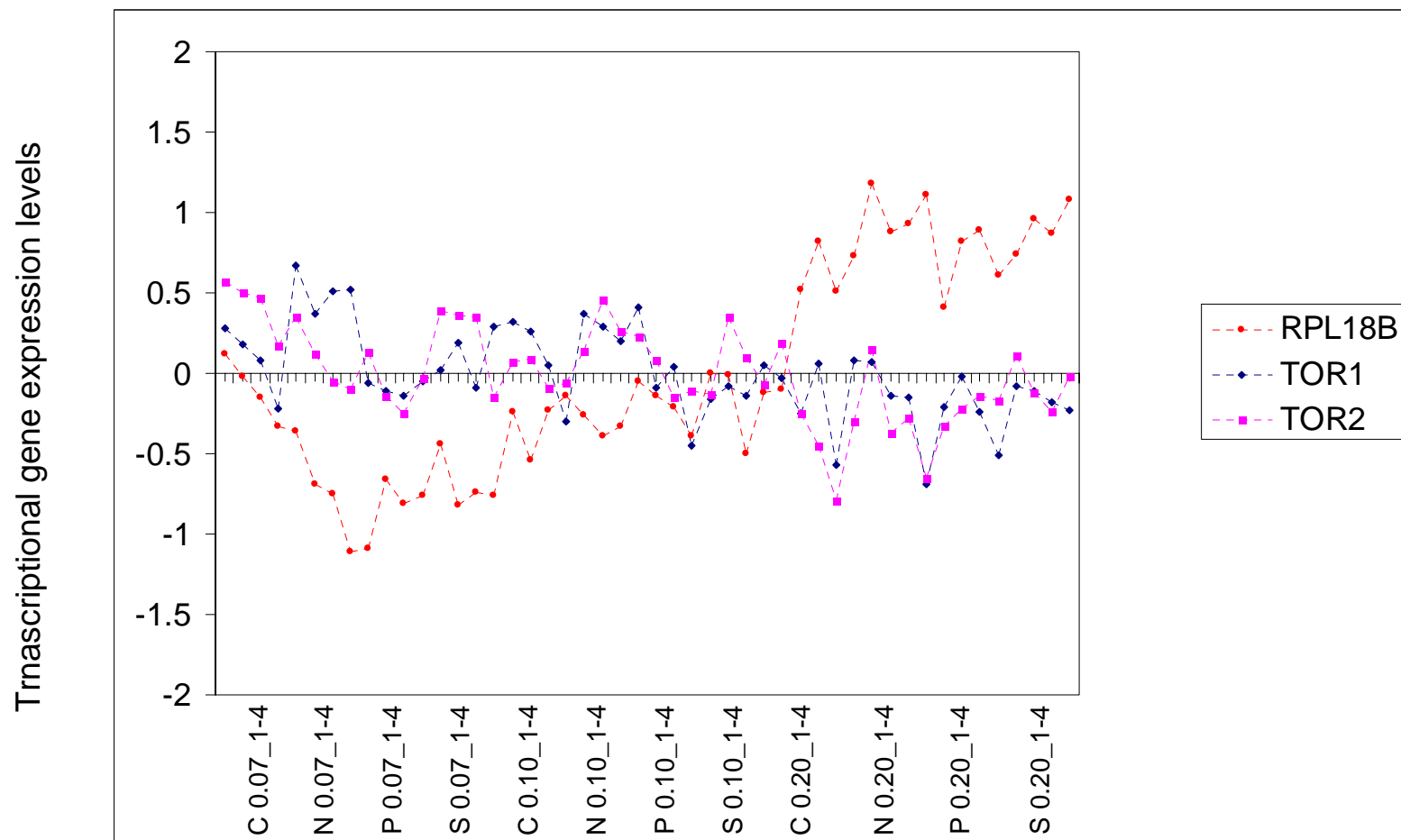

**Fig. S25**

Supplement: Additional data file 1 — Supplementary figures S1-S28. [file jbiol54-S1.zip › Fig S25 SGOliver.pdf]

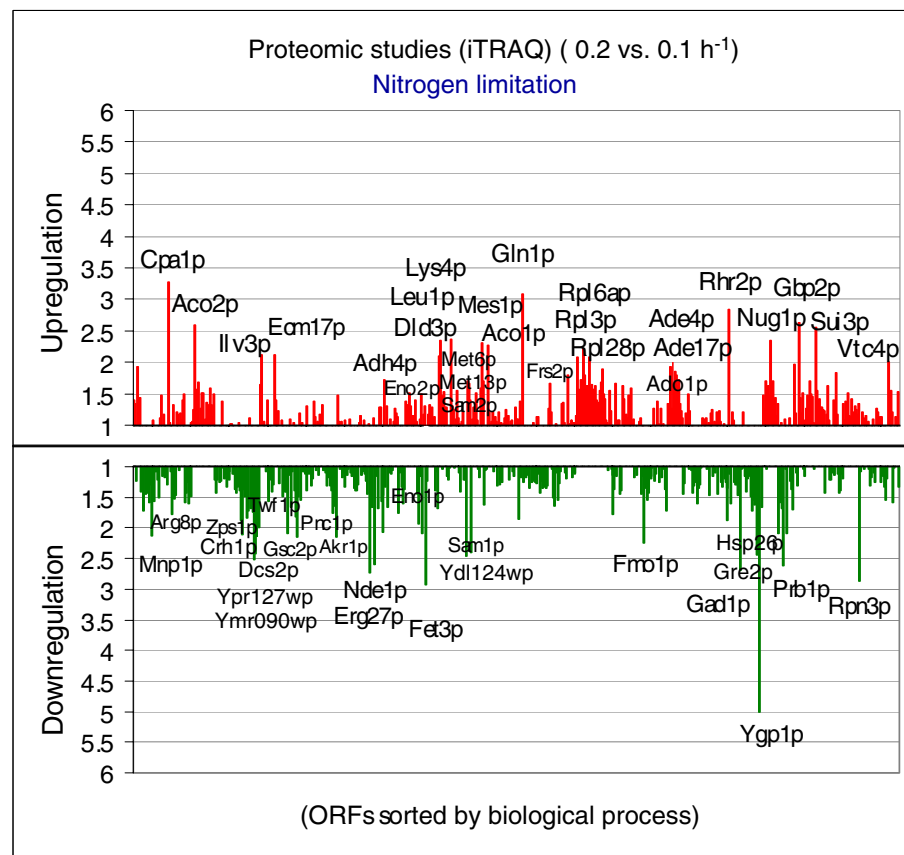

Fig. S26

Corresponding author: S. G. Oliver

Supplement: Additional data file 1 — Supplementary figures S1-S28. [file jbiol54-S1.zip › Fig S26 SGOliver.pdf]

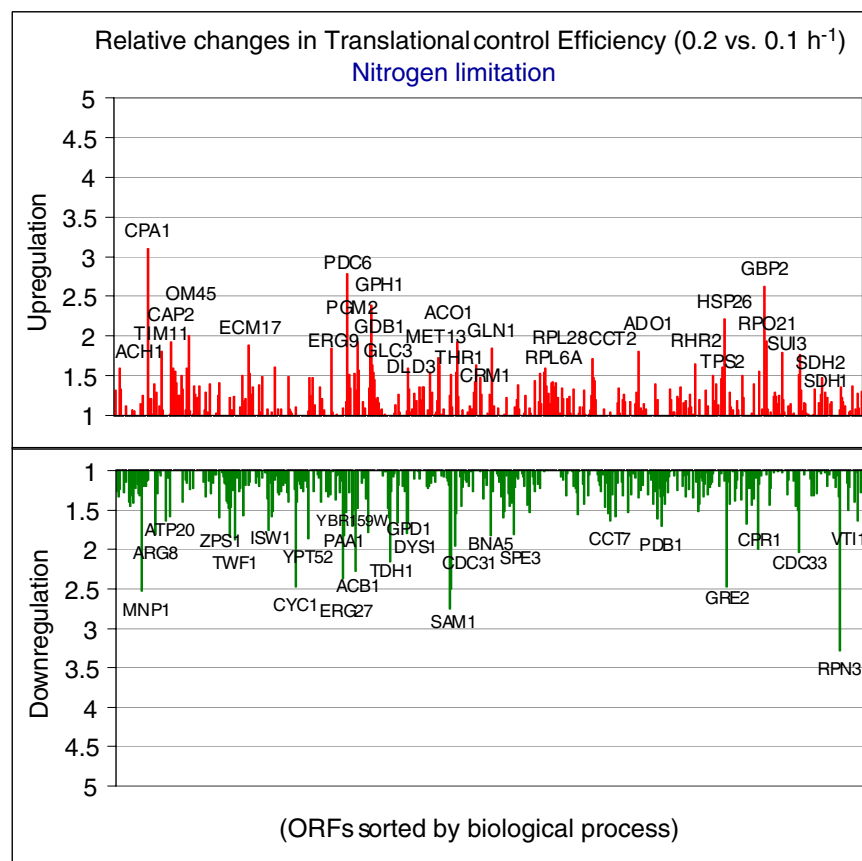

Fig. S27

Corresponding author: S. G. Oliver

Supplement: Additional data file 1 — Supplementary figures S1-S28. [file jbiol54-S1.zip › Fig S27 SGOliver.pdf]

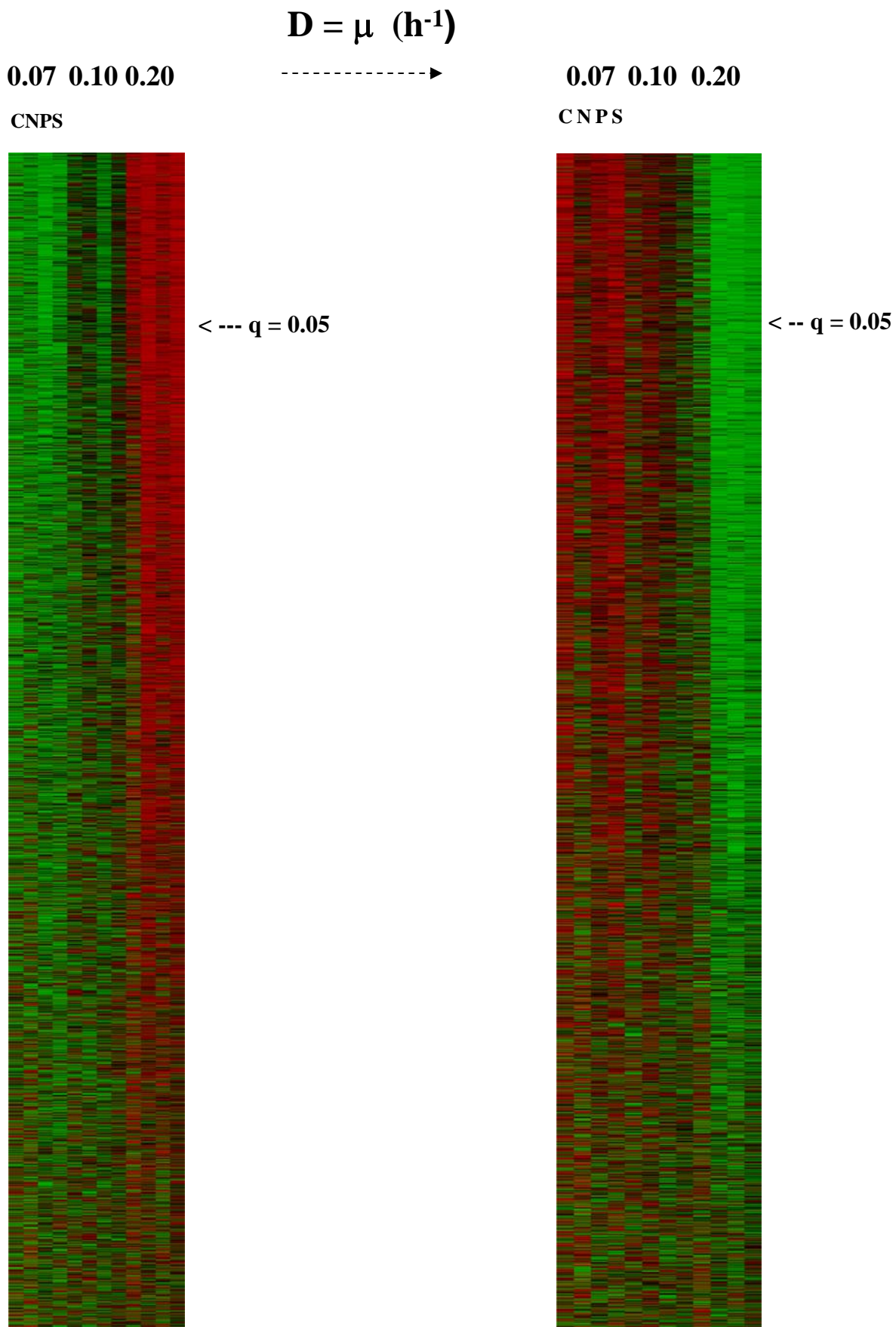

**Fig. S3**

Corresponding author: S. G. Oliver

Supplement: Additional data file 1 — Supplementary figures S1-S28. [file jbiol54-S1.zip › Fig S3 SGOliver.pdf]

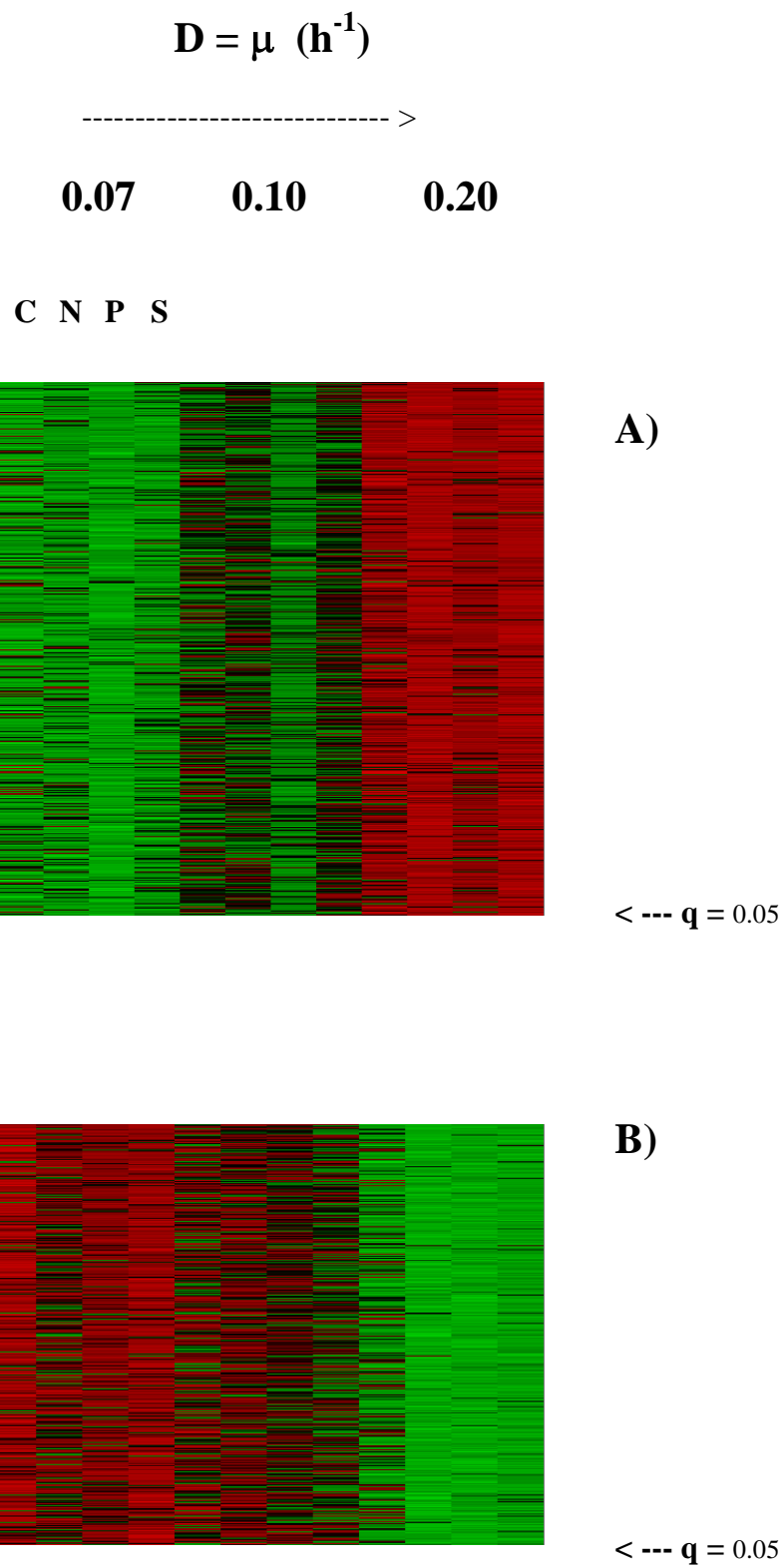

**Fig. S4.** Corresponding author: S. G. Oliver

Supplement: Additional data file 1 — Supplementary figures S1-S28. [file jbiol54-S1.zip › Fig S4 SGOliver.pdf]

# Biological process: up-regulated with growth rate

Fig. S5

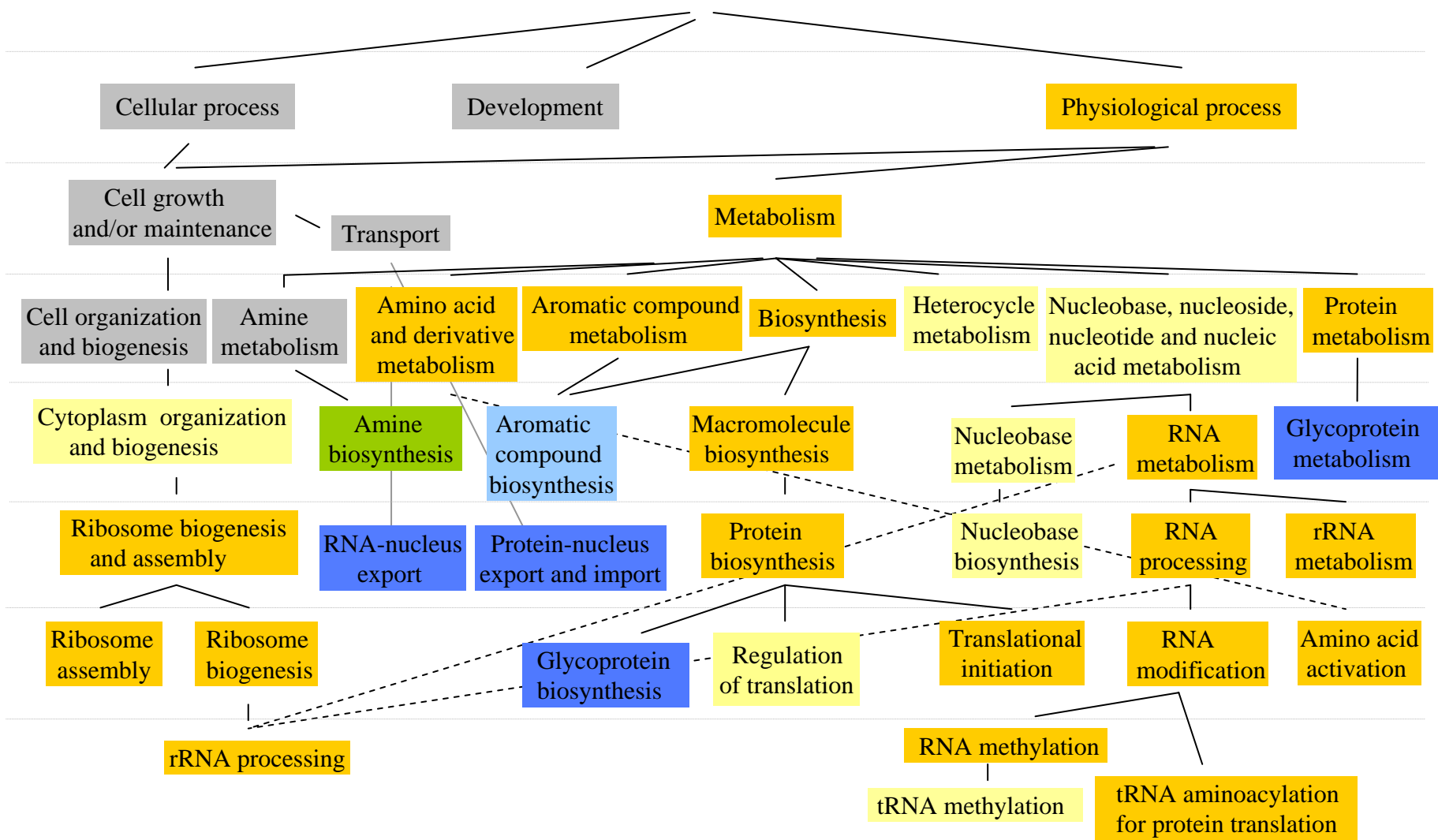

p value:

$\leq 2e^{-4}$

$2e^{-4}$  to  $4e^{-3}$

$4e^{-3}$  to  $7.5e^{-3}$

$7.5e^{-3}$  to  $1e^{-2}$

$> 1e^{-2}$

Supplement: Additional data file 1 — Supplementary figures S1-S28. [file jbiol54-S1.zip › Fig S5 SGOliver.pdf]

## Molecular function: up-regulated with growth rate

Fig. S6

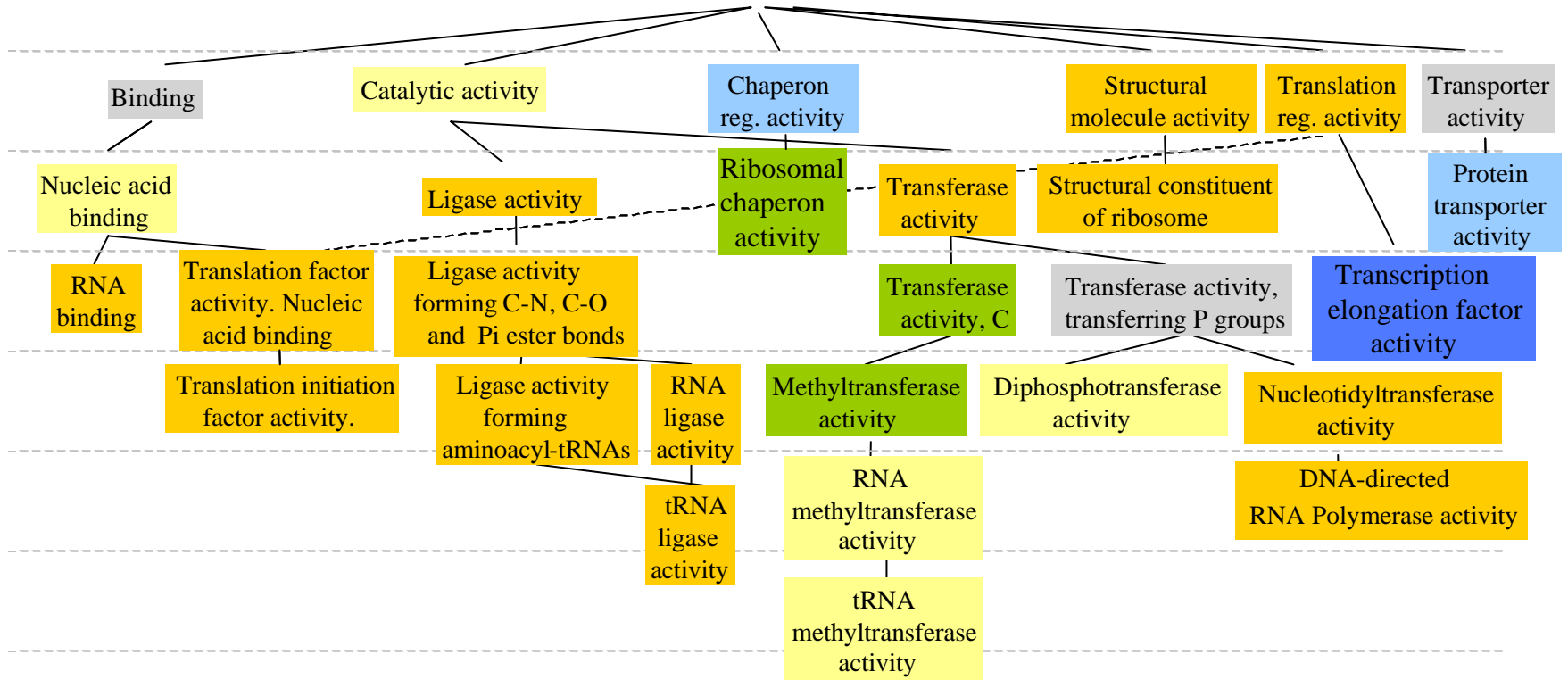

p value:

$\leq 1e^{-3}$

$1e^{-3}$  to  $1e^{-2}$

$1e^{-2}$  to  $2.5e^{-2}$

$2.5e^{-2}$  to  $5e^{-2}$

$> 5e^{-2}$

Supplement: Additional data file 1 — Supplementary figures S1-S28. [file jbiol54-S1.zip › Fig S6 SGOliver.pdf]

# Cellular component: up-regulated with growth rate

Fig. S7

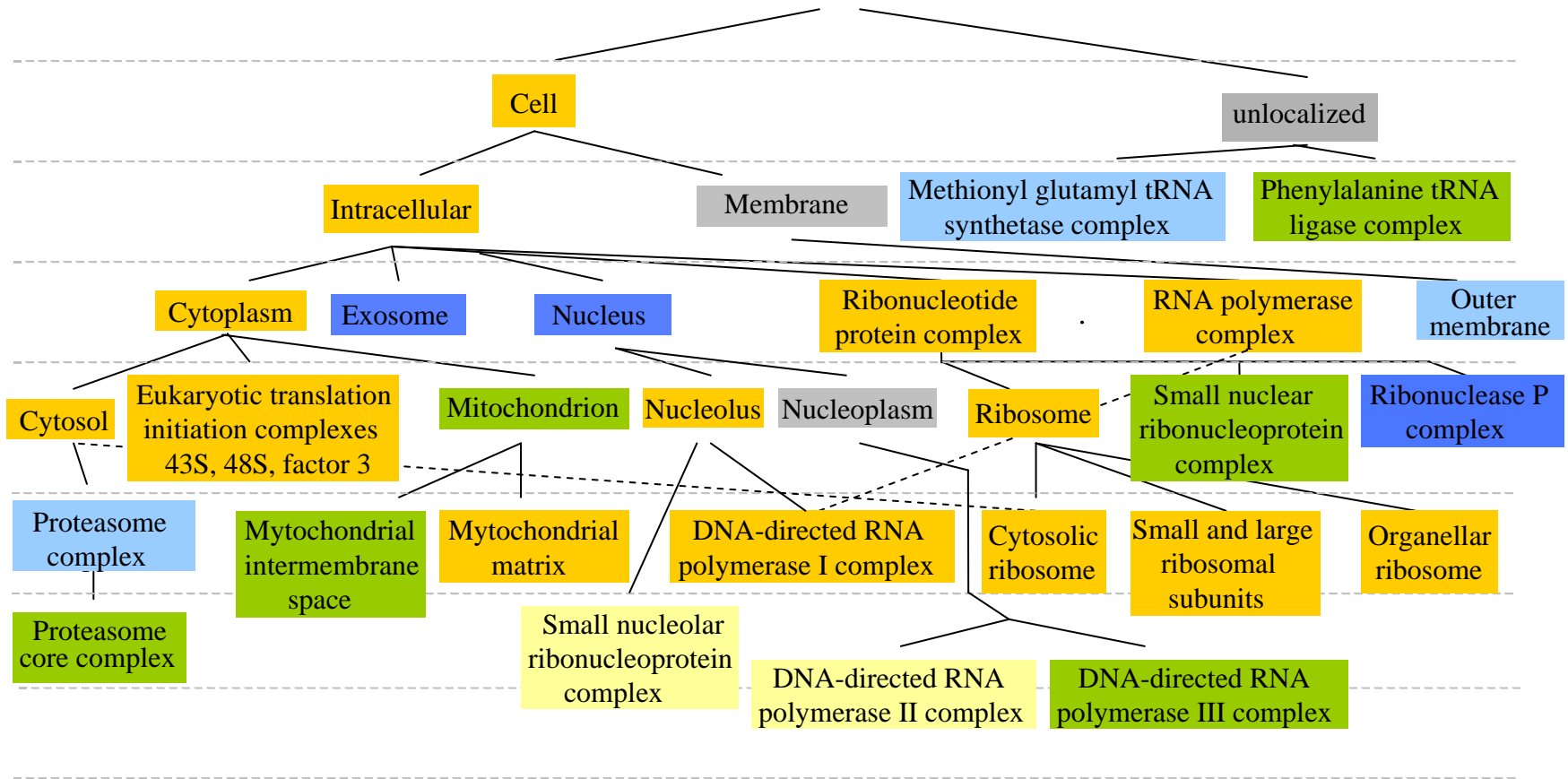

p value:

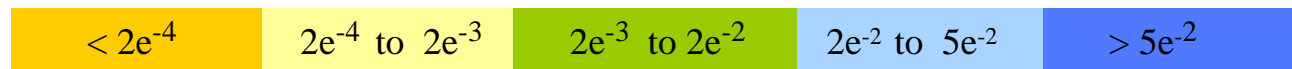

Supplement: Additional data file 1 — Supplementary figures S1-S28. [file jbiol54-S1.zip › Fig S7 SGOliver.pdf]

# Molecular function: down-regulated with growth rate

Fig. S9

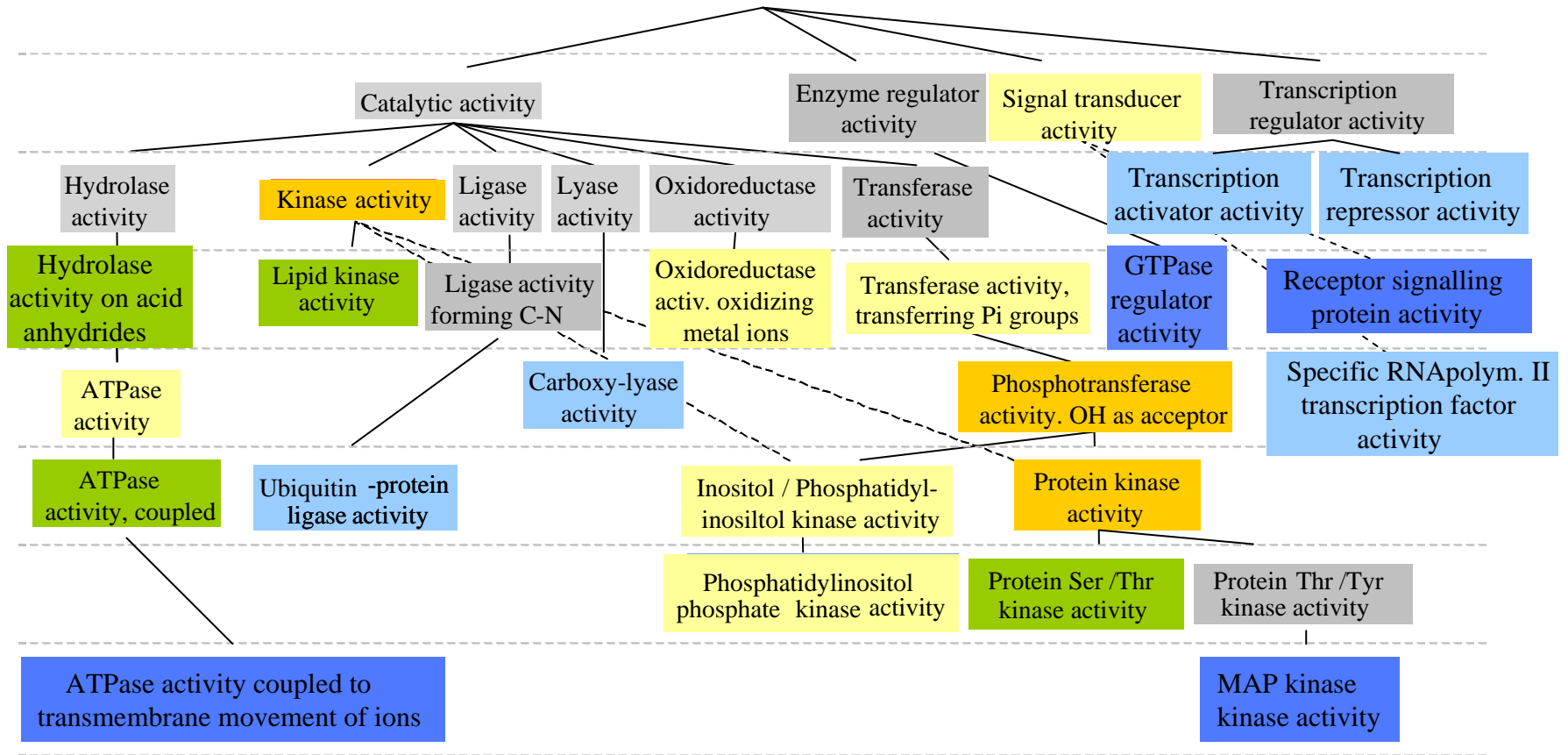

p value:

$\leq 1e^{-3}$

$1e^{-3}$  to  $1e^{-2}$

$1e^{-2}$  to  $2.5e^{-2}$

$2.5e^{-2}$  to  $5e^{-2}$

$> 5e^{-2}$

Supplement: Additional data file 1 — Supplementary figures S1-S28. [file jbiol54-S1.zip › Fig S9 SGOliver.pdf]
