# Supplementary material for: Growth control of the eukaryote cell: a systems biology study in yeast
Source: J Biol. 2007 Apr 30;6(2):4. doi: 10.1186/jbiol54 (PMC2373899; doi:10.1186/jbiol54)
Supplement: Additional data file 2 — Supplementary tables S1-S30. [file jbiol54-S2.zip › Table S30 SGOliver.pdf]

Variable	Set Parameter
Carrier Gas	Helium
Carrier gas flow rate (ml.min^{-1})	1
Injection Mode	Split
Inlet temperature (°C)	270
Sample volume injected (µl)	1
Split ratio	1 in 3
Initial GC temperature (°C)	70
Start temp hold time (min)	2
Ramp rate (°C.min^{-1})	24
Final temp (°C)	280
Hold final temp (min)	5
Mass spectrometer acquisition rate (Hz)	20
Transfer line temperature (°C)	250
Source temperature (°C)	230
Mass Range (Da)	30-500

Table S30

Corresponding author: S. G. Oliver

Table S30. GC-TOF-MS instrumental conditions.
