# Supplementary material for: Growth control of the eukaryote cell: a systems biology study in yeast
Source: J Biol. 2007 Apr 30;6(2):4. doi: 10.1186/jbiol54 (PMC2373899; doi:10.1186/jbiol54)
Supplement: Additional data file 2 — Supplementary tables S1-S30. [file jbiol54-S2.zip › Table S8 SGOliver.pdf]

Gene / ORF	q value	Viability / Mutant phenotype
CDC33 / YOL139C (eIF4E)	0.04	E. / g. d.
GCD1 / YOR260W	0.04	E. / g. d.
GCD2 / YGR083C	0.04	E. / g. d.
GCD11 / YER025W	0.04	E. / g. d.
SUI3 / YPL237W	0.03	E. / g. d.
TIF3 / YPR163C	0.04	N. E.
TIF11 / YMR260C	0.04	E. / g. d.
TIF34 / YMR146C	0.05	E.
TIF35 / YDR429C	0.04	E. / g. d.
TIF4631 / YGR162W	0.04	N. E. / g. d.
TIF5 / YPR041W	0.04	E. / g. d.
NIP1 / YMR309C	0.04	E. / g. d.
PRT1 / YOR361C	0.04	E. / g. d.
RPG1 / YBR079C	0.02	E. / g. d.

Table S8

Corresponding author: S. G. Oliver

Table S8. **Growth-rate-regulated significance (q value; obtained by ANCOVA analysis) of transcription of genes involved in translational initiation (formation and regulation of the eIF4E-cap complex), and mutant phenotypes from corresponding systematic deletion mutants.** Nomenclature: *CDC33* (or eIF4E), cytoplasmic mRNA cap binding protein; *TIF* genes, Translation Initiation Factors; *GCD* genes, General Control Derepressed translation initiation factors; *SUI3*, beta subunit of the translation initiation factor eIF2, involved in the identification of the start codon; *NIP1*, translation initiation factor eIF3 subunit; *PRT1* (or *CDC63*), subunit of the core complex of translation initiation factor 3 (eIF3); *RPG1*, subunit of the core complex of translation initiation factor 3 (eIF3). Abbreviations: N. E., non-essential gene; E, essential gene; g. d., gene disruption resulting in either inviable or growth defect phenotype in functional genomics systematic deletion studies under different conditions (Affymetrix annotation, July 12th, 2006; SGD [5]).
