# Supplementary material for: Growth control of the eukaryote cell: a systems biology study in yeast
Source: J Biol. 2007 Apr 30;6(2):4. doi: 10.1186/jbiol54 (PMC2373899; doi:10.1186/jbiol54)
Supplement: Additional data file 3 — Legends to supplementary figures and tables [file jbiol54-S3.pdf]

Additional document 3

Legends

- **Supplementary Figures.**
- **Supplementary Tables.**

Supplementary references to legends

Supplementary Figures. Legends

Fig. S1. Transcriptional studies. Gene expression signatures at the transcriptional level. Genome-wide pattern of relative changes (fold change) in transcriptional levels from growth rate (μ) 0.1 to 0.2 h^{-1} (doubling time, Td = 6.9 to 3.5 h). Carbon limitation. ORFs sorted alphabetically by biological process (Affymetrix annotation, July 12th, 2006). Up-regulation (red). Down-regulation (green).

Fig. S2. Transcriptional studies. Gene expression signatures at the transcriptional level. Genome-wide pattern of relative changes in transcriptional levels (fold change) from growth rate (μ) 0.1 to 0.2 h^{-1} (doubling time, Td = 6.9 to 3.5 h). Nitrogen limitation. ORFs sorted alphabetically by biological process (Affymetrix annotation, July 12th, 2006). Up-regulation (red). Down-regulation (green).

Fig. S3. Colour plots of transcriptional gene expression data (so-called 'Eisen' plots) [1]. Coordinate modulation of transcriptional gene expression with growth rate (~4000 genes) obtained by ANalysis of COVAriance (ANCOVA). Colour code: up-regulated genes (red); down-regulated genes (green). Gene names of significantly up- and down-regulated ORFs are included in Tables S3 and S4.

Fig. S4. Colour plots of transcriptional gene expression data (so-called 'Eisen' plots) [1]. **Up-regulated and down-regulated genes at the transcriptional level.** Groups/subsets of genes characterized by a clear, definite up- or down-regulated expression pattern with increasing growth rates, independent of the culture conditions tested, obtained by ANalysis of COVAriance (ANCOVA) (see Methods). A) Top 493 up-regulated genes with increasing growth rates (cutoff, q = 0.05). B) Top 398 down-regulated genes with increasing growth rates (up-regulated with decreasing growth rates) (cutoff, q = 0.05). Colour code: up-regulation (red); down-regulation (green) (see Methods). Gene names of significantly up- and down-regulated ORFs are included in Tables S3 and S4.

Fig. S5. Gene ontology (GO) diagram. Biological processes up-regulated with growth rate. Significantly over-represented biological processes of genes up-regulated with increasing growth rates. Results obtained from GoMiner and validated with GenMAPP (p-value corresponding to GoMiner analysis). Colour code as in *Saccharomyces* Genome Database GO Term Finder tool [2]. For complete GoMiner interactive Directed Acyclic Graphs (DAG) see Fig. S11.

Fig. S6. Gene ontology (GO) diagram. Molecular functions up-regulated with growth rate. Significantly over-represented molecular functions of genes up-regulated with increasing growth rates. Results obtained from GoMiner and validated with GenMAPP (p-value corresponding to GoMiner analysis). Colour code as in *Saccharomyces* Genome Database GO Term Finder tool [2]. For complete GoMiner interactive Directed Acyclic Graphs (DAG), see Fig. S12.

Fig. S7. Gene ontology (GO) diagram. Cellular components up-regulated with growth rate. Significantly overrepresented cellular component gene ontologies (GO) of genes up-regulated with increasing growth rates. Results obtained from GoMiner and validated with GenMAPP (p-value corresponding to GoMiner analysis). Colour code as in *Saccharomyces* Genome Database GO Term Finder tool [2]. For complete GoMiner interactive Directed Acyclic Graphs (DAG), see Fig. S13.

Fig. S8. Gene ontology (GO) diagram. Biological processes down-regulated with growth rate. Significantly overrepresented biological processes of genes down-regulated with increasing growth rates. Results obtained from GoMiner and validated with GenMAPP (p-value corresponding to GoMiner analysis). Colour code as in *Saccharomyces* Genome Database GO Term Finder tool [2]. For complete GoMiner interactive Directed Acyclic Graphs (DAG), see Fig. S14.

Fig. S9. Gene ontology (GO) diagram. Molecular functions down-regulated with growth rate. Significantly overrepresented molecular functions of genes down-regulated with increasing growth rates. Results obtained from GoMiner and validated with GenMAPP (p-value corresponding to GoMiner analysis). Colour code as in *Saccharomyces* Genome Database GO Term Finder tool [2]. For complete GoMiner interactive Directed Acyclic Graphs (DAG), see Fig. S15.

Fig. S10. Gene ontology (GO) diagram. Cellular components down-regulated with growth rate. Significantly overrepresented cellular component gene ontologies (GO) of genes down-regulated with increasing growth rates. Results obtained from GoMiner and validated with GenMAPP (p-value corresponding to GoMiner analysis). Colour code as in *Saccharomyces* Genome Database GO Term Finder tool [2]. For complete GoMiner interactive Directed Acyclic Graphs (DAG), see Fig. S16.

Fig. S11 to S16. Directed Acyclic Graphs (DAG files)

Fig. S11. Complete gene ontology (GO) diagram. Biological processes up-regulated with growth rate. Complete interactive GO tree (DAG diagram) of overrepresented biological processes corresponding to genes significantly up-regulated with increasing growth rate. GO tool: GoMiner. Colour code: overrepresented gene ontologies (red); underrepresented gene ontologies (blue).

Fig. S12. Complete gene ontology (GO) diagram. Molecular functions up-regulated with growth rate. Complete interactive GO tree (DAG diagram) of overrepresented molecular functions corresponding to genes significantly up-regulated with increasing growth rate. GO tool: GoMiner. Colour code as in Fig. S11.

Fig. S13. Complete gene ontology (GO) diagram. Cellular components up-regulated with growth rate. Complete interactive GO tree (DAG diagram) of overrepresented cellular component gene ontologies corresponding to genes significantly up-regulated with increasing growth rate. GO tool: GoMiner. Colour code as in Fig. S11.

Fig. S14. Complete gene ontology (GO) diagram. Biological processes down-regulated with growth rate. Complete interactive GO tree (DAG diagram) of overrepresented biological processes corresponding to genes significantly down-regulated with increasing growth rate. GO tool: GoMiner. Colour code as in Fig. S11.

Fig. S15. Complete gene ontology (GO) diagram. Molecular functions down-regulated with growth rate. Complete interactive GO tree (DAG diagram) of overrepresented molecular functions corresponding to genes significantly down-regulated with increasing growth rate. GO tool: GoMiner. Colour code as in Fig. S11.

Fig. S16. Complete gene ontology (GO) diagram. Cellular components down-regulated with growth rate. Complete interactive GO tree (DAG diagram) of overrepresented cellular component gene ontologies corresponding to genes significantly down-regulated with increasing growth rate. GO tool: GoMiner. Colour code as in Fig. S11.

Fig. S17. Ribosomal biogenesis and assembly genes. Groups of genes significantly up-regulated and down-regulated with increasing growth rates corresponding to the biological process: Ribosomal biogenesis and assembly (GO tool: GenMAPP). Colour code: Red: Genes up-regulated with increasing growth rate (i.e. down-regulated with decreasing growth rate); Green: Genes down-regulated with increasing growth rate (i.e. up-regulated with decreasing growth rate). Bright colours: Most significantly regulated genes ($q < 0.05$), included in the top up- (493) or top down- regulated (398) lists obtained by ANCOVA analysis. Dark/pale colours: Groups of up- and down-regulated genes in the range ($0.05 < q < 0.10$).

Fig. S18. RNA polymerase complex. Groups of genes significantly up-regulated and down-regulated with increasing growth rates, corresponding to the RNA polymerase complex (GO tool: GenMAPP). Colour code as in Fig. S17.

Fig. S19. tRNA aminoacylation for protein translation. Groups of genes significantly up-regulated and down-regulated with increasing growth rates, corresponding to the biological process: tRNA aminoacylation for protein translation (GO tool: GenMAPP). Colour code as in Fig. S17.

Fig. S20. Storage vacuole. Groups of genes significantly up-regulated and down-regulated corresponding to the cellular component: Storage vacuole (GO tool: GenMAPP). Colour code as in Fig. S17.

Fig. S21. Autophagy. Groups of genes significantly up-regulated and down-regulated corresponding to the biological process: Autophagy (GO tool: GenMAPP). Colour code as in Fig. S17.

Fig. S22. Schematic diagram of the target of rapamycin (TOR) pathway regulating temporal control of cell growth (TORC1 complex) in yeast. Most significantly up-regulated (bright red) and down-regulated (bright green) genes with increasing growth rate ($q < 0.05$). Dark/pale colours: Groups of genes in the range ($0.05 < q < 0.10$). GO tool: GenMAPP. Adapted from Loewith and Hall (2004) [3], Reinke *et al.,* (2004) [4] and *Saccharomyces* genome database (SGD) [5]. Symbols. (+) Existence of orthologous proteins in five eukaryotic organisms (*Ashbya gossypii, Caenorhabditis elegans, Arabidopsis thaliana, Drosophila melanogaster and Homo sapiens*). (*) Existence of orthologous proteins in *Homo sapiens* (SGD Model Organism BLASTP Best Hits and Ensembl database) [6-9].

Fig. S23. TOR regulation of gene expression at the transcriptional level. Role of the TOR pathway (TORC1 complex) in coordinate up- and down-regulation of growth-related genes at the transcriptional level (temporal control of cell growth [3])

Fig. S24. Transcriptional patterns of gene expression of TORC1 components. Variations in expression levels with increasing growth rates of the components of the TORC1 complex, for the different chemostat experiments (i.e. nutrient-limiting conditions and growth rates). Standardised gene expression levels (for each gene the standard deviation was set to one and mean set to zero for logarithmic data obtained by the RMA method).

Fig. S25. TOR1 and TOR2 transcriptional patterns of gene expression. Variations in transcriptional levels with increasing growth rate of TOR1 and TOR2 compared to the top up-regulated gene ribosomal protein gene (RPL18B). Standardised gene expression levels (for each gene the standard deviation was set to one and mean set to zero for logarithmic data obtained by the RMA method).

Fig. S26. Proteomic studies. Gene expression signatures at the protein level. Patterns of relative changes (fold change) in protein levels from growth rate (μ) 0.1 to 0.2 h^{-1} (doubling time, Td = 6.9 to 3.5h). Nitrogen limitation (663 proteins). ORFs sorted by biological process (*Saccharomyces* Genome Database, SGD) [5]. Up-regulation (red). Down-regulation (green).

Fig. S27. Relative changes in translational control efficiency. Translational control. Patterns of relative changes in translational control efficiencies from growth rate (μ) 0.1 to 0.2 h^{-1}. Nitrogen limitation. ORFs sorted by biological process (*Saccharomyces* Genome Database, SGD) [5]. Up-regulation (red). Down-regulation (green).

Fig. S28. Proteomics experimental strategy. Experiments and iTRAQ labelling. 114.1 tag, equal amount (w/w) of proteins extracted from all samples (pooled standard, P). Experiment 1: 115.1 tag, N limited (0.1h^{-1}); 116.1 tag, C limited (0.1 h^{-1}); 117.1 tag, S limited (0.1h^{-1}); Experiment 2: 115.1 tag, P limited (0.1h^{-1}); 116.1 tag, N limited (0.2 h^{-1}); 117.1 tag, C limited (0.2h^{-1}); Experiment 3: 115.1 tag, S limited (0.2h^{-1}); 116.1 tag, P limited (0.2 h^{-1}); 117.1 tag = equal amount (w/w) of proteins extracted from all samples (pooled standard, P).

Supplementary Tables. Legends

Table S1. Relative changes in transcriptional gene expression. Carbon limitation. Genome-wide patterns of relative changes (fold change) in transcriptional levels ($\mu = 0.1\ h^{-1}$ to $0.2\ h^{-1}$).

Table S2. Relative changes in transcriptional gene expression. Nitrogen limitation. Genome-wide patterns of relative changes (fold change) in transcriptional levels ($\mu = 0.1\ h^{-1}$ to $0.2\ h^{-1}$).

Table S3. Top up-regulated genes with increasing growth rates. List of 493 top up-regulated genes, consistently up-regulated with increasing growth rate irrespective of nutrient limitation conditions, obtained by ANCOVA analysis ranked by q value, including: a) Essential genes and mutant phenotypes from corresponding systematic deletion studies; b) Protein conserved in human and five eukaryotic model organisms; c) Human orthologous. Abbreviations: g. d., gene disruption resulting in either inviable or growth defect phenotype. g. i., gene interactions resulting in either inviable phenotype or slow growth (Affymetrix annotation, July 12th, 2006; SGD [5]; SGD Model Organism BLASTP Best Hits [6, 7]; Ensembl database [8, 9]). Low abundance transcripts detected under this study are marked with asterisks (*) (see methods). The list includes nineteen ORFs for which more than one probe set is present in the Affymetrix chip (marked R, R’, R’’).

Table S4. Top down-regulated genes with increasing growth rate. List of 398 top down-regulated genes, consistently down-regulated with increasing growth rate irrespective of nutrient limitation conditions, obtained by ANCOVA analysis ranked by q value, including: a) Essential genes and mutant phenotypes from corresponding systematic deletion studies; b) Protein conserved in human and five eukaryotic model organisms; c) Human orthologous. Abbreviations: g. d., gene disruption resulting in either inviable or growth defect phenotype. g. i., gene interactions resulting in either inviable phenotype or slow growth (Affymetrix annotation, July 12th, 2006; SGD [5]; SGD Model Organism BLASTP Best Hits [6, 7]; Ensembl database [8, 9]). Low abundance transcripts detected under this study are marked with asterisks (*) (see methods). The list includes six ORFs for which more than one probe set is present in the Affymetrix chip (marked R, R').

Table S5. Significantly overrepresented biological processes of genes up-regulated with increasing growth rate. Table ranked by p value obtained from GoMiner. Results validated with GenMAPP.

Table S6. Significantly overrepresented molecular functions of genes up-regulated with increasing growth rate. Table ranked by p value obtained from GoMiner. Results validated with GenMAPP.

Table S7. Significantly overrepresented cellular component gene ontologies of genes up-regulated with increasing growth rate. Cellular component. Table ranked by p value obtained from GoMiner. Results validated with GenMAPP.

Table S8. **Growth-rate-regulated significance (q value; obtained by ANCOVA analysis) of transcription of genes involved in translational initiation (formation and regulation of the eIF4E-cap complex), and mutant phenotypes from corresponding systematic deletion mutants.** Nomenclature: *CDC33* (or eIF4E), cytoplasmic mRNA cap binding protein; *TIF* genes, Translation Initiation Factors; *GCD* genes, General Control Derepressed translation initiation factors; *SUI3*, beta subunit of the translation initiation factor eIF2, involved in the identification of the start codon; *NIP1*, translation initiation factor eIF3 subunit; *PRT1* (or *CDC63*), subunit of the core complex of translation initiation factor 3 (eIF3); *RPG1*, subunit of the core complex of translation initiation factor 3 (eIF3). Abbreviations: N. E., non-essential gene; E, essential gene; g. d., gene disruption resulting in either inviable or growth defect phenotype in functional genomics systematic deletion studies under different conditions (Affymetrix annotation, July 12th, 2006; SGD [5]).

Table S9. Significantly overrepresented biological processes of genes down-regulated with increasing growth rate (up-regulated with decreasing growth rates). Table ranked by p value obtained from GoMiner. Results validated with GenMAPP.

Table S10. Significantly overrepresented molecular functions of genes down-regulated with increasing growth rate (up-regulated with decreasing growth rates). Table ranked by p value obtained from GoMiner. Results validated with GenMAPP.

Table S11. Significantly overrepresented cellular component gene ontologies of genes down-regulated with increasing growth rate (up-regulated with decreasing growth rates). Table ranked by p value obtained from GoMiner. Results validated with GenMAPP.

Table S12. Protein-protein interactions in list of genes up-regulated with growth rate. (see Additional Document 4. Supplementary methods)

Table S13. Protein-protein interactions in list of genes down-regulated with growth rate. (see Additional Document 4. Supplementary methods)

Table S14. Protein-protein interactions between up- and down-regulated lists of genes. (see Additional Document 4. Supplementary methods)

Table S15. Up-regulated genes with growth rate and down-regulated by rapamycin. 397 up-regulated genes controlled by TOR significantly down-regulated by rapamycin treatment (see methods).

Table S16. Down-regulated genes with growth rate and up-regulated by rapamycin. 249 down-regulated genes controlled by TOR significantly up-regulated by rapamycin treatment (see methods).

Table S17. Proteasome subunits significantly up-regulated at the transcriptional level by rapamycin treatment.

Table S18. Relative changes in protein levels (proteomic signatures). Carbon limitation. Patterns of relative changes (fold change) in protein levels (μ = 0.1 h^{-1} to 0.2 h^{-1}).

Table S19. Biological processes corresponding to proteins significantly up-regulated from growth rate 0.1 to 0.2 h^{-1}. Carbon limitation. Proteins significantly up-regulated with increasing growth rate (relative fold changes higher than 20%, 141 proteins) from iTRAQ studies were analysed by SGD GO Term Finder, [2]).

Table S20. Biological processes corresponding to proteins significantly down-regulated from growth rate 0.1 to 0.2 h^{-1}. Carbon limitation. Proteins significantly down-regulated with increasing growth rate (relative fold changes higher than 20%, 160 proteins) from iTRAQ studies were analysed by SGD GO Term Finder, [2]).

Table S21. Relative changes in protein levels (proteomic signatures). Nitrogen limitation. Patterns of relative changes (fold change) in protein levels (μ = 0.1 h^{-1} to 0.2 h^{-1}).

Table S22. Proteins consistently up-regulated at the protein level with increasing growth rate. List of top up-regulated proteins, consistently up-regulated with increasing growth rate irrespective of nutrient limitation conditions, obtained by ANOVA analysis (see methods).

Table S23. Proteins consistently down-regulated at the protein level with increasing growth rate. List of top down-regulated proteins, consistently down-regulated with increasing growth rate irrespective of nutrient limitation conditions, obtained by ANOVA analysis (see methods).

Table S24. Relevant enzymes consistently up- and down-regulated at the protein level responsible of control of metabolic fluxes in central growth-related metabolic pathways.

Table S25. Relevant transcripts with translational efficiencies consistently up- and down-regulated with growth rate.

Table S26. Metabolome studies. Glutamate, glutamine and arginine biosynthetic pathway. Relative changes in intracellular metabolic levels of relevant metabolites in the glutamate, glutamine and arginine biosynthetic pathway from growth rate (μ) 0.1 to 0.2 h^{-1} (doubling time, Td = 6.9 to 3.5h).

Table S27. Metabolic control at the protein (enzyme) level: Glutamate, glutamine and arginine biosynthetic pathway. Relative changes in enzyme levels within the glutamate, glutamine and arginine biosynthetic pathway from growth rate (μ) 0.1 to 0.2 h^{-1} (doubling time, Td = 6.9 to 3.5h) . Enzymes consistently up-regulated are marked in red.

Table S28. Metabolome studies. Methionine/methyl cycle pathway. Relative changes in intracellular metabolic levels of relevant metabolites in the methionine/methyl cycle pathway from growth rate (μ) 0.1 to 0.2 h^{-1} (doubling time, Td = 6.9 to 3.5h).

Table S29. Metabolic control at the protein (enzyme) level: Sulphur, C1 (folate), methionine and methyl cycle pathways. Protein relative changes from growth rate (μ) 0.1 to 0.2 h^{-1} (doubling time, Td = 6.9 to 3.5h). Enzymes consistently up- or down- regulated are marked in red and green respectively.

Table S30. GC-TOF-MS instrumental conditions.

Supplementary references to legends

1 Eisen MB, Spellman PT, Brown PO, Botstein D: **Cluster analysis and display of genome-wide expression patterns.** *Proc Natl Acad Sci U S A* 1998, **95**:14863-14868.

2 ***Saccharomyces*** **Genome Database GO Term Finder tool**. [http://db.yeastgenome.org/cgi-bin/GO/goTermFinder]

3 Loewith R, Hall MN: **TOR signalling in yeast: Temporal and spatial control of cell growth.** In *Cell Growth. Control of Cell Size*. Monograph 42. Hall MN, Raff M and Thomas G. New York: Cold Spring Harbor Laboratory Press; 2004:139-165.

4 Reinke A, Anderson S, McCaffery JM, Yates J 3rd, Aronova S, Chu S, Fairclough S, Iverson C, Wedaman KP, Powers T: **TOR complex 1 includes a novel component, Tco89p (YPL180w), and cooperates with Ssd1p to maintain cellular integrity in *Saccharomyces cerevisiae*.** *J Biol Chem* 2004, **279**:14752-14762.

5 ***Saccharomyces*** **genome database (SGD)** [http://www.yeastgenome.org]

6 Balakrishnan R, Christie KR, Costanzo MC, Dolinski K, Dwight SS, Engel SR, Fisk DG, Hirschman JE, Hong EL, Nash R *et al.*: **Fungal BLAST and Model Organism BLASTP Best Hits: new comparison resources at the *Saccharomyces* Genome Database (SGD)**. *Nucleic Acids Res* 2005, **33:**Database Issue: D374-377.

7 **SGD Model Organism BLASTP Best Hits** [http://db.yeastgenome.org/cgi-bin/bestHits]

8 Birney E, Andrews TD, Bevan P, Caccamo M, Chen Y, Clarke L, Coates G, Cuff J, Curwen V, Cutts T *et al.*,: **An overview of Ensembl.** *Genome Res* 2004, **14**: 925-928.

9 **Ensembl database** [http://www.ensembl.org]
