# Supplementary material for: Growth control of the eukaryote cell: a systems biology study in yeast
Source: J Biol. 2007 Apr 30;6(2):4. doi: 10.1186/jbiol54 (PMC2373899; doi:10.1186/jbiol54)
Supplement: Additional data file 4 — Supplementary methods. [file jbiol54-S4.pdf]

Additional document 4

Supplementary Methods

Supplementary Methods

Cultivation conditions

Yeast strains were grown in fermenters, essentially as described before [1]. A set of chemostat cultures under C-, N-, P- and S-limiting conditions were established at a series of dilution rates (0.07, 0.10, 0.20 h^{-1}). Cultures were fed with a defined mineral medium limiting growth by carbon, nitrogen, phosphorus, or sulphur [1], all other nutritional requirements were in excess at a constant residual concentration. Glucose and vitamins were added to the medium after separate sterilization. Glucose was heat-sterilized at 120ºC. Vitamins were prepared and sterilized by filtration through 0.2 μm filters (Sartopore®2, Sartorius, UK). Chemostat cultivation was performed in 2-litre fermenters (FT Applikon Ltd., UK) with a working volume of 1.0 litre, a temperature of 30ºC and a stirrer speed of 750 rpm. The culture pH was maintained at 5.0 by automatic addition of 2 M NaOH or HCl as required, via an Applikon ADI-1030 biocontroller. Aerobic cultivation was performed by maintaining an air flow of 1.0 litre min^{-1}. The dissolved oxygen concentration in all chemostat cultures, as measured with an Ingold polarographic oxygen electrode, was above 50% of saturation. To avoid loss of volatile metabolites, the off-gas condenser was cooled to 4ºC, connected to a cryostat. Off-gas oxygen and carbon dioxide levels were monitored with a Tandem gas analyser (Magellan Instruments, UK). Steady state conditions were deemed established once biomass levels (absorbance and dry weight measurements), dissolved oxygen, and off-gas fractions remained constant over three residence times (three volume changes) [2]. For the rapamycin study, a batch culture growing at mid-exponential phase in minimal medium [3] was established under the same cultivation conditions as for chemostat experiments. This was divided into two flasks. Rapamycin (200 ng ml^{-1}) was added to one half, and the vehicle added to the other, as the control. Samples were taken at 0, 1, 2 and 4h after the treatment.

On-line and off-line measurements

Dissolved oxygen, oxygen and carbon dioxide gas levels were monitored on-line. At appropriate intervals, culture samples were collected for determination of biomass concentration. Biomass levels were monitored both spectrophotometrically at a wavelength of 600 nm, and by dry weight. Dry weight measurements were determined as described by Postma *et al.* (1989) [4]. The purity of the cultures was routinely checked by phase-contrast microscopy and by plating on selective media.

Transcriptional studies. Labelling, hybridisation and scanning protocols. Data analysis.

Total RNA was extracted from biomass as previously described [5]. Microarray experiments were performed using Affymetrix Yeast YG_S98 GeneChip oligonucleotide arrays (Affymetrix, Inc. USA) as described [6], according to the manufacturer's instructions [7]. Briefly, 15 μg of total RNA was used to prepare first strand cDNA using an oligo(dT)-T7 primer. Following second strand synthesis, biotinylated cRNA targets were generated using the Enzo Bio Array High Yield RNA Transcript Labelling Kit (Affymetrix, Inc. USA) by *in vitro* transcription with biotinylated UTP and CTP. The fragmented cRNA targets together with labelled controls were hybridized to the arrays at 45ºC, rotating at 60 rpm for 16 h according to the manufacturer's instructions. Following hybridization, the arrays were processed using Affymetrix fluidics protocol "EukGE-WS2", and stained with R-phycoerythrin conjugated to streptavidin (Molecular Probes, Inc. USA). Images of the arrays were acquired using the Microarray suit (MAS) v5.0 software and an Affymetrix 2500 GeneChip scanner. For each of 12 conditions (four nutritional limitations at three dilution rates) four arrays were carried out. Robust Multichip Average (RMA) quantile normalization [8, 9] and further analysis were carried out using RMAExpress software [10]. YG_S98 Affy probes marked as 'Gene1///Gene2' in the Gene Symbol column from Affymetrix annotation,

correspond to probe sets which are insufficiently discriminatory to resolve between both genes (Affymetrix personal communication). Low-abundance transcripts for the culture conditions tested (C, N, P, and S-limitation) were obtained by calculating the mean expression level for each condition and finding those probe sets whose expression level was below 100 (6.644 in $\log_2$) under all conditions (see Tables S3 and S4). In compliance with MIAME guidelines [11], the data from these studies have been submitted to the ArrayExpress repository [12] at the European Bioinformatics Institute (EBI) under accession numbers E-MEXP-115 and E-MAXD-4 (for the rapamycin studies).

Data analysis and processing for Principal Components Analysis (PCA)

The objective was to measure the response to growth and nutrient limitation at several 'omic levels in order to discover clustering patterns and trends and, in so doing, understand how the 'cell system' responds as a whole. Principal Components Analysis (PCA) [13] is suited to this purpose because it is an unsupervised multivariate approach that considers all the variables at once and then performs dimensional reduction giving greatest weight to variables that change the most.

Exploratory cluster analysis using Principal Components Analysis (PCA) was then performed on the transcriptional and proteome data and metabolic profiles. Since transcriptome, proteome and metabolome data needed to be gathered by different methodologies, data analysis procedures were required to minimize systematic bias imposed by differences in measurement techniques, and to be consistent in data processing prior to PCA. Table 1 summarizes the data analysis steps performed to handle each class of 'omic data, some of which are explained below. We have sought to be consistent throughout, for example logarithmic transformations were applied to all data types. On the other hand, particular data types required treatments that others did not (e.g. microarray data required no

missing value imputation whereas other data types did). The integration of data sets from the different levels of 'omic analysis will be a recurrent theme in both Functional Genomics and Systems Biology. Standard approaches need to be developed and, whilst these are beyond the scope of this contribution, we will publish a separate paper to discuss these problems.

Briefly, the transcriptome data were subjected to a background adjustment, RMA quantile normalization and summarization using RMAExpress software [10], and centring MAD (Median Absolute Deviation) normalization [14]. For the proteomics PCA analysis, after each run was normalized to pooled standard (Fig. S28), sample-wise MAD normalization [14], missing-value imputation (MVI) using Bayesian missing value estimation method [15] and measurement-wise MAD normalization [14] were performed. For the metabolome data, metabolite samples were normalized to internal standard. Missing-value imputation (MVI) [15] and measurement-wise MAD normalization [14] were applied (Table 1). PCA was performed with Matlab® Version 6.5 [16] running under Windows XP on an IBM-compatible PC, and the PCA implemented using the NIPALS algorithm [17]. The results obtained from these analyses were then visualized using Spotfire®DecisionSite™ 7.3 version 11.0 [18].

Transcriptome and proteome signatures, and changes in metabolite levels

To facilitate comparison of transcriptome and proteome patterns, growth rates 0.1 h^{-1} and 0.2 h^{-1} were compared. For each nutrient limitation, the relative change in expression was analysed between the growth rates. In the iTRAQ proteome studies, since there was no replication this was simply calculated as the log and natural relative expression. Prior to this the logged data were normalized measurement-wise per run to the pooled standard and then normalized sample-wise by MAD, scaling to average MAD. For transcriptome studies a modified t-test, Cyber-T [19, 20] with p-value and false discovery rate (q-value) calculation

[21, 22] was applied on the RMA-normalized data. Relative metabolite levels (0.2 vs 0.1 h^{-1}) were obtained by normalization of each metabolite peak area to the internal standard**.**

Transcriptional studies. Global gene expression analysis. ANOVA/ANCOVA model.

The normalized logged intensities were analysed to identify probes from those genes whose expression level was regulated predominantly by growth rate (μ), irrespective of the identity of the growth-limiting nutrient, (l). This suggested an Analysis Of Variance (ANOVA) of the normalized logged intensities should be performed [23, 24].

In principle, normalization should have removed a number of main and interaction effects that are independent of specific probes, i.e. global biases. Therefore, we considered all remaining effects to be probe-dependent and performed the ANOVA on a probe-by-probe basis. Denoting the logged intensity for the lth medium limitation, r^{th} rate and k^{th} replicate by y_{lrk} , we have the ANOVA model:

$y_{lrk} = \mu + L_l + R_r + LR_{lr} + \varepsilon_{lrk}$,   (1)

with $\Sigma_l L_l = 0$, $\Sigma_r R_r = 0$, $\Sigma_l LR_{lr} = \Sigma_r LR_{lr} = 0$,

$E(\varepsilon_{lrk}) = 0$, $E(\varepsilon_{lrk}^2) = \sigma^2$.

The significances of the main effects and interaction term are determined by calculating their appropriate F test statistic [23]. Following Kerr *et. al.* (2000) [24], p-values were estimated by re-sampling from suitably re-scaled residuals [25] under a homoskedastic assumption that the same value for σ^2 was appropriate for each probe. We fitted the ANOVA model in (1) to 10^6 re-sampled data sets. The effect of multiple testing was accounted for by estimating the false discovery rate (FDR) using the q-value method and R-code of Storey [21, 22], for which default settings were used. For the main effects and interaction term, a q-value was calculated for each probe. The growth rate is a continuous-valued parameter that denotes the local specific change in biomass concentration in the controlled culture. The

ANOVA coefficients R_r and LR_{lr} represent the limitation-independent and limitation-specific effect of growth rate on transcriptional gene expression, respectively. Both the limitation-independent and limitation-specific effects will contain a linear component, proportional to growth rate, and a non-linear component. From a biological perspective, we might expect the response to growth rate of transcriptional gene expression to be predominantly proportional, with participation of a second smaller non-linear component. Therefore, we decided to treat the growth rate quantitatively and identify the contribution to the total variance of the linear response component, equivalent to performing an Analysis of Covariance (ANCOVA), even though r takes on a finite set of values and is considered to be controlled without error. The main growth rate effect and interaction terms can be decomposed into a linear and non-linear component,

$R_r = a_0\Delta r + \delta R_r, \quad LR_{lr} = a_l\Delta_r + \delta LR_{lr}$

$\Delta r = r - \bar{r}, \quad \Sigma_l a_l = 0,$

$\Sigma_r\delta R_r = 0, \ \Sigma_r\Delta r\delta R_r = 0, \ \Sigma_r\delta LR_{lr} = 0, \ \Sigma_l\delta LR_{lr} = 0, \ \Sigma_r\Delta r\delta LR_{lr} = 0,$

where $\bar{r}$ = (0.07+0.1+0.2)/3 is the average of the growth rates studied. Of those probes (4307/9335) for which the main effect due to rate in the ANOVA model was significant ($q < 0.01$) only 2.9% showed a significant ($q < 0.01$) non-linear effect. Of those probes (1746/9335) for which the interaction term in (1) was significant ($q<0.01$), 32.9% had a significant non-linear contribution. This suggests that the large majority of probes show a predominantly linear response to the effect of rate. We therefore decided to use the linear model as a basis of further filtering probes for subsequent analysis. a_0 represents the, limitation-independent, transcriptional response to growth rate. A large positive value of a_0 indicates a substantial element of up-regulation of transcriptional gene expression with increasing growth rate. Correspondingly, a large negative value of a_0 indicates a substantial limitation-independent element of down-regulation of gene expression with increasing

growth rate. The notion of a probe (gene) being growth-rate-regulated was interpreted as meaning that the main effect of rate, a_0, was dominant over any interaction between growth rate and the type of limiting medium. Thus, for each probe, the ratio of the proportion of the total variance explained by the main rate effect, a_0, to the proportion of the total variance explained by the interaction term was calculated. This is equivalent to calculating the ratio $M^2 = a_0^2 / ¼\Sigma_l a_l^2$. This has the intuitive interpretation that a large value of M indicates that rate largely has the same general effect irrespective of the type of limited medium. Probes representing growth-rate-regulated ORFs are then represented by statistically significant values of a_0 and correspondingly large values of M. These were selected by constructing a conservative p-value estimate as the proportion of re-sampled data sets in which either the observed value of M or the observed F-statistic for a_0 was exceeded. Associated q-values were calculated in the same manner as before, and probes filtered by applying a cut-off to the q-value.

Global data analysis of transcriptional studies gene lists

From the ANCOVA results, normalized, filtered data (focussing on genes of known function; ‘Eisen’ plots [26] (Fig. S3 and S4) and tables of significantly up- and down-regulated genes on the basis of q were obtained. The ‘Eisen’ plots were created with maxdView (available from [27]). The data for each gene in log. base 2 were normalized by z transformation (mean set to 0 and standard deviation 1). To facilitate visual comparison, the colours were set to a linear ramp of green for expression below the mean, through black for no change from the mean, to red for expression above the mean.

Clusters/groups of probesets (genes) judged to be most significantly up- or down-regulated with growth rate were formed based on a rank of our ANCOVA false discovery rate q (see above). We sought two clusters of well-annotated genes for subsequent analysis. The dataset

was therefore filtered for probesets with "Gene Symbol" annotation in the most recent Affymetrix annotation update (July 12th, 2006). To enable robust gene ontology analysis q cut-offs were selected and fixed based on q = 0.05 (5%) for up- and down-regulated genes. Gene ontology analyses (GoMiner, GenMAPP, see below) focussed mainly on most significantly growth-regulated genes (q < 0.05), with GenMAPP analyses extending the study including significantly growth-regulated genes in the range (0.05 < q <0.10) (see results).

For the rapamycin study, all the probesets on the array were subjected to a two-way ANOVA analysis (time and rapamycin factors) using GeneSpring® software (Agilent Technologies, Santa Clara, CA) [28]. The p-values arising from this analysis were subjected to a false discovery rate estimation using default settings of the Q-value software [22]. Probesets that had a q-value less than 0.05 for the rapamycin and interaction factors were regarded as significantly affected by rapamycin treatment and used in further analysis. For the elucidation of groups of over- or under-represented genes, a standard binomial test was used [29].

Proteomics ANOVA

The MAD-normalized proteomics log.-ratios (ratio to pooled standard, Fig. S28) were taken as the starting point for a similar analysis to that performed with the transcriptome data, with the aim of identifying proteins whose abundances were significantly growth-rate-regulated. An identical analysis of the transcriptome data was not possible since no technical replicate measurements were available and only two growth rates were studied, $\mu = 0.1h^{-1}$ and $0.2h^{-1}$.

To attempt to identify growth-rate regulated proteins the normalized proteomics values were decomposed, on a protein by protein basis, using a two-factor ANOVA model containing only main terms,

$$y_{rl} = \mu + R_r + L_l + \varepsilon_{rl} \qquad (2)$$

with $\Sigma_r R_r = 0$, $\Sigma_l L_l = 0$ and $E(\varepsilon_{rl}) = 0$, $E(\varepsilon_{rl}^2) = \sigma^2$

where y_{rl} represents the normalized log-ratio for the rth rate and l^{th} limitation.

Growth-rate regulated proteins were identified as those for which the inclusion of the growth-rate main term R_r was statistically significant. This corresponds to a statistically significant value for the contrast $R_{0.2} - R_{0.1}$, thereby indicating a statistically significant change in protein abundance just due to growth rate. As for the analysis of the transcriptome data, p-values for the main terms in (2), were estimated from 10^6 bootstrap data sets constructed by re-sampling from suitably re-scaled estimates of the residuals ε_{lr}. To correct for multiple testing the Q-value software of Storey [21, 22] was used to estimate the False Discovery Rate (FDR).

Integrative proteome-transcriptome analysis. Proteome-transcriptome correlations.

Integration of proteome and transcriptome data towards proteome-transcriptome correlation studies and analysis of relative changes in translational control efficiencies required prior stringent data analysis, use of specific normalization and scaling of ‘omic data, leading to equivalent, comparable data sets. Thus, transcriptome data were subjected to RMA normalization [8-10], whereas proteome data were MAD normalized [14] and then scaled to the average median absolute deviation (to make it as comparable as possible to RMA). For proteome-transcriptome correlations, graphs in log. and natural values are presented. For studies on relative changes in translational control efficiencies, data sets and graphs are in natural values only (graphs and ratios log [(/) p] $_i$ / log [(/) tr.] $_i$ have no direct relation with relative changes in translational control efficiencies; see Additional data file 7, page 7).

Gene ontology (GO) studies

For gene ontology (GO) studies, a number of GO tools [30] were tested, from which GoMiner [31, 32] and GenMAPP [33, 34] were selected, on the basis of their statistical analysis capabilities and appropriate visualization tools, to display the significance of over-represented gene ontologies (biological process, molecular function and cellular component). Analyses using GoMiner were thoroughly validated with GenMAPP, and corresponding interactive Directed Acyclic Graphs (DAG) (see below), GO maps and tables with selected groups of genes were obtained. For studies not covering the whole genome (e.g. proteomic studies; ca. 700 proteins per nutrient-limiting condition) SGD GO Term Finder [35] was used.

Directed Acyclic Graphs (DAG)

DAG graphs were created in SVG, -Scalable Vector Graphics-, format (SVG Viewer available free from [36]). Once installed, the DAG files can be opened with Internet Explorer. SVG works best with Internet Explorer (IE) 5.0, IE 5.5, IE 6, and Netscape 4.7 on Windows; and Netscape 4.7 Mac. Use right click to zoom in/out and (Alt + mouse) to drag/centre the diagram. Passing above a node shows the GO ontology and genes involved. Double-clicking on a node shows all existent relationships of that node in the global gene ontology network.

Analysis of protein-protein interactions

Protein interaction data was extracted from the latest BioGRID database of curated protein-protein interactions (BioGRID database, [37, 38], including data from Reguly *et al.*, (2006) [39] and new 866 physical and genetic interactions, as of May 1, 2006). Only curated physical interactions were used for the analysis. All non-physical interactions (interactions

where the method of detection was marked up as one of the following: synthetic lethality, dosage rescue, synthetic growth defect, synthetic rescue, epistatic miniarray profile, dosage lethality, phenotypic enhancement, phenotypic suppression or dosage growth defect) and self-self interactions were excluded.

Numbers of protein interactions expected by chance were calculated by selecting an equivalent number of ORFs at random from the selection of ORFs present within BioGRID. Interactions between the ORFs in the random set were then extracted from the known protein interaction network and the number of interactions within the set was then calculated. P values were calculated by comparing the observed value to 10,000 random permutations, being (in all cases) in the range of $p<1E^{-4}$.

References

1 Baganz F, Hayes A, Marren D, Gardner DCJ, Oliver SG: **Suitability of replacement markers for functional analysis studies in *Saccharomyces cerevisiae*.** *Yeast* 1997, **13**:1563-1573.

2 Stafford K: **Continuous fermentation.** In *Manual of Industrial Microbiology and Biotechnology*. Edited by Demain AL and Solomon NA. Washington: American Society for Microbiology; 1986:137-151.

3 Burke D, Dawson D, Stearns T: *Methods in Yeast Genetics*. New York: Cold Spring Harbour Laboratory Press; 2000.

4 Postma E, Verduyn C, Scheffers WA, van Dijken JP: **Enzymic analysis of the Crabtree effect in glucose-limited chemostat cultures of *Saccharomyces cerevisiae*.** *Appl Environ Microbiol* 1989, **55**:468-477.

5 Hayes A, Zhang N, Wu J, Butler PR, Hauser NC, Hoheisel JD, Lim FL, Sharrocks AD, Oliver SG: **Hybridization array technology coupled with chemostat culture: Tools to interrogate gene expression in *Saccharomyces cerevisiae*.** *Methods* 2002, **26**:281-290.

6 Wishart JA, Hayes A, Wardleworth L, Zhang N, Oliver SG: **Doxycycline, the drug used to control the *tet*-regulatable promoter system has no effect on global gene expression in *Saccharomyces cerevisiae***. *Yeast* 2005, **22**:565-569.

7 **Affymetrix website** [http://www.affymetrix.com]

8 Bolstad BM, Irizarry RA, Astrand M, Speed, TP: **A comparison of normalization methods for high density oligonucleotide array data based on variance and bias.** *Bioinformatics* 2003, **19**:185-193.

9 Irizarry RA, Bolstad BM, Collin F, Cope LM, Hobbs B, Speed TP: **Summaries of Affymetrix GeneChip probe level data.** *Nucleic Acids Res* 2003, **31:**e15.

10 **RMAExpress software** [http://rmaexpress.bmbolstad.com/]

11 **MIAME website** [http://www.mged.org/Workgroups/MIAME/miame.html]

12 **ArrayExpress public repository** [http://www.ebi.ac.uk/arrayexpress/]

13 Joliffe IT: *Principal components analysis*. New York: Springer-Verlag; 1986.

14 Yang YH, Dudoit S, Luu P, Lin DM, Peng V, Ngai J, Speed TP: **Normalization for cDNA microarray data: a robust composite method addressing single and multiple slide systematic variation.** *Nucleic Acids Res* 2002, **30**:e15.

15 Oba S, Sato MA, Takemasa I, Monden M, Matsubara K, Ishii S: **A Bayesian missing value estimation method for gene expression profile data.** *Bioinformatics* 2003, **19**:2088-2096.

16 **Matlab® software** [http://www.mathworks.com]

17 Wold H: **Estimation of Principal Components and Related Models by Iterative Least Squares**. In *Multivariate Analysis*. Edited by Krishnaiah PR. New York: Academic Press; 1966:391-420.

18 **Spotfire® software** [http://www.spotfire.com]

19 Baldi P, Long AD: **A Bayesian framework for the analysis of microarray expression data: regularized t-test and statistical inferences of gene changes.** *Bioinformatics* 2001, **17**:509-519.

20 **Cyber-T program** [http://visitor.ics.uci.edu/genex/cybert/]

21 Storey JD: **A direct approach to false discovery rates.** *J. Royal Stat. Soc. series B* 2002, **64**:479-498.

22 **q-value software** [http://faculty.washington.edu/~jstorey/qvalue/]

23 Scheffé H: *The Analysis of Variance.* New York: John Wiley & Sons; 1959.

24 Kerr MK, Martin M, Churchill GA: **Analysis of variance for gene expression microarray data**. *J of Comput Biol* 2000, **7**: 819-837.

25 Wu CFJ: **Jackknife, bootstrap and other resampling methods in regression analysis.** *Ann Statist* 1986, **14**:1261-1295.

26 Eisen MB, Spellman PT, Brown PO, Botstein D: **Cluster analysis and display of genome-wide expression patterns.** *Proc Natl Acad Sci U S A* 1998, **95**:14863-14868.

27 **maxdView software** [http://bioinf.man.ac.uk/microarray/maxd/]

28 **GeneSpring® software** [http://www.chem.agilent.com/]

29 **SISA binomial test statistical tool** [http://home.clara.net/sisa/binomial.htm]

30 **Gene ontology (GO) tools website** [http://www.geneontology.org/GO.tools.html]

31 **GoMiner tool** [http://discover.nci.nih.gov/gominer/]

32 Zeeberg BR, Feng W, Wang G, Wang MD, Fojo AT, Sunshine M, Narasimhan S, Kane DW, Reinhold WC, Lababidi S *et al.,*: **GoMiner: a resource for biological interpretation of genomic and proteomic data.** *Genome Biol* 2003, **4**:R28.

33 **GenMAPP tool** [http://www.genmapp.org/]

34 Dahlquist KD, Salomonis N, Vranizan K, Lawlor SC, Conklin, BR: **GenMAPP, a new tool for viewing and analyzing microarray data on biological pathways.** *Nat Genet* 2002, **31**:19-20.

35 ***Saccharomyces* Genome Database (SGD)** [http://www.yeastgenome.org]

36 **Scalable Vector Graphics (SVG Viewer) software** [http://www.adobe.com/svg/]

37 **BioGRID database** [http://www.thebiogrid.org]

38 Stark C, Breitkreutz BJ, Reguly T, Boucher L, Breitkreutz A, Tyers M: **BIOGRID: a general repository for interaction datasets.** *Nucleic Acids Res* 2006, **34** (Database issue):D535-D539.

39 Reguly T, Breitkreutz A, Boucher L, Breitkreutz BJ, Hon GC, Myers CL, Parsons A, Friesen H, Oughtred R, Tong A *et al.* **Comprehensive curation and analysis of global interaction networks in *Saccharomyces cerevisiae*.** *J Biol* 2006, **5**:11.

40 **Genome Annotating Proteomic Pipeline (GAPP)** [http://www.gapp.info]

Steps	Data analysis procedures	Transcriptome	Proteome	Internal metabolites	External metabolites
1	Analytical method	Affymetrix YG-S98 GeneChip	iTRAQ	GC-TOF-MS	GC-TOF-MS
	Growth rates (h^{-1})	0.07, 0.1, 0.2	0.1, 0.2	0.07, 0.1, 0.2	0.07, 0.1, 0.2
	Nutrient limitations	C, N, P, S	C, N, P, S	C, N, P, S	C, N, P, S
	Replication	4	1	3	3
2	Identification and quantification	Affymetrix GCOS software [7]	Genome Annotating Proteomic Pipeline (GAPP) system [40], Mascot score and link to the quantification data in a relational database (see methods).	Mass spectral libraries, reverse match score >700 , and analysis of pure standards (see methods)	Mass spectral libraries, reverse match score >700 , and analysis of pure standards (see methods)
3	Heavily-tailed distribution	Data logged	Data logged	Data logged	Data logged
4	Experiment-wise normalization	---	Each run normalized to pooled standard (Fig. S28)	---	---
5	Sample-wise normalization	RMA normalization [8-10]	MAD (median absolute deviation) [14]	Normalized to internal standard	Normalized to internal standard
6	Missing value imputation (MVI)	Not required	MVI [15]	MVI [15]	MVI [15]
7	Measurement-wise normalization	MAD [14]	MAD [14]	MAD [14]	MAD [14]
8	Principal Components Analysis (PCA)	NIPALS [17]	NIPALS [17]	NIPALS [17]	NIPALS [17]

Table 1. Data analysis procedures prior to PCA
