# Supplementary material for: Growth control of the eukaryote cell: a systems biology study in yeast
Source: J Biol. 2007 Apr 30;6(2):4. doi: 10.1186/jbiol54 (PMC2373899; doi:10.1186/jbiol54)
Supplement: Additional data file 5 — Additional studies on transcriptional control of cell growth. [file jbiol54-S5.pdf]

Additional document 5. Transcriptional control of cell growth

Gene ontologies and processes regulating cell growth at the transcriptional level

Genes up-regulated with increasing growth rate

The initiation of translation (i.e., formation and regulation of the eIF4E-cap complex) is tightly regulated during growth [1-3] and probably acts to integrate cell growth and cell division [4]. Thus, dysregulation of cap-dependent translation has been reported to confer malignant characteristics and to induce cancer [5, 6]. Our finding that all yeast genes involved in translational initiation are amongst those whose transcription is consistently and significantly up-regulated with increasing growth rate (Table S8) confirms the critical role of this process in the overall control of growth, with a relevant participation of post-transcriptional and post-translational mechanisms as well (i.e. association of the mRNA cap binding protein eIF4E with 4E-binding proteins, controlled by phosphorylation-dephosphorylation) [2-4, 7]. The integration of growth and division via translation initiation (at least, in response to nutrient supply) is thought to be exerted mainly through the rapamycin-sensitive TOR signal transduction pathway (TORC1 complex) [1, 2].

Ribosome biogenesis and assembly require a huge investment of the cell's energetic and material resources. Moreover, all ribosomal components have to be synthesised co-ordinately in exact stoichiometric proportions in order to sustain balanced growth [8-10]. The fact that many ribosomal protein genes can act as haploinsufficient tumour suppressors in zebrafish [11] reflects the importance of coordinate stoichiometric regulation of the ribosome biogenesis and assembly machinery in balanced growth [9, 10, 12]. Growth-rate dependent regulation of ribosomal-protein synthesis at the transcriptional level has been reported for batch cultures of yeast and

other organisms grown on different carbon sources [9, 13-15] although, translational controls have also be shown to be involved [10]. Our results (Figs. S5 and S17) confirm that ribosome biogenesis and assembly are central growth-related processes, in which a large number of genes are significantly up-regulated with increasing growth rates, irrespective of the growth-limiting nutrient (Fig. S17). Ribosome biogenesis has been reported to be regulated through the cAMP-dependent protein kinase (PKA) of the RAS/cAMP signal transduction pathway – which is, itself, subject to control by the TOR-signalling pathway [2, 10, 12, 16].

RNA polymerases are central to the control of growth via the regulation of mRNA synthesis, as well as being responsible for the RNA components of the translational apparatus, rRNAs and tRNAs. RNA polymerase III (pol III) occupies a unique position, in that it is responsible for the synthesis of two components of the translational machinery (tRNAs and 5S rRNA), as well as 7SL RNA. A wide range of transformed and tumour cell types have been shown to express elevated levels of pol III products [17]. The exact mechanisms involved in regulating the expression of genes encoding components of the three RNA polymerase complexes are the subject of intensive study (White, 2004b [18] and references therein). Our data show a clear coordinated up-regulation of the transcription of these genes with increasing growth rate (Fig. S18). Aminoacyl-tRNA synthetases are responsible for the charging of tRNAs with amino acids to sustain protein synthesis. Our results show that a majority of the 20 genes encoding these enzymes are up-regulated with increasing growth-rates (Fig. S19). This is also confirmed by proteomics data which showed that the levels of the aminoacyl-tRNA synthetases were also consistently up-regulated at the level of protein synthesis with increasing growth rates (see proteomic studies).

The proteasome is a supramolecular protein complex dedicated mainly to controlled proteolysis of ubiquitin conjugated proteins [19, 20], although new roles and participation in mechanisms of control of gene expression have also been reported (e.g. altering the SAGA coactivator and its interactions with transcriptional activators) [21]. It's a highly complex ATPase-dependent protease consisting of over 30 subunits, the majority of which are essential (SGD database; [22]). Subunit expression, protein composition and levels of individual proteolytic activities have been reported to vary under different growth states [23], which points to a relevant role of this complex in balanced growth, not always considered or being overlooked in the majority of cases. In our transcriptional studies eight specific protein subunits (mainly 20S proteasome subunits) were found consistently up-regulated with growth rate (Table S3) which points to a coordinate regulation of protein synthesis and degradation during balanced growth.

Genes down-regulated with increasing growth rate

Cell growth entails the coordinated up- and down-regulation of a variety of processes. Genes that are down-regulated with increasing growth rate are probably involved in maximising the efficient utilisation of cellular resources at each different growth rate and culture condition, particularly when nutrients are scarce. Our data indicate that this is a poorly understood aspect of the cell's economy since a significant number of these genes (140/398) are of as yet undetermined function (Fig. 2). This is despite the fact that nutrient scarcity is likely to be a common circumstance in the organism's natural environment [24]. Among the genes of known function that are up-regulated at low growth rates are those involved in mobilisation and storage of available resources at the level of the vacuole (Fig. S20). Another interesting example

of genes that are up-regulated at low growth rates are those involved in autophagy (Fig. S21). Autophagy is a major system of bulk degradation of cellular components. It participates in the coordinate degradation of cytoplasmic components, including proteins, large complexes and organelles whose turnover is important in the control of cell growth. Autophagy mediates the shrinkage of the ribosome pool, thus slowing cell growth when nutrients are limiting [25]. Autophagy in yeast has been reported to be a TOR-mediated response to nutrient starvation [26], and we have demonstrated previously the induction of autophagy genes in stationary phase [27]. Autophagy genes are well conserved from yeast to mammals, suggesting that it is a fundamental activity of eukaryotic cells, being implicated in processes such as homeostasis, development and differentiation [25]. Other genes that are up-regulated at low growth rates are those encoding specific transcriptional repressors whose action results in the activation of alternative routes for the assimilation of substrates and/or as an adaptation to the environment.

In all, the data on the down-regulated genes present a picture of the yeast cell at low growth rates, activating pathways involved in the response to external stimuli, maintenance of homeostasis, vacuolar transport and storage, and autophagy; the whole being directed towards a more efficient use of scarce resources.

Protein-protein interactions

Remarkably, whereas proteins encoded by the up-regulated genes participate in a large number of interactions with each other (876; expected by chance 287), the ones encoded by the down-regulated genes rarely interact with one another (89; expected by chance 193) (Tables S12 and S13). The existence of a high number of interactions in the first list can be explained since the up-regulated genes include many protein

complexes and components of the translational machinery. The existence of a low number of interactions in the group of down-regulated genes points to a more relevant contribution of other mechanisms, with participation of kinases, phosphotransferases, ATPases and oxidoreductases among others (Table S10). Moreover, an important number of pair-interactions were encountered between the up- and down-regulated lists (233; predicted by chance 472) (Table S14), which points to the existence of direct, close interrelationships between the two sets.

Coordination of cell growth and division. Transcriptional studies

The most accepted, generally occurring model is that one in which cell growth precedes cell division [4] where the cell must be able to monitor its actual 'cell size', either as mass, volume or biosynthetic fluxes. The present perspective points to the existence of two different mechanisms: (1) Mechanisms controlling the actual cell size, and (2) Mechanisms controlling the critical size, maximum threshold attainable by the cell under each specific condition before being committed to cell division at 'Start' [10, 28-31]. As far as the control of actual cell size is concerned, Jorgensen and coworkers (2002) [28] tested the entire yeast gene knockout set for genes affecting cell size, finding 451 candidate genes (221 small mutants and 230 large mutants) with translation initiation factors, ribosomal protein genes, aminoacyl-tRNA synthetases, RNA pol II transcription complexes and proteasome subunits among the most relevant families and functional categories underlying cell size control, in good agreement with our results, particularly on genes and functions transcriptionally up-regulated with increasing growth rates (Fig S5 and S6; Table S6). We found a group of 94 genes with mutants affected in cell size significantly growth-rate regulated (76 up-regulated genes, 18 in the down regulated set) (Tables S3 and S4), whereas other

groups of genes with mutants affected in cell size do not appear in our lists. The global results show good congruence and agree with the present perspective that control of actual cell size is complex and can be exerted by different mechanisms at different regulatory levels (i.e. transcriptional and post-transcriptional level), with the role of many individual genes still to be elucidated [30].

As far as the control of the critical cell size, same relevant study by Jorgensen and coworkers (2002) [28] resulted in the discovery of a 'regulon' of 29 genes underlying the mechanisms controlling 'critical cell size' and the coupling between growth and cell division [28, 29]. In this study, two relevant genes were found situated upstream this 'regulon', Ptk2, a reported putative serine/threonine kinase, and the transcriptional activator Sfp1 controlling 15 ribosomal protein genes [28, 29]. In our studies Ptk2 was found significantly down-regulated (Table S4) with increasing growth rates. We didn't find Sfp1 significantly regulated at the transcriptional level, which agrees with studies that refer its predominant role at the subcellular localization (nucleus:cytosolic ratio) level, as reported for other ribosomal protein genes regulators (e.g. Sch9; Ifh1, Fhl1) [32-36]. With independence of this, we could identify eight of the ribosomal protein genes controlled by Sfp1 as exhibiting significantly up-regulation with growth at the transcriptional level (Table S3). Apart from this, the initiation of translation has also been reported as a critical point which can mediate the coupling between cell growth and cell division [4], which is also regulated by TOR [3, 37]. More comprehensive studies at the transcriptional, translational, post-translational and metabolic level are necessary to advance our knowledge of the mechanisms coordinating cell growth and cell division.

References

1 Barbet NC, Schneider U, Helliwell SB, Stansfield I, Tuite MF, Hall MN: **TOR controls translation initiation and early G1 progression in yeast.** *Mol Biol Cell* 1996, **7**:25-42.

2 Hall MN, Raff M, Thomas G: *Cell Growth. Control of Cell Size*. Monograph 42. New York: Cold Spring Harbor Laboratory Press; 2004.

3 Petroulakis E, Sonenberg N: **Translation initiation and cell growth control.** In *Cell Growth. Control of Cell Size*. Monograph 42. Hall MN, Raff M and Thomas G. New York: Cold Spring Harbor Laboratory Press; 2004:299-328.

4 Schmidt EV: **Coordination of cell growth and cell division**. In *Cell Growth. Control of Cell Size*. Monograph 42. Hall MN, Raff M and Thomas G. New York: Cold Spring Harbor Laboratory Press; 2004:101-137.

5 Bjornsti MA, Houghton PJ: **The TOR pathway: a target for cancer therapy.** *Nat Rev Cancer* 2004a, **4**:335-348.

6 Bjornsti MA, Houghton PJ: **Lost in translation: dysregulation of cap-dependent translation and cancer.** *Cancer Cell* 2004b, **5**:519-523.

7 Matsuo H, Li H, McGuire AM, Fletcher CM, Gingras AC, Sonenberg N, Wagner G: **Structure of translation factor eIF4E bound to m7GDP and interaction with 4E-binding protein.** *Nat Struct Biol* 1997, **4**:717-724.

8 Warner JR: **The economics of ribosome biosynthesis in yeast.** *Trends Biochem Sci* 1999, **24**:437-440.

9 Planta RJ: **Regulation of ribosome synthesis in yeast.** *Yeast* 1997, **13**:1505-1518.

10 Jorgensen P, Tyers M, Warner JR: **Forging the factory: Ribosome synthesis and growth control in budding yeast.** In *Cell Growth. Control of Cell Size*. Monograph 42. Edited by Hall MN, Raff M and Thomas G. New York: Cold Spring Harbor Laboratory Press; 2004a:329-370.

11 Amsterdam A, Sadler KC, Lai K, Farrington S, Bronson RT, Lees JA, Hopkins N: **Many ribosomal protein genes are cancer genes in zebrafish.** *PLoS Biol* 2004, **2**:E139.

12 Powers T, Walter P: **Regulation of ribosome biogenesis by the rapamycin-sensitive TOR-signaling pathway in *Saccharomyces cerevisiae*.** *Mol. Biol. Cell* 1999, **10**:987-1000.

13 Mager WH, Planta RJ: **Coordinate expression of ribosomal protein genes in yeast as a function of cellular growth rate.** *Mol Cell Biochem* 1991, **104**:181-187.

14 Kraakman LS, Griffioen G, Zerp S, Groeneveld P, Thevelein JM, Mager WH, Planta RJ: **Growth-related expression of ribosomal protein genes in *Saccharomyces cerevisiae*.** *Mol Gen Genet* 1993, **239**:196-204.

15 Popescu SC, Tumer NE: **Silencing of ribosomal protein L3 genes in *N. tabacum* reveals coordinate expression and significant alterations in plant growth, development and ribosome biogenesis.** *Plant J* 2004, **39**:29-44.

16 Thomas G, Sabatini DM, Hall MN: *TOR. Target of rapamycin. Current Topics in Microbiology and Immunology Series. Vol. 279.* New York: Springer-Verlag; 2004.

17 White RJ: **RNA polymerase III transcription and cancer.** *Oncogene* 2004a, **23**:3208-3216.

18 White RJ: **Control of rRNA and tRNA production is closely tied to cell growth**. In *Cell Growth. Control of Cell Size*. Monograph 42. Hall MN, Raff M and Thomas G. New York: Cold Spring Harbor Laboratory Press; 2004b:371-412.

19 Zwickl P, Seemuller E, Kapelari B, Baumeister W: **The proteasome: a supramolecular assembly designed for controlled proteolysis.** *Adv Protein Chem* 2001, **59**:187-222.

20 Ciechanover A: **Proteolysis: from the lysosome to ubiquitin and the proteasome.** *Nat Rev Mol Cell Biol* 2005, **6**:79-87.

21 Lee D, Ezhkova E, Li B, Pattenden SG, Tansey WP, Workman JL: **The proteasome regulatory particle alters the SAGA coactivator to enhance its interactions with transcriptional activators.** *Cell* 2005, **123**:423-436.

22 ***Saccharomyces* Genome Database (SGD)** [http://www.yeastgenome.org]

23 Chen Q, Thorpe J, Ding Q, El-Amouri IS, Keller JN: **Proteasome synthesis and assembly are required for survival during stationary phase.** *Free Radic Biol Med* 2004, **37**:859-868.

24 Ferenci T: **Regulation by nutrient limitation.** *Curr Opin Microbiol* 1999, **2**:208-213.

25 Ohsumi Y: **Autophagy: Reversing Cell Growth.** In *Cell Growth. Control of Cell Size*. Monograph 42. Hall MN, Raff M and Thomas G. New York: Cold Spring Harbor Laboratory Press; 2004:413-429.

26 Kamada Y, Sekito T, Ohsumi Y: **Autophagy in yeast: a TOR-mediated response to nutrient starvation.** *Curr Top Microbiol Immunol* 2004, **279**:73-84.

27 Wu J, Zhang N, Hayes A, Panoutsopoulou K, Oliver SG: **Global analysis of nutrient control of gene expression in *Saccharomyces cerevisiae* during growth and starvation.** *Proc Natl Acad Sci U S A* 2004, **101**:3148-3153.

28 Jorgensen P, Nishikawa JL, Breitkreutz BJ, Tyers M: **Systematic identification of pathways that couple cell growth and division in yeast.** *Science* 2002, **297**:395-400.

29 Sudbery P: **Cell biology. When wee meets whi.** *Science* 2002, **297**:351-352.

30 Jorgensen P, Tyers M: **How cells coordinate growth and division.** *Curr Biol* 2004, **14**:R1014-R1027.

31 Jorgensen P, Rupes I, Sharom JR, Schneper L, Broach JR, Tyers M: **A dynamic transcriptional network communicates growth potential to ribosome synthesis and critical cell size.** *Genes Dev* 2004b, **18**:2491-2505.

32 Marion RM, Regev A, Segal E, Barash Y, Koller D, Friedman N, O'Shea EK: **Sfp1 is a stress- and nutrient-sensitive regulator of ribosomal protein gene expression.** *Proc Natl Acad Sci U S A* 2004, **101**:14315-1422.

33 Martin DE, Soulard A, Hall MN: **TOR regulates ribosomal protein gene expression via PKA and the Forkhead transcription factor FHL1.** *Cell* 2004, **119**:969-979.

34 Schawalder SB, Kabani M, Howald I, Choudhury U, Werner M, Shore D: **Growth-regulated recruitment of the essential yeast ribosomal protein gene activator Ifh1.** *Nature* 2004, **432**:1058-1061.

35 Wade JT, Hall DB, Struhl K: **The transcription factor Ifh1 is a key regulator of yeast ribosomal protein genes.** *Nature* 2004, **432**:1054-1058.

36 Rudra D, Zhao Y, Warner JR: **Central role of Ifh1p-Fhl1p interaction in the synthesis of yeast ribosomal proteins.** *EMBO J* 2005, **24**: 533-542.

37 Fingar DC, Blenis J: **Target of rapamycin (TOR): an integrator of nutrient and growth factor signals and coordinator of cell growth and cell cycle progression.** *Oncogene* 2004, **23**:3151-3171.
