# Supplementary material for: Growth control of the eukaryote cell: a systems biology study in yeast
Source: J Biol. 2007 Apr 30;6(2):4. doi: 10.1186/jbiol54 (PMC2373899; doi:10.1186/jbiol54)
Supplement: Additional data file 6 — Proteome-transcriptome correlations. [file jbiol54-S6.pdf]

Additional document 6

Proteome – transcriptome correlations

Proteome – transcriptome correlations
(relative changes from 0. 1 to 0.2 h^{-1})

(Log_2-Log_2 scale)

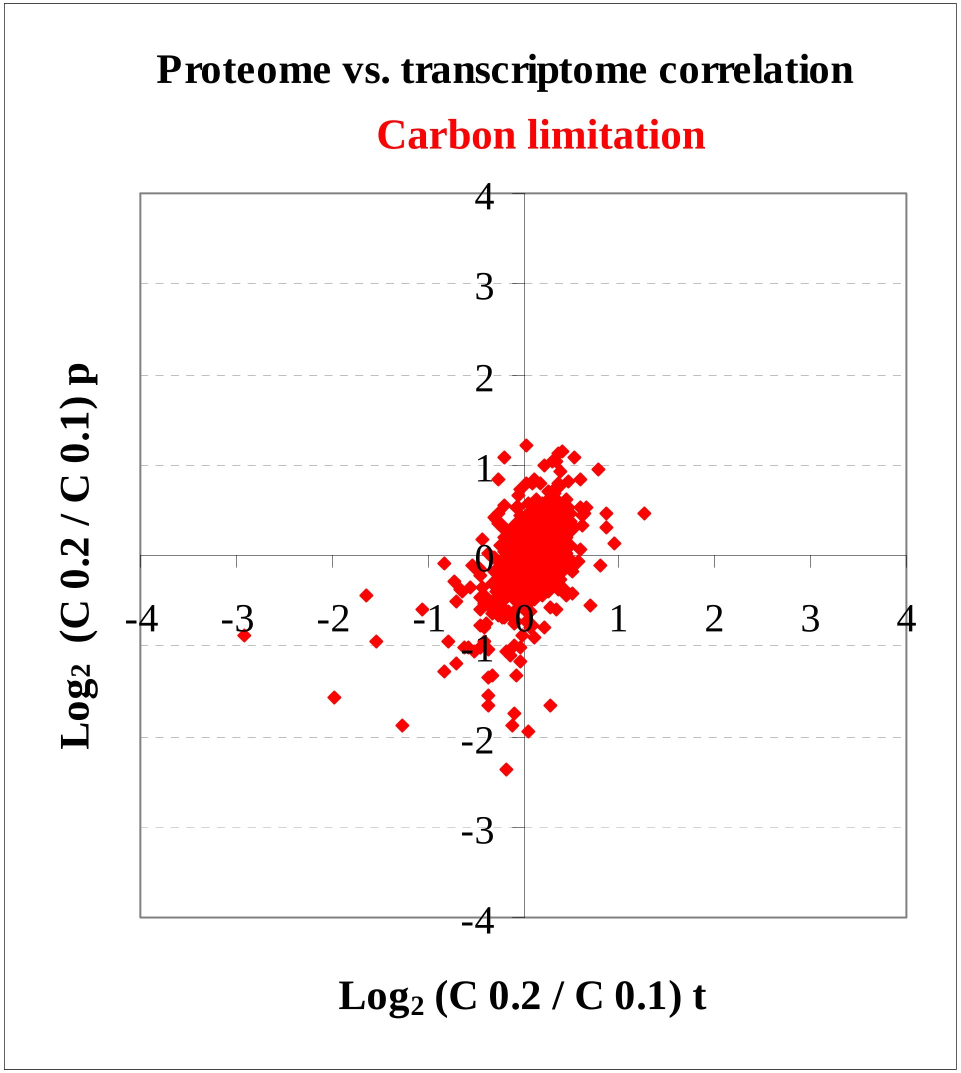

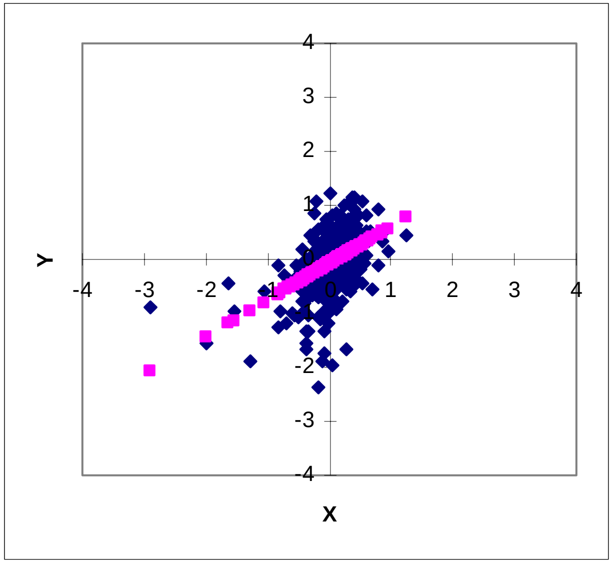

Linear Regression
Y = A + B * X

Parameter	Value	Error
A	-0.08494	0.01531
B	0.6785	0.04826

R	SD	N	P
0.47514	0.38984	680	<0.0001

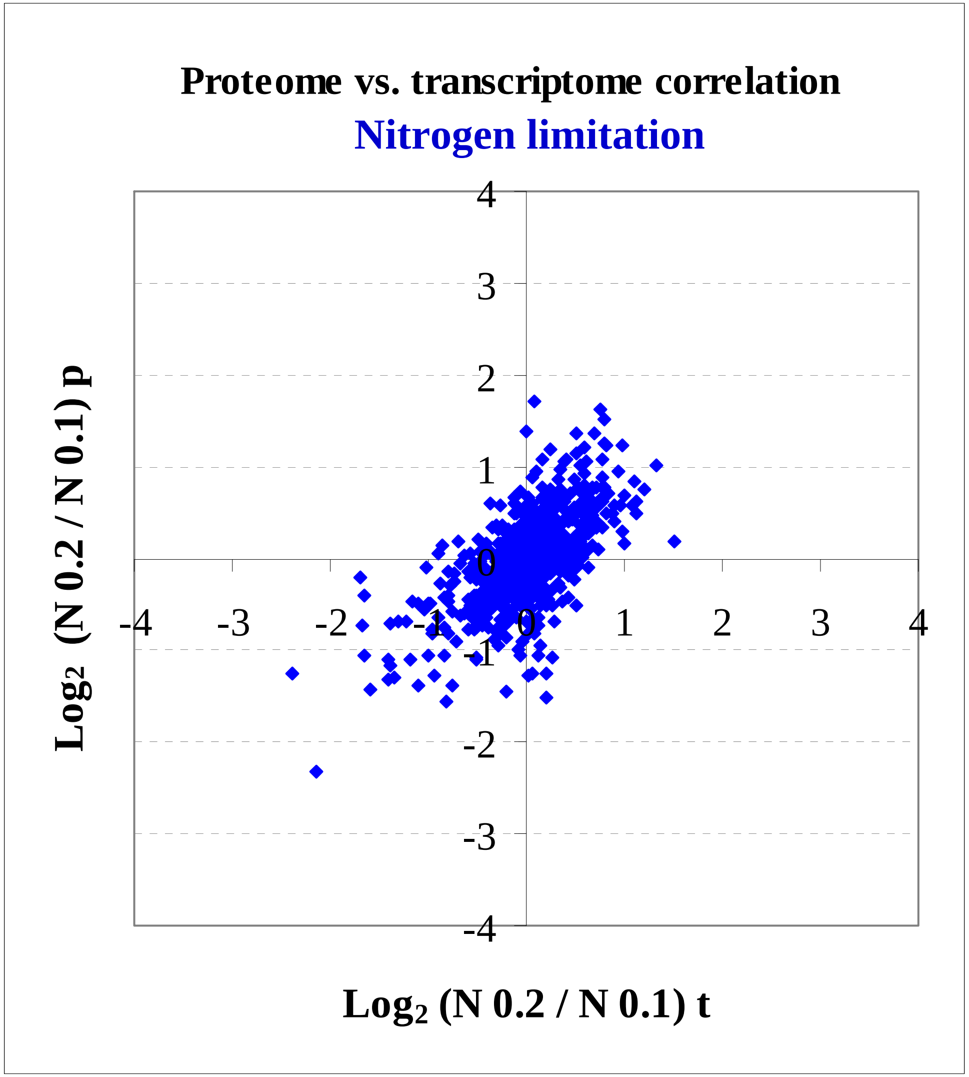

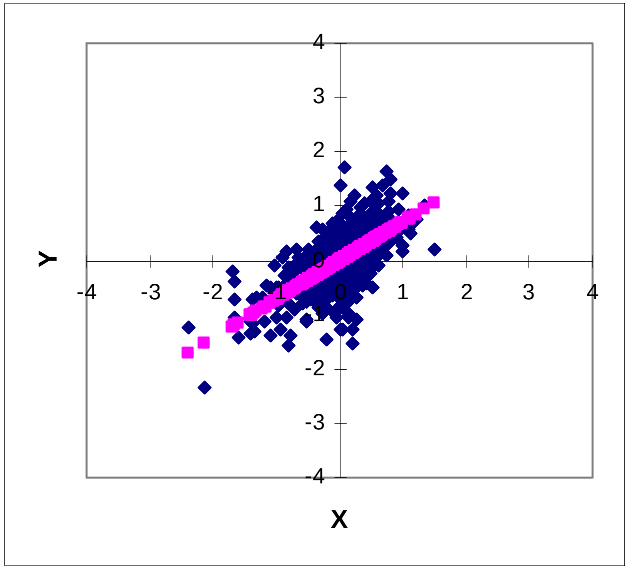

Linear Regression
Y = A + B * X

Parameter	Value	Error
A	-0.01989	0.01496
B	0.71071	0.03167

R	SD	N	P
0.65283	0.39005	680	<0.0001

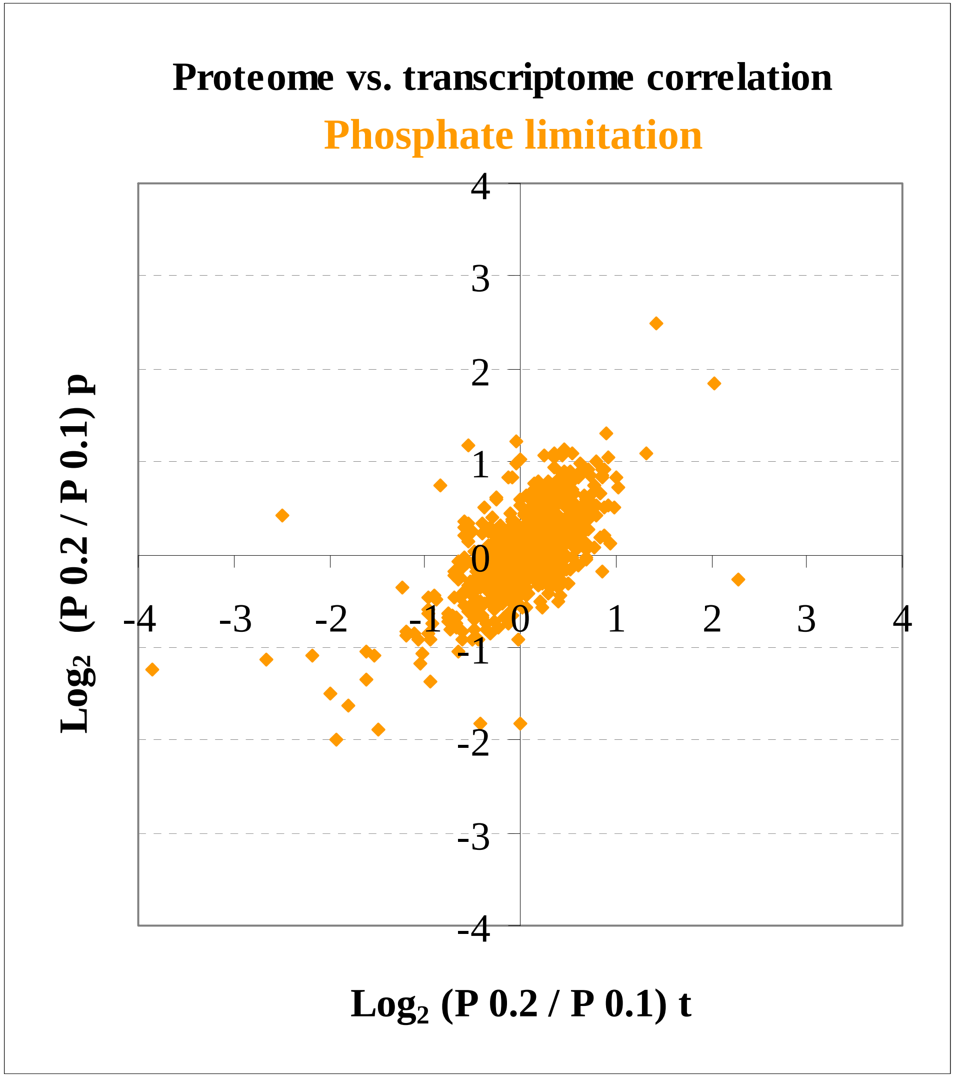

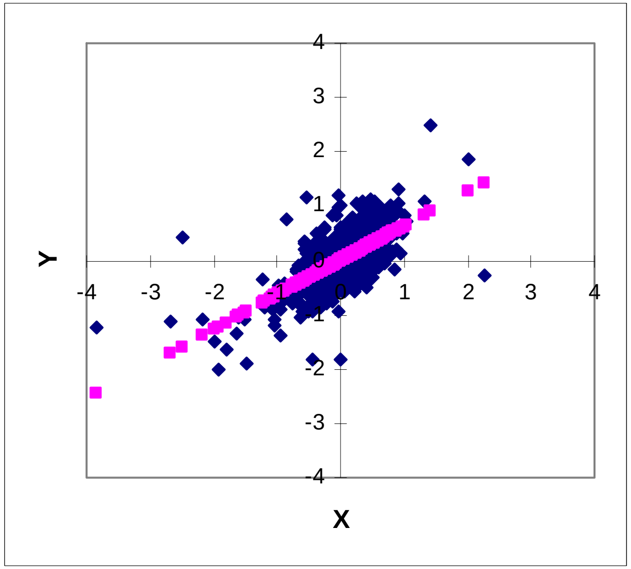

Linear Regression
Y = A + B * X

Parameter	Value	Error
A	-0.00798	0.0128
B	0.63345	0.02645

R	SD	N	P
0.65166	0.35578	779	<0.0001

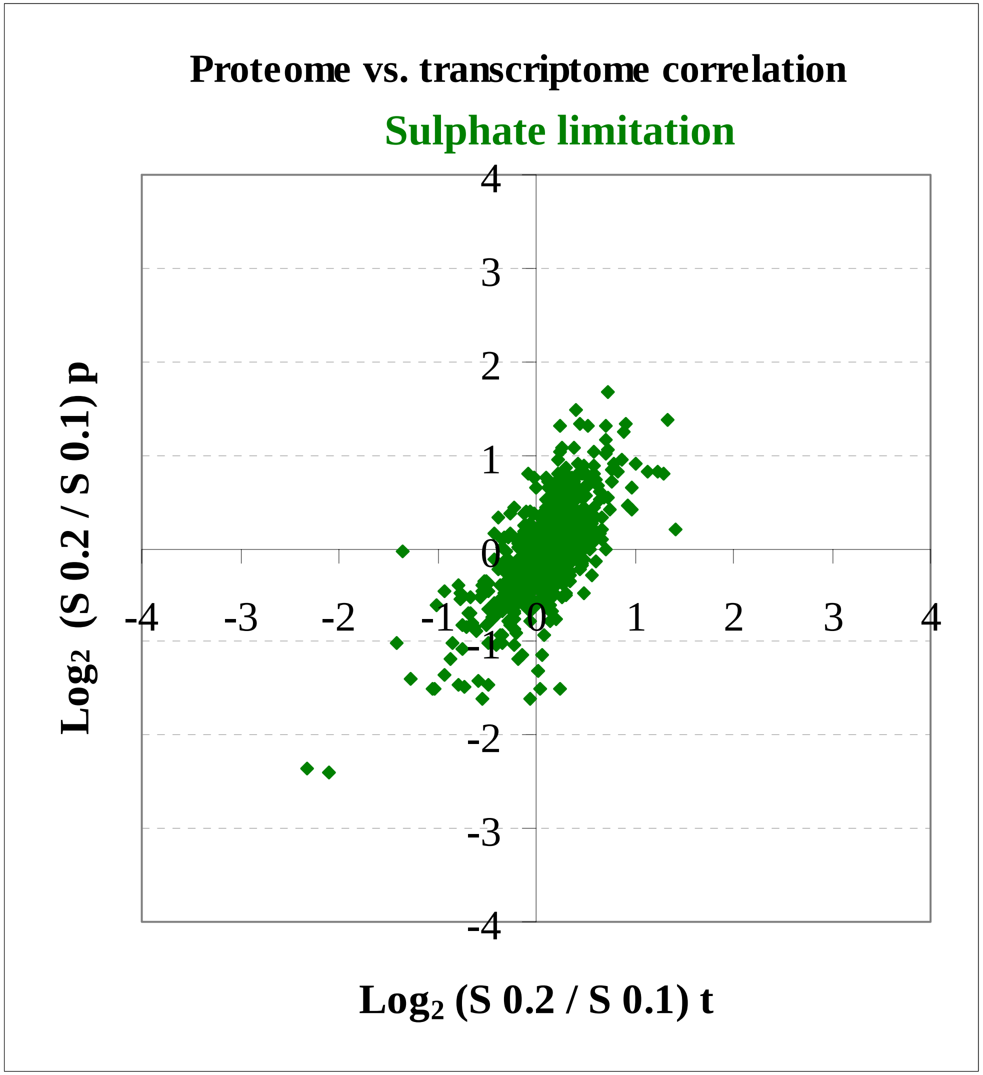

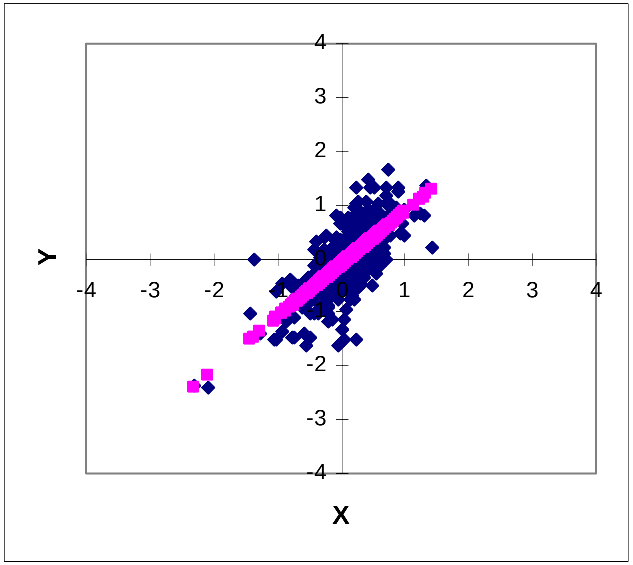

Linear Regression
Y = A + B * X

Parameter	Value	Error
A	-0.12263	0.01505
B	0.98346	0.03824

R	SD	N	P
0.70829	0.37444	659	<0.0001

Proteome – transcriptome correlations

(relative changes from 0. 1 to 0.2 h^{-1})

(natural values)

Graphs and linear regression analysis

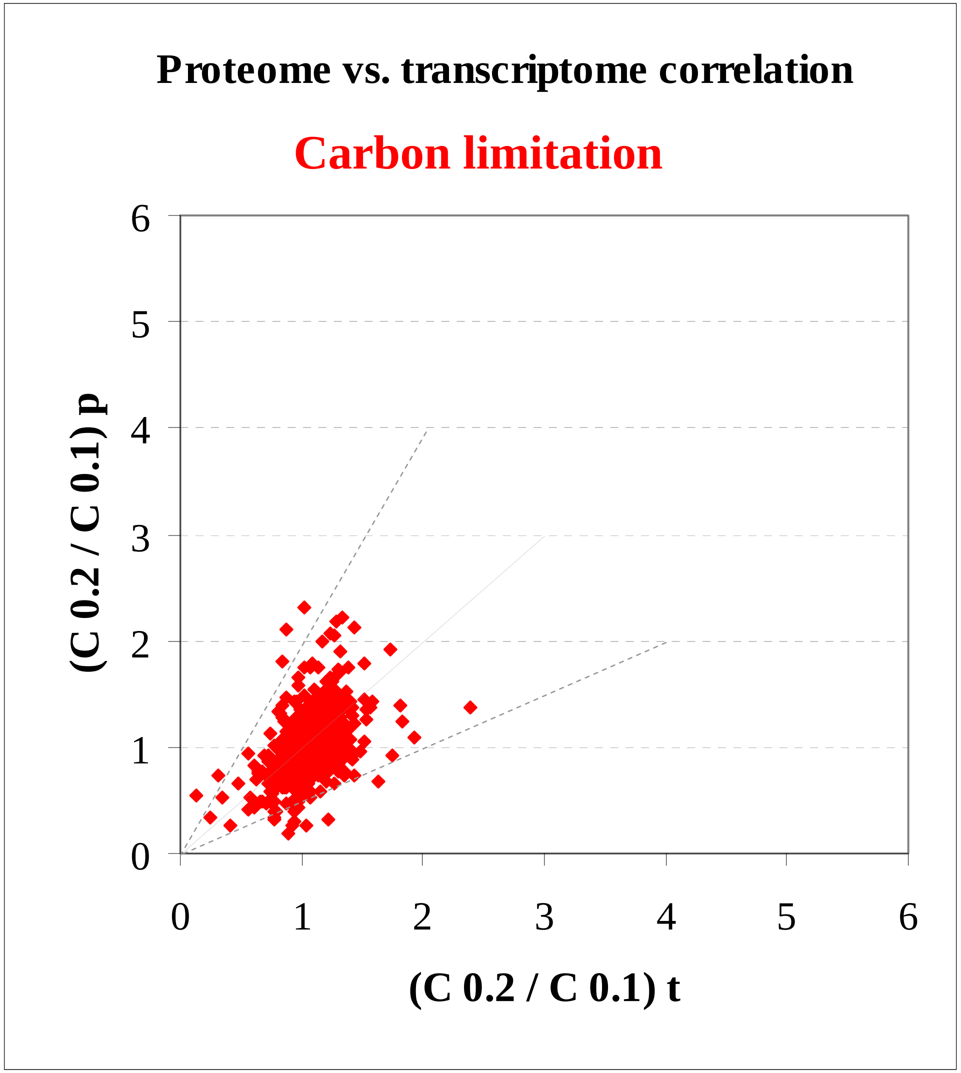

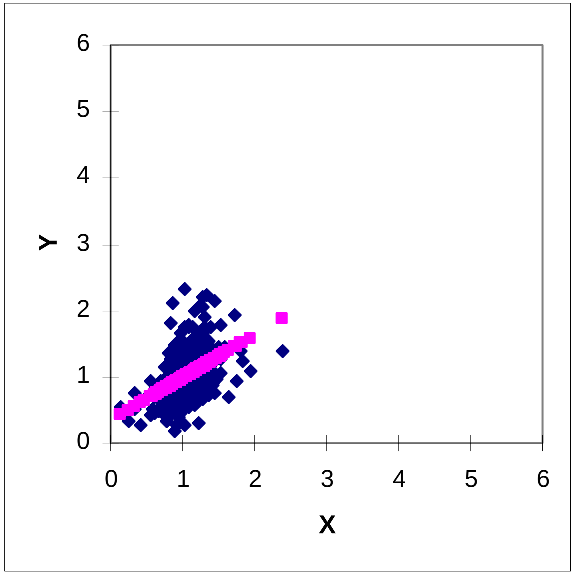

Linear Regression
Y = A + B * X

Parameter	Value	Error
A	0.33048	0.05403
B	0.64153	0.0496

R	SD	N	P
0.44485	0.26168	680	<0.0001

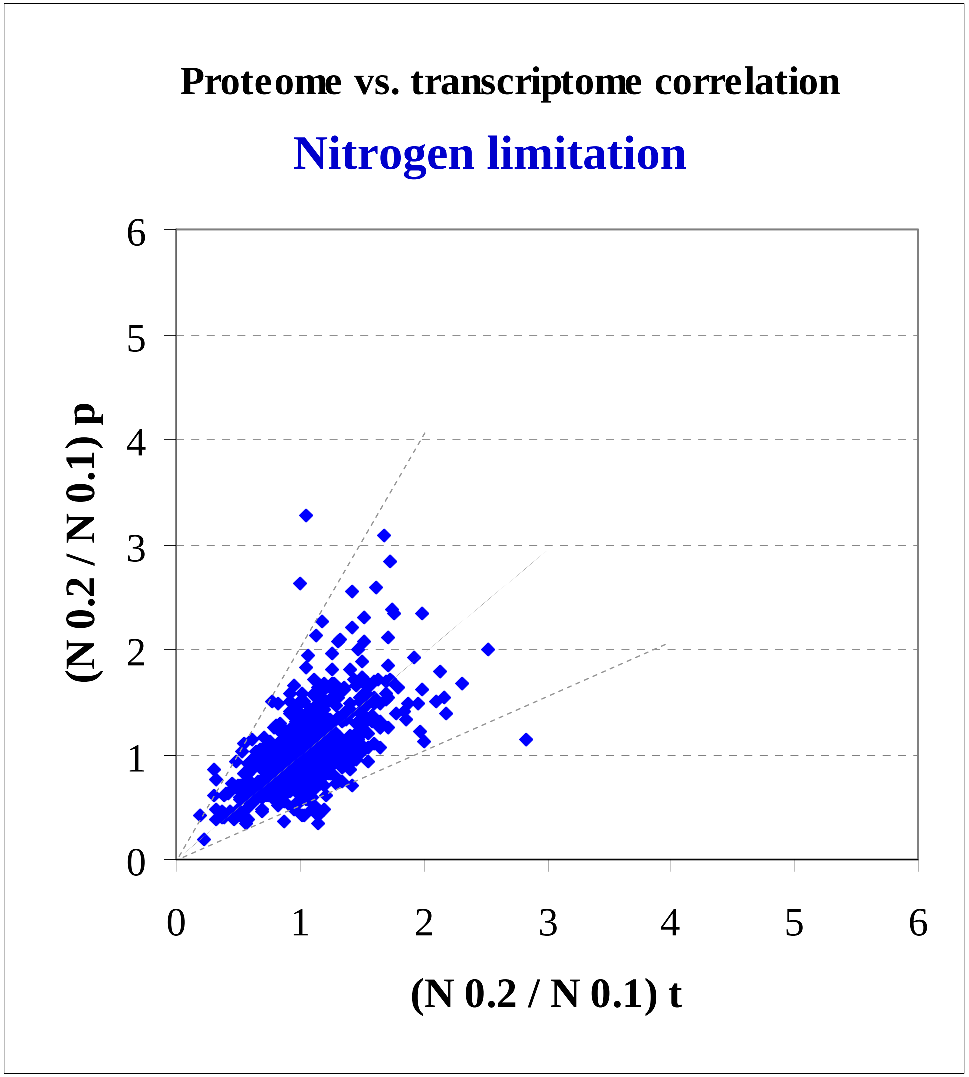

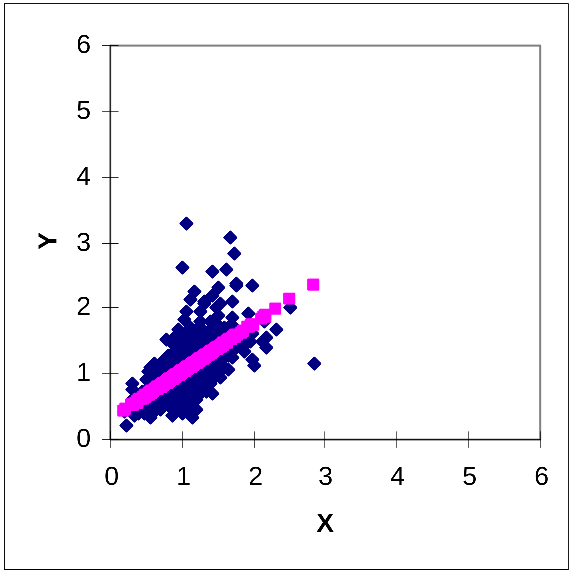

Linear Regression
Y = A + B * X

Parameter	Value	Error
A	0.28149	0.03955
B	0.73136	0.0358

R	SD	N	P
0.61726	0.30703	680	<0.0001

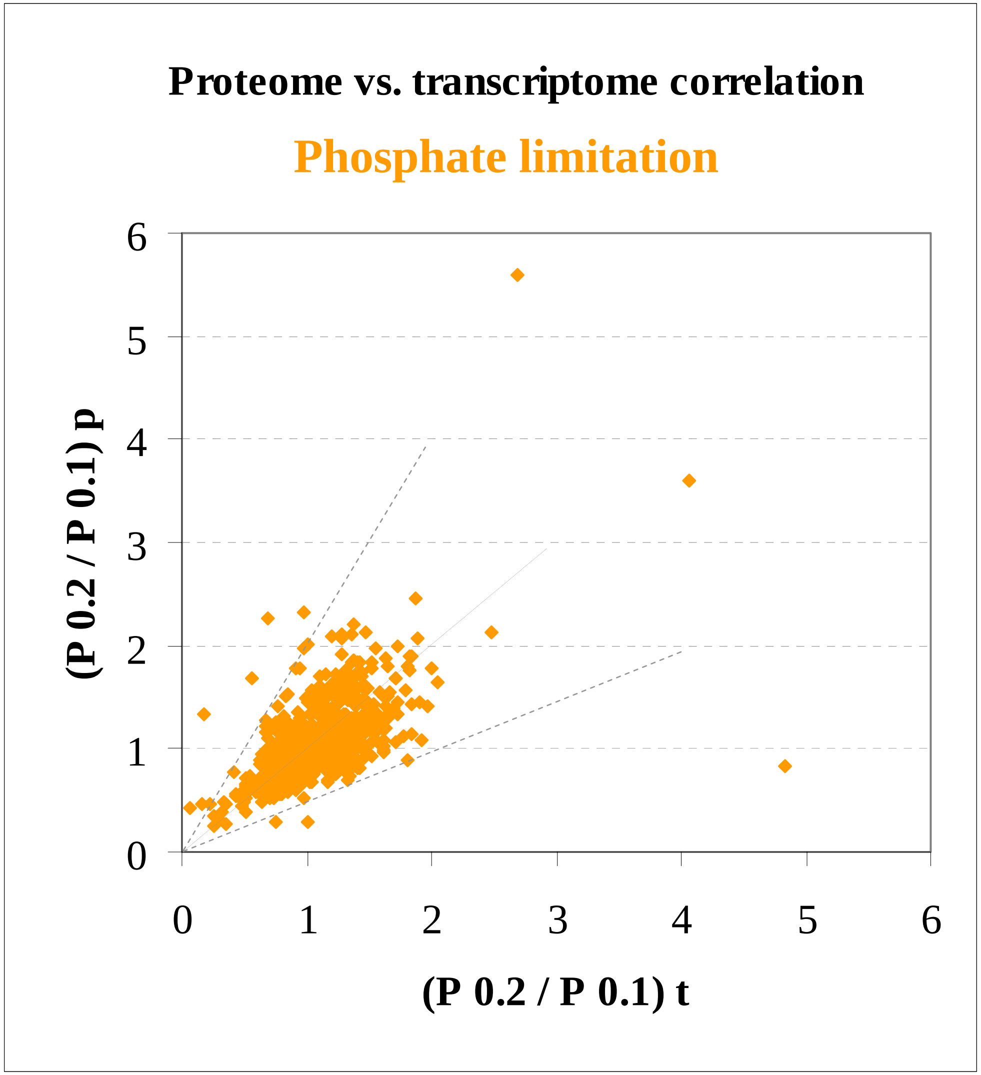

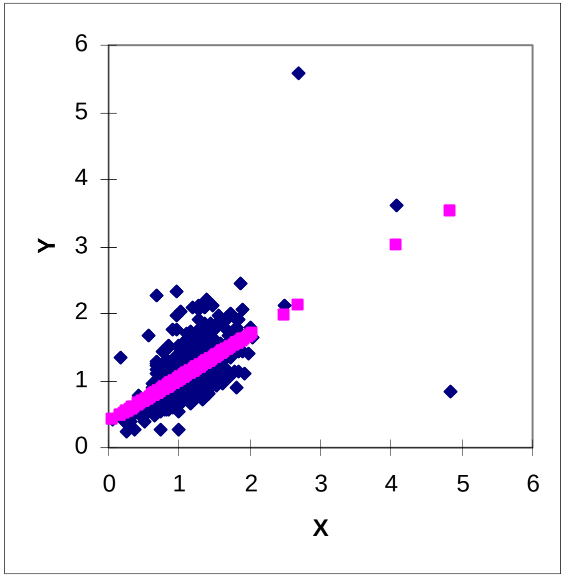

Linear Regression
Y = A + B * X

Parameter	Value	Error
A	0.364	0.03556
B	0.65049	0.03125

R	SD	N	P
0.59839	0.3045	779	<0.0001

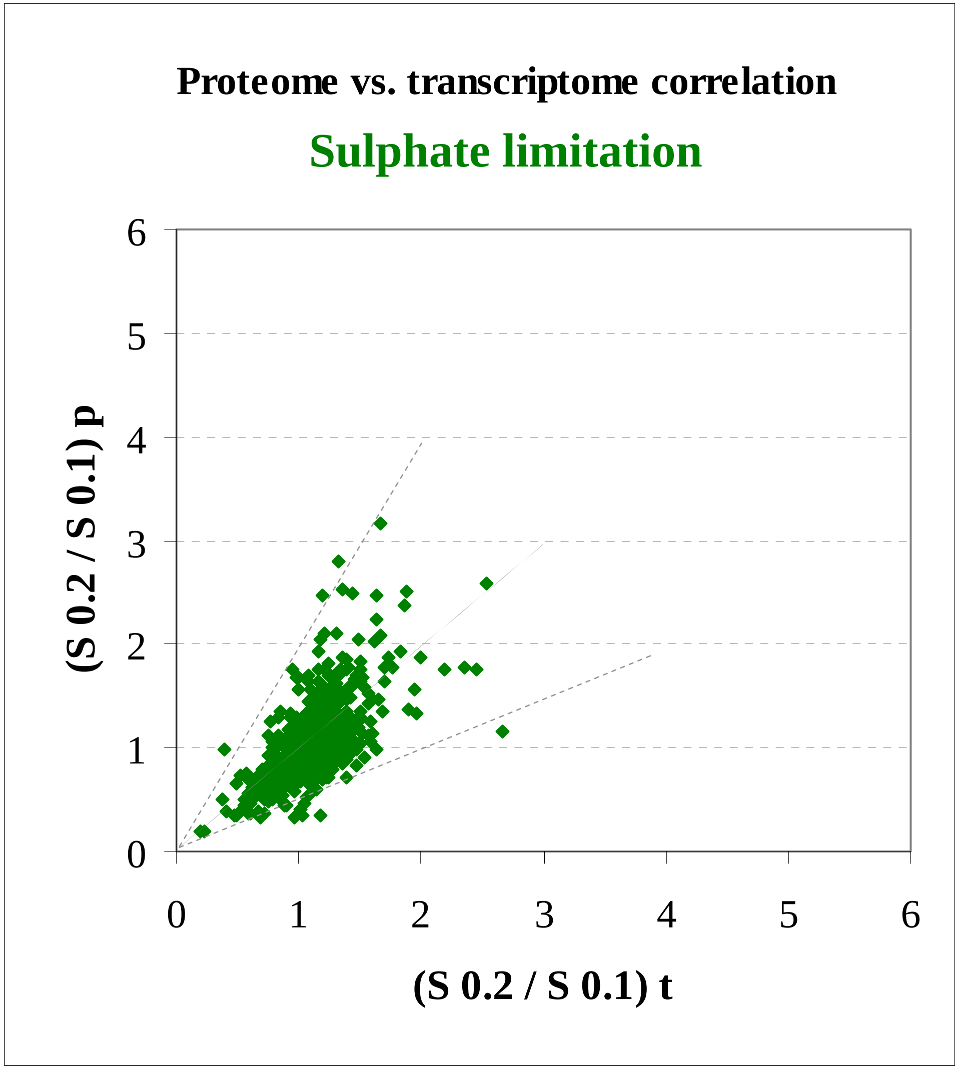

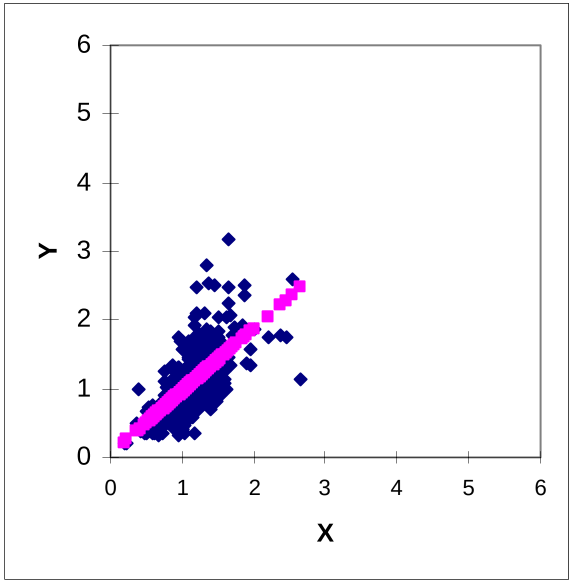

Linear Regression
Y = A + B * X

Parameter	Value	Error
A	0.03376	0.04614
B	0.91729	0.04048

R	SD	N	P
0.66237	0.2903	659	<0.0001
