# Supplementary material for: Growth control of the eukaryote cell: a systems biology study in yeast
Source: J Biol. 2007 Apr 30;6(2):4. doi: 10.1186/jbiol54 (PMC2373899; doi:10.1186/jbiol54)
Supplement: Additional data file 7 — Main concepts on translational control efficiency and translational control [file jbiol54-S7.pdf]

Additional document 7

Translational control efficiency. Ratios. Translational control

- Quantitative genomic strategies. Absolute and relative magnitudes.
- Translational control efficiency (Trlc. Eff.) $_i$. Definition.
- Proteome-transcriptome correlations.
- Ratio relative changes in protein levels vs. relative changes in transcript levels. Ratio [(/) p / (/) tr.] $_i$
- Ratio of translational efficiencies (Ratio of Trlc. Eff.) $_i$. Translational control.

Quantitative genomic studies. Strategies

1) Absolute ‘omic levels (pools)

Example: ORF/gene i: Transcriptome and proteome levels

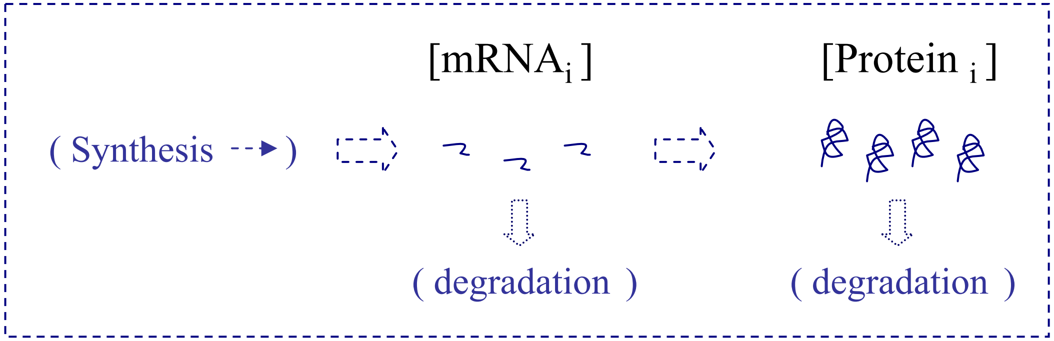

Definition: (**Translational control efficiency**) $_i$ = ([Protein $_i$]) / ([mRNA $_i$])

(Trlc. Eff.) $_i$ (per transcript)

(protein / mRNA) ratio;
actual levels/pools in the cell)

(Effective conversion into protein, encompassing synthesis and degradation processes)

[Absolute concentrations (pools), and translational efficiencies difficult to quantify. Being object of increasing investigation]

(Beilharz and Preiss, 2004; Arava *et al.*, 2005; Mata *et al.*, 2005)

Quantitative genomic studies. Strategies

2) Studies of relative changes in 'omic levels

Example: From condition '1' to '2':
ORF/gene i: Transcript change: 2 fold → Protein change: 3 fold

Example: ORF/gene i

(1) (2) | (1) (2)

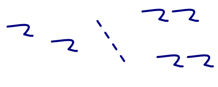

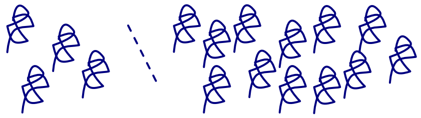

$$\frac{[mRNA_i]_2}{[mRNA_i]_1} = ([mRNA_i]_2 / [mRNA_i]_1)$$

$$\frac{[Protein_i]_2}{[Protein_i]_1} = ([Protein_i]_2 / [Protein_i]_1)$$

(e.g. Microarrays) (e.g. 2D-DIGE; iTRAQ)

Proteome - transcriptome correlation studies

Relative changes in proteome levels vs. relative changes at the transcriptional level.
$[([Protein_i]_2 / [Protein_i]_1) \text{ vs. } ([mRNA_i]_2 / [mRNA_i]_1)]$

Proteome - transcriptome correlation studies

Correlations. Graphical representations

" Relative changes in proteome levels vs. relative changes in transcriptional levels "

A) Correlations. Log Log data

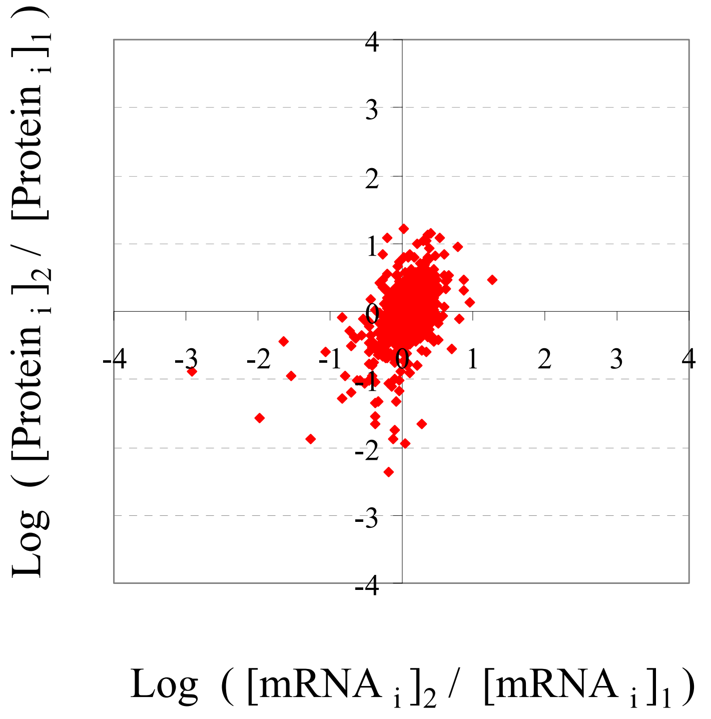

B) Correlations. Natural data

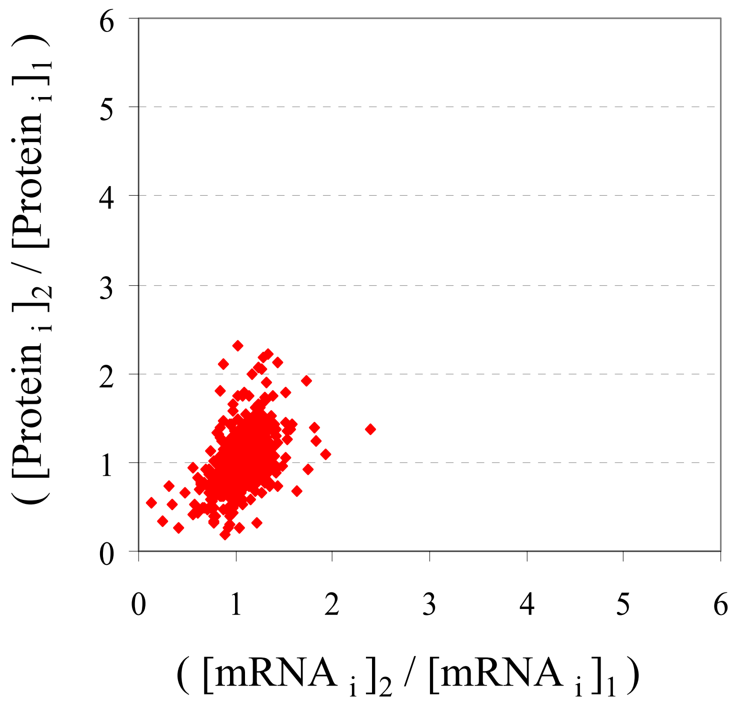

Proteome - transcriptome correlations. Ratios. Translational control

Let's define:

Ratio 'relative change in protein levels vs. relative change in transcript levels' (per ORF):

Ratio [(/) p / (/) tr.]$_i$ = ([Protein$_i$]$_2$ / [Protein$_i$]$_1$) **/** ([mRNA$_i$]$_2$ / [mRNA$_i$]$_1$) **[1]**

This can be rearranged:

= ([Protein$_i$]$_2$ / ([mRNA$_i$]$_2$) **/** ([Protein$_i$]$_1$ / [mRNA$_i$]$_1$)

which is equal to: --> = [(Translational ctrl. efficiency)$_i$]$_2$ / [(Translational ctrl. efficiency)$_i$]$_1$

(see page 2)

= **Ratio of translational control efficiencies$_i$ from condition 1 to 2 = (Ratio of Trlc. Eff.)$_i$**

That is, the Ratio [(/) p / (/) tr.]$_i$, which can be obtained by integrative proteome-transcriptome studies, equals numerically to the ratio of translational efficiencies (per ORF) from condition 1 to 2. This opens the way towards:

↓

Genome-wide studies of changes in translational control efficiencies (from condition 1 to 2)
(e.g. genome-wide studies of relative changes in translational control efficiencies with growth rate).

Proteome - transcriptome correlations. Ratios. Translational control

Calculation of Ratio [(/) p / (/) tr.]$_i$

Ratio [(/) p / (/) tr.]$_i$ = ([Protein $_i$]$_2$ / [Protein $_i$]$_1$) **/** ([mRNA $_i$]$_2$ / [mRNA $_i$]$_1$)

From iTRAQ experiments:

([Protein $_i$]$_2$ / [Protein $_i$]$_1$) raw data → logged for proper statistical analysis + normalization (see methods) . -- > **Normalized log$_2$ ([Protein $_i$]$_2$ / [Protein $_i$]$_1$)** values = **(Y$_i$)**

(or, = log$_2$ [Protein $_i$]$_2$ - log$_2$ [Protein $_i$]$_2$)

From Microarray experiments:

([mRNA $_i$]$_2$ / [mRNA $_i$]$_1$) raw data → logged for proper statistical analysis + normalization (see methods) . -- > **Normalized log$_2$ ([mRNA $_i$]$_2$ / [mRNA $_i$]$_1$)** values = **(X$_i$)**

(or, = log$_2$ [mRNA $_i$]$_2$ - log$_2$ [mRNA $_i$]$_2$)

$$\textbf{Ratio [(/) p / (/) tr.]}_i = \frac{(\,[\text{Protein}_i]_2 / [\text{Protein}_i]_1\,)}{(\,[\text{mRNA}_i]_2 / [\text{mRNA}_i]_1\,)} = \frac{2^{\wedge}(Y_i)}{2^{\wedge}(X_i)} = \textbf{(Ratio of Trlc. Eff.)}_i$$

Appendix: The **Ratio [(/) p / (/) tr.]$_i$** (using natural values, --> ratio from graph B, page 4) has physiological significance, providing direct information of those transcripts whose translational efficiency is changed from condition 1 to 2, their expression being regulated at the translational level.

Question: Does the ratio **Log [(/) p]$_i$ / Log [(/) tr.]$_i$** (ratio from graph A, page 4) have also physiological significance?

Log [(/) p]$_i$ / Log [(/) tr.]$_i$ =

$$= \text{Log} \left([\text{Protein}_i]_2 / [\text{Protein}_i]_1 \right) \,/\, \text{Log} \left([\text{mRNA}_i]_2 / [\text{mRNA}_i]_1 \right)$$

which can be expressed as:

$$= \left(\text{Log}\, [\text{Protein}_i]_2 - \text{Log}\, [\text{Protein}_i]_1 \right) \,/\, \left(\text{Log}\, [\text{mRNA}_i]_2 - \text{Log}\, [\text{mRNA}_i]_1 \right)$$

but, this **is not** equal to:

$$\neq \left(\text{Log}\, [\text{Protein}_i]_2 - \text{Log}\, [\text{mRNA}_i]_2 \right) \,/\, \left(\text{Log}\, [\text{Protein}_i]_1 - \text{Log}\, [\text{mRNA}_i]_1 \right) =$$
$$= \text{Log} \left([\text{Protein}_i]_2 / ([\text{mRNA}_i]_2 \right) \,/\, \text{Log} \left([\text{Protein}_i]_1 / [\text{mRNA}_i]_1 \right) =$$
$$= \text{Log} \left([\text{ Translational ctrl. efficiency })_i]_2 \,/\, [\text{ Translational ctrl. efficiency })_i]_1 \right)$$

Conclusion: The ratio **Log [(/) p]$_i$ / Log [(/) tr.]$_i$** (from graph A, page 4) has no direct physiological significance.

References

Arava Y, Boas FE, Brown PO, Herschlag D: **Dissecting eukaryotic translation and its control by ribosome density mapping.** *Nucleic Acids Res* 2005, **33**:2421-2432.

Beilharz TH, Preiss T: **Translational profiling: the genome-wide measure of the nascent proteome.** *Brief Funct Genomic Proteomic* 2004, **3**:103-111.

Mata J, Marguerat S, Bähler J: **Post-transcriptional control of gene expression: a genome-wide perspective.** *Trends Biochem Sci* 2005, **30**:506-514.
