# Supplementary material for: Growth control of the eukaryote cell: a systems biology study in yeast
Source: J Biol. 2007 Apr 30;6(2):4. doi: 10.1186/jbiol54 (PMC2373899; doi:10.1186/jbiol54)

Additional document 8

Global distributions of relative changes in translational control efficiencies

Rel. Ch. Trlc. Eff. (relative changes from 0. 1 to 0.2 h^{-1})

(natural values)

Global distribution of relative changes in translational control efficiencies
(Rel. Ch. Trlc. Eff. from μ = 0.1 to 0.2 h^{-1})

Carbon limitation

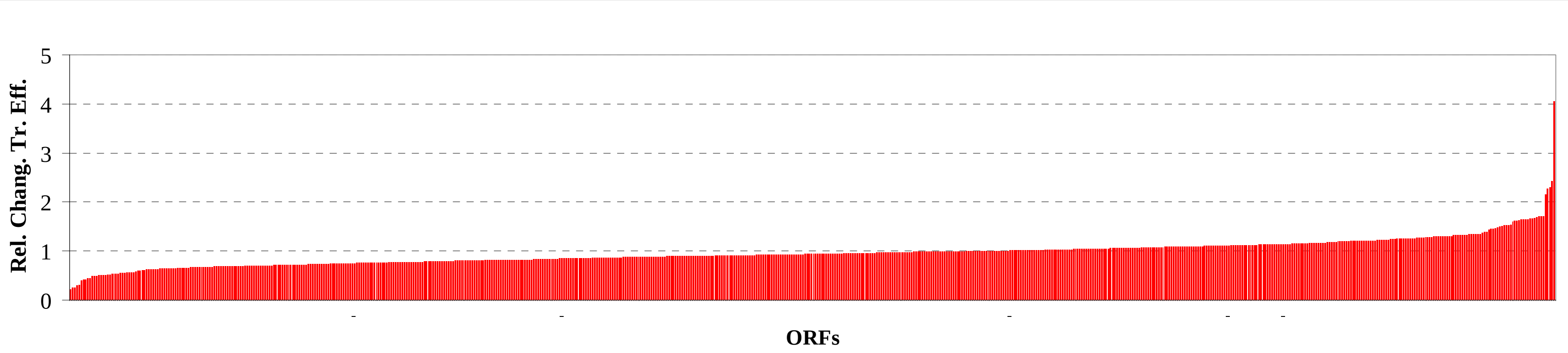

Carbon limitation: From μ = 0.1 to 0.2 h^{-1}

Proportion of transcripts that change their Trlc. Eff. in the range [0.5 - 2] = **97%**

→

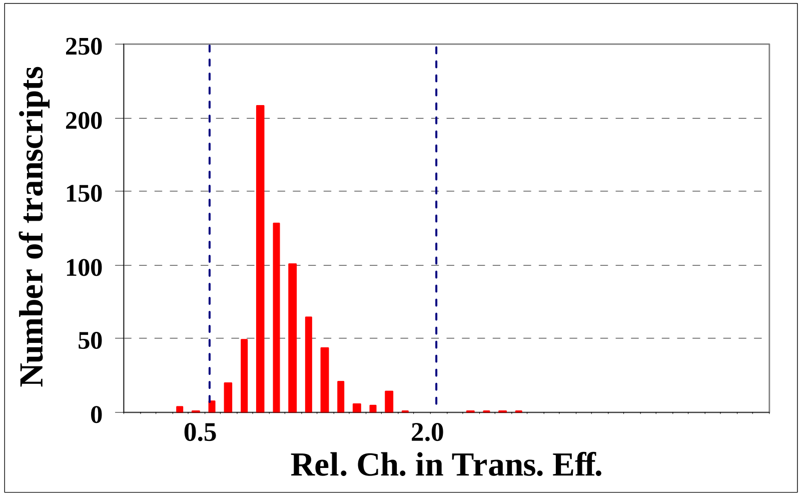

Global distribution of relative changes in translational efficiencies
(Rel. Ch. Trlc. Eff. from μ = 0.1 to 0.2 h^{-1})

Nitrogen limitation

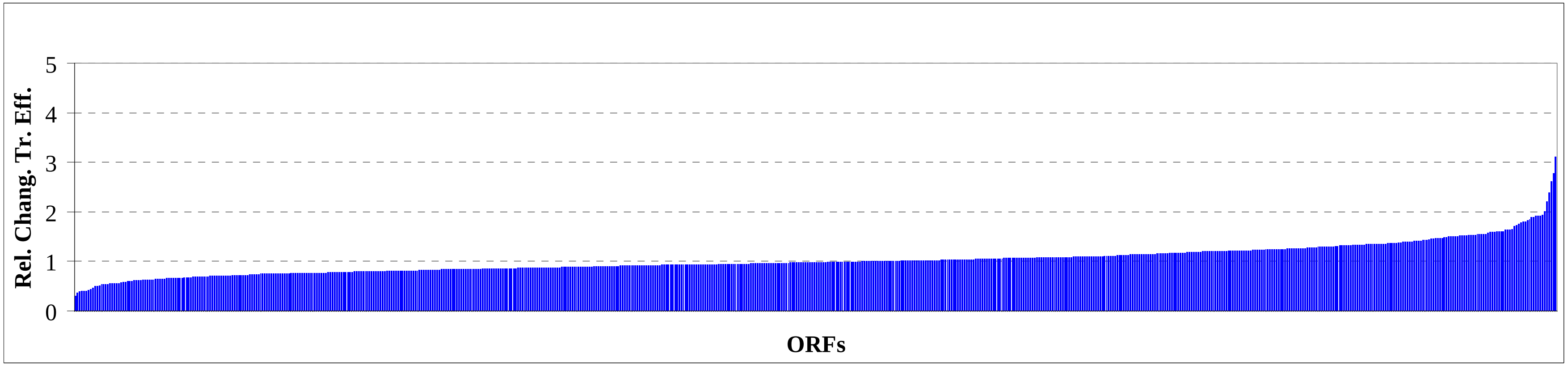

Nitrogen limitation: From μ = 0.1 to 0.2 h^{-1}

Proportion of transcripts that change their Trlc. Eff. in the range [0.5 - 2] = **97%**

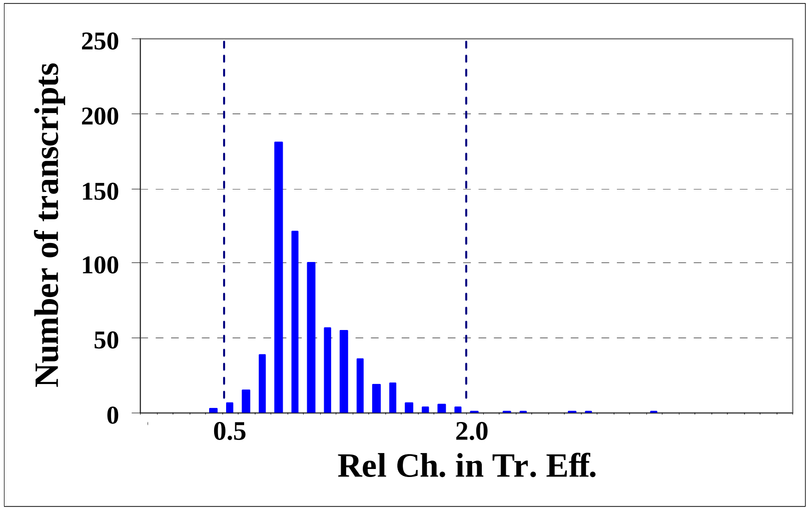

Supplement: Additional data file 8 — Global patterns of relative changes in translational control efficiencies. [file jbiol54-S8.pdf]
